# Unsymmetrical sulfoxides with sterically hindered catechol fragment: synthesis, structure, electrochemical properties, and antiradical activity

**DOI:** 10.3762/bjoc.22.65

**Published:** 2026-06-01

**Authors:** Daria A Burmistrova, Vasiliy A Fokin, Oleg P Demidov, Mikhail A Kiskin, Maxim V Arsenyev, Andrey I Poddel’sky, Nadezhda T Berberova, Ivan V Smolyaninov

**Affiliations:** 1 Chemistry Department, Astrakhan State Technical University, Tatischev str. 16/1, 414056, Astrakhan, Russia; 2 North-Caucasus Federal University, Pushkin str., 1, 355017, Stavropol, Russia; 3 Kurnakov Institute of General and Inorganic Chemistry, Russian Academy of Sciences, Leninskii prosp., 31, 119071, Moscow, Russia; 4 Razuvaev Institute of Organometallic Chemistry, Russian Academy of Sciences, Tropinina str., 49, 603137, Nizhniy Novgorod, Russia

**Keywords:** antioxidant activity, catechol thioethers, redox-transformations, sulfoxides, X-ray analysis

## Abstract

New unsymmetrical sulfoxides containing a redox-active sterically hindered catechol fragment and a nonpolar hydrocarbon substituent at the sulfoxide group were synthesized via the oxidation of the corresponding catechol thioethers with hydrogen peroxide. The yield of the reaction products ranges from 42 to 89%. The crystal structures of catechol sulfoxides with isopropyl, cyclopentyl, adamantyl, benzyl and 1-naphthyl moieties were established by single-crystal X-ray analysis. The possibility of forming intra- and intermolecular hydrogen bonds has been shown for these compounds. The electrochemical behavior of sulfoxides was studied in comparison with that of the parent catechol thioethers. The redox transition corresponding to catechol fragment oxidation is shifted to the anodic region relative to the initial thioethers, which is attributed to the more electron-withdrawing nature of the S=O group. The second redox stage characterizes the transformation of the sulfoxide fragment and is observed in most cases at 1.82–1.91 V. The radical-scavenging activity and antioxidant properties of the unsymmetrical sulfoxides and their precursor thioethers were evaluated using the reaction with the 2,2′-diphenyl-1-picrylhydrazyl radical (DPPH) and the 2,2′-azinobis(3-ethylbenzothiazoline-6-sulfonic acid) radical cation (ABTS^+•^). In both assays, the lowest IC_50_ values among the studied catechol sulfoxides were found for compounds bearing isopropyl and *tert*-butyl substituents on the sulfoxide group.

## Introduction

Polyphenolic compounds can participate in redox processes and undergo a wide range of chemical modifications. As a result, they possess diverse biological activities and unusual physicochemical properties, which render them useful in various fields of chemistry. Catechol-containing compounds, in particular, are known for their antioxidant activity through the regulation of free-radical processes [1–2], and are used as antiparkinsonian [3–5], antitumor, and antibacterial agents [6–8]. However, the practical utility of such type of compounds extends beyond biomedical applications. For instance, Michael addition reactions between dithiols and phenolic compounds yield cross-linked, colorless polymer films with universal adhesion and high stability, imparting resistance to various solvents [9–10]. Furthermore, catechol-containing compounds can serve as antioxidant biomimetic additives in lubricants [11].

One effective approach for the fine-tuning of polyphenol properties is functionalization via the introduction of heteroatoms (S, Se, Te) [12–14]. We have previously shown that hybrid molecules based on sterically hindered catechol thioethers, bearing various polar and hydrocarbon groups, exhibit pronounced chelating [15–16], antiradical, and cryoprotective activity [17–20]. The ability of the sulfur atom to exist in various oxidation states provides a powerful way to control organic molecules. Through the controlled conversion of thioethers to sulfoxides and sulfones [21], it is possible to tune their reactivity and biological properties. Indeed, compounds of this type possess pronounced neuroprotective properties [12–14]. However, despite significant progress in the study of catechols with sulfide linkers, the chemistry of sulfoxides and sulfones is still a relatively unexplored area.

Polyfunctional catechol thioethers are most commonly synthesized via the Michael reaction between *o*- or *p*-benzoquinone and the corresponding thiol [22–25], through nucleophilic aromatic substitution on the catechol ring [26–27], or under electrochemical conditions [28–32]. A number of methods exist for the conversion of sulfides into sulfoxides, proceeding via either oxidative or non-oxidative pathways. The controlled chemoselective oxidation of the thioether group to the sulfoxide, without overoxidation to the sulfone, remains a current challenge in organic chemistry. Peroxide compounds – such as hydrogen peroxide [33], organic hydroperoxides [34–35], peracids [36], ozone, and the peroxymonosulfate anion (the active component of Oxone^®^) [37] – are commonly employed for this purpose. One of the most accessible approaches uses hydrogen peroxide along with catalytic systems based on transition metals [38]. Notably, this process is not stereoselective and affords chiral sulfoxides as a mixture of enantiomers. Besides environmentally friendly and readily available hydrogen peroxide, oxygen is another soft “green” oxidizer widely used in the oxidation of sulfides to sulfoxides [39–41]. In the case of catechol thioethers, an additional key challenge lies in achieving chemoselective oxidation of the sulfur atom in the presence of the readily oxidizable catechol core.

The presence of a redox-active catechol moiety adjacent to the sulfoxide group can significantly influence the formation of intra- and intermolecular hydrogen bonds and affect the O–H bond dissociation energy, which in turn may substantially impact the antioxidant properties. Moreover, the combination of a polar sulfoxide group and a nonpolar hydrocarbon tail allows the lipophilic–hydrophilic balance of the molecule to be fine-tuned, a factor critically important for bioavailability. The incorporation of both a rigid O,O′-chelating catechol fragment and a soft sulfoxide group within a single molecule also provides a basis for the preparation of metal complexes with unusual properties. The aim of the present work is to synthesize new unsymmetrical sulfoxides bearing hydrocarbon fragments derived from catechol thioethers, to study their structure and electrochemical properties, and to establish how the hydrocarbon substituent influences antiradical activity upon conversion from thioethers to sulfoxides.

## Results and Discussion

### Synthesis

**Scheme 1:**
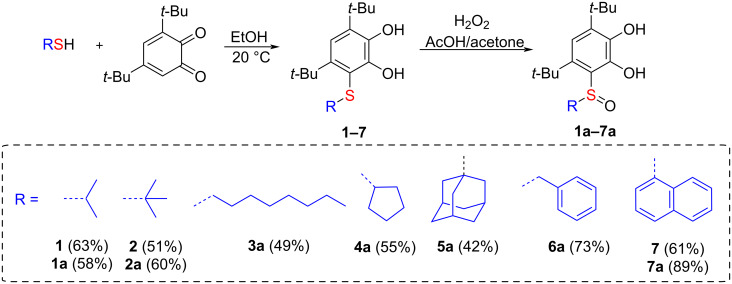
Synthesis of catechol-contained thioethers **1–7** and sulfoxides **1a–7a**.

The starting unsymmetrical thioethers **1**–**7** were obtained according to a previously described procedure based on the Michael reaction (Scheme 1) [23,42]. When converting these thioethers into sulfoxides using hydrogen peroxide as the oxidant, there is a risk of concurrent oxidation of the catechol moiety. The oxidation of 3,5-di-*tert*-butylcatechol (3,5-DTBC) by hydrogen peroxide is a classic reaction in bioinorganic chemistry, frequently employed as a model system to study the activity of catechol oxidase and tyrosinase. Although hydrogen peroxide is a strong oxidant, its reaction with 3,5-DTBC is often slow at room temperature in the absence of a catalyst, typically requiring metal ions such as Cu^2+^, Fe^3+^, or Co^2+^ [43]. Moreover, the oxidation of catechols is facilitated under alkaline conditions. Therefore, the oxidation of thioethers **1**–**7** was carried out in the presence of acetic acid (Scheme 1). This approach proved successful for the selective oxidation of the thioether group, and no products arising from oxidation of the catechol moiety were detected. The yields of the target sulfoxides ranged from 42% to 89%.

Compounds **3**–**6** were prepared and characterized as described previously (**3**, **4**, and **6** [23], **5** [42]). The structures of new thioethers **1**, **2**, **7** and sulfoxides **1a**–**7a** were confirmed by the spectral methods IR-, ^1^H NMR, ^13^C{^1^H} NMR spectroscopy (Figures S1–S20), HRMS (Figures S21–S26) in Supporting Information File 1, and elemental analysis.

### X-ray data

The X-ray suitable crystals of **1a**, **4a**–**7a** were grown by slow recrystallization of the compounds from acetonitrile or chloroform (for **7a**) solutions at room temperature. The crystallographic parameters and X-ray diffraction experimental parameters are given in Supporting Information File 1, Table S1. Compound **4a** crystallizes as a solvate with hexane in a ratio of two crystallographically independent molecules. In the monoclinic crystal of compound **5a**, two independent molecules were refined.

**Figure 1:**
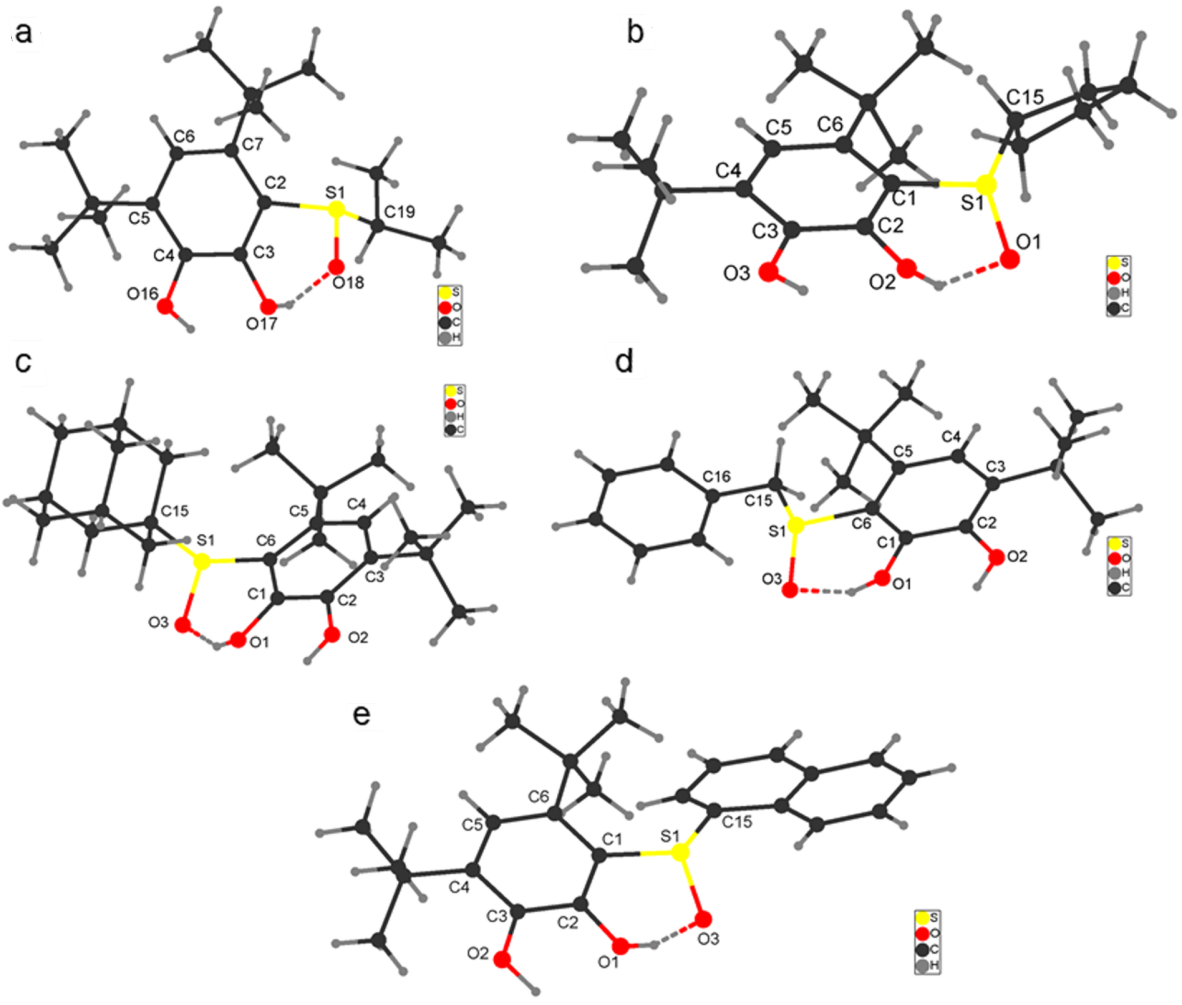
Molecular structures of **1a** (a), **4a** (b), **5a** (c), **6a** (d), **7a** (e) (solvent molecules and fragment disordering are omitted; for **4a** and **5a**, only one independent molecule is shown).

In all the studied single-crystal compounds, the molecules (Figure 1) contain a catechol fragment, in which the bond lengths in the phenyl ring, hydroxy and *tert*-butyl groups (Table S2, Supporting Information File 1) correspond to the literature data for catechols [18,44].

The observed S=O distances range from 1.512 to 1.532 Å, which in most compounds except **4a** is longer than the average sulfoxide bond length of 1.489–1.515 Å [45–46] and close to the bond length in DMSO [47]. The S–C(cat) bond distances range in the narrow range of values 1.782–1.789 Å. The S–C(R) distance varies in the range of 1.838–1.892 Å. If the C(R) atom is aromatic as in the case of **7a**, the average S–C distance has a tendency to be slightly shorter (1.804 Å) due to a possibility of weak conjugation of the π-system in the aromatic ring with the S=O double bond, but nevertheless close to the corresponding value for **6a**. The O–S–C(cat) and O–S–C(R) bond angles vary in the ranges of 104.2(2)–106.75(7)° and 103.6(3)–110.0(3)°, respectively. The torsion angle (C(R)–S–C(cat)–C(OH)) between the plane of the aromatic ring of the catechol fragment and the hydrocarbon group at the sulfur atom ranges from 66.7° to 84.3°, depending on the nature of the substituent.

The hydroxy group at the second position participates in the formation of an intramolecular H-bond with the oxygen atom of the sulfoxide group (Figure 1), and the second OH group forms an intermolecular contact with the SO group of the neighboring molecule (Table S3, Supporting Information File 1). The crystal packing is also determined by C–H···O, C–H···S, O–H···S, and C–H···π non-covalent interactions (Tables S3 and S4, Supporting Information File 1). In the crystal of **7a**, the naphthyl aromatic fragments of neighboring molecules are located at a close distance, indicating the formation of π contacts (C_g_···C_g_ 3.676(9)–3.769(9) Å).

In the ^1^H NMR spectra of all synthesized sulfoxides, the signal corresponding to the proton of the hydroxy group at position 1 of the aromatic ring is significantly shifted downfield relative to that in the corresponding thioethers (δ 7.11–7.30 ppm) and appears in the range of δ 11.33–11.85 ppm (CDCl₃; see Supporting Information File 1, Section S2). The signal of the second OH proton is also shifted downfield compared to that in the thioethers (δ 5.53–5.58 ppm) and is observed at δ 6.04–6.17 ppm. In the ^13^C NMR spectra of the sulfoxides, the signals of the carbon atoms bearing the OH groups are shifted downfield and appear at δ 148.4–150.0 ppm. The sulfinyl group also influences the signals of aliphatic carbon atoms. For example, in sulfoxide **1a**, the signal of the secondary carbon in the isopropyl substituent is shifted downfield (δ 54.4 ppm) compared to the analogous carbon atom in thioether **1** (δ 41.9 ppm). These results indicate a pronounced electron-withdrawing effect of the sulfinyl group, as well as the presence of strong intra- and intermolecular O–H···O=S bonds, which is consistent with the IR spectroscopic data.

In the IR spectra of the sulfoxides, bands corresponding to the stretching vibrations of hydrogen-bonded OH groups are observed. For compounds **2a**–**4a**, **6a**, and **7a**, a single broad band appears in the region of 3223–3465 cm^−1^, whereas for **1a** and **5a**, two bands are fixed at 3418/3368 cm^−1^ and 3276/3509 cm^−1^, respectively. For most compounds, these bands are slightly shifted to lower wavenumbers by 22–62 cm^−1^ relative to the corresponding thioethers. The largest shifts, ranging from 146 to 180 cm^−1^, are registered for compounds **6a** and **7a**, which contain benzyl and naphthyl substituents, respectively. A notable feature of the IR spectrum of sulfoxide **5a** is the smallest shift of the OH bands (6–13 cm^−1^) relative to the corresponding thioether, which is attributed to the bulky adamantyl substituent. Consequently, differences in the strength of hydrogen bonding among the synthesized compounds are evident.

The presence of sufficiently strong O–H···O=S interactions also results in a shift of the S=O stretching vibration to lower wavenumbers (945–949 cm^−1^) relative to the typical values reported for non‑hydrogen‑bonded sulfoxides, in agreement with literature data [32].

### Electrochemical properties

A comparative assessment of the electrochemical properties of the synthesized catechol sulfoxides and their precursors thioethers enables the proposal of an electrooxidation mechanism, the identification of electron transfer centers, and the elucidation of differences in the redox behavior of catechols containing sulfur atoms in various oxidation states. Furthermore, a detailed study of the redox properties of functionalized catechols allows the prediction of their antioxidant activity based on electrochemical data. The redox properties of catechol-thioethers **1**–**7** (CatH_2_-S-R) and sulfoxides **1a**–**7a** (CatH_2_-SO-R) were studied by cyclic voltammetry (CV) (Table 1). All experimental electrochemical methods and conditions are presented in Supporting Information File 1.

**Table 1:** Peak (*E*_p_^ox^) potentials of **1**–**7** and **1a**–**7a** obtained by CV (GC-electrode, *d* = 2 mm, CH_3_CN, *ν* = 0.2 V∙s^−1^, 0.1 М *n*-Bu_4_NClO_4_, *c* = 3 mmol·L^−1^, Ag/AgCl/KCl (sat.)).

Compound	*E*_p_^ox1^, V	*E*_p_^ox2^, V

CatH_2_-S-R		

**1**	1.21	1.61
**2**	1.23	1.63
**3** ^a^	1.20	1.59
**4** ^a^	1.21	1.59
**5** ^b^	1.19	1.63
**6** ^a^	1.18	1.54
**7**	1.24	1.55

CatH_2_-SO-R		

**1a**	1.25	1.90
**2a**	1.24	1.60
**3a**	1.26	1.91
**4a**	1.25	1.90
**5a**	1.19	1.51
**6a**	1.27	1.90
**7a**	1.26	1.82

^a^Data were obtained from [23]; ^b^data were obtained from [42].

**Figure 2:**
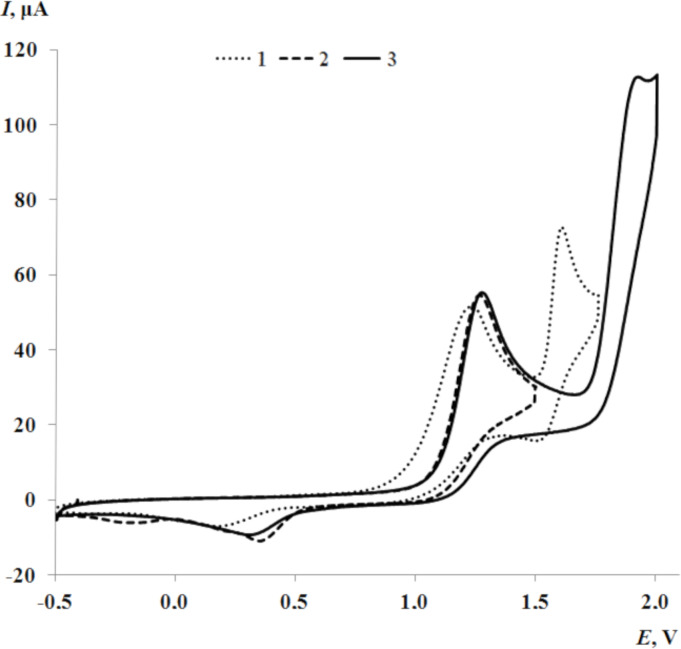
CV curves of **1** and **1a** at the potential ranges: from −0.5 to 1.75 V for **1** (curve 1); from −0.5 to 1.5 V for **1a** (curve 2); from −0.5 to 2.0 V for **1a** (curve 3); (CH_3_CN, GC electrode, Ag/AgCl/KCl(sat.), 0.15 M *n*-Bu_4_NClO_4_, *c* = 3 mmol·L^−1^).

In the anodic area, the electrochemical profile of thioethers **1**, **2**, and **7** exhibit two successive oxidative stages consistent with previous data for compounds **3**–**6** [23,42]. The first two-electron peak (*E*_p_^ox1^) observed for **1**–**7** in the range of 1.19–1.24 V (Figure 2 (curve 1), Figure S27 in Supporting Information File 1) corresponds to the catechol moiety oxidation to the *o*-benzoquinone (Cat-S-R) [23,48]. The second quasi-revisable peak (*E*_p_^ox2^) characterizes the oxidation of the sulfide fragment (1.54–1.63 V). The values of current ratios (*I_c_/I_a_*) are in the range of 0.3–0.7 and suggest the formation of low-stable intermediates.

The electrooxidation of sulfoxides **1a**–**7a** proceeds similarly in two or three consecutive steps in acetonitrile depending on the structure of the hydrocarbon substituent (Figure 2, curves 2–3; Figures S28–S32 in Supporting Information File 1). For compounds **1a**–**7a**, the first irreversible two-electron redox transition is observed at 1.19–1.27 V and corresponds to the oxidation of the catechol moiety. The presence of the electron-withdrawing sulfinyl group in **1a**–**4a**, **6a**, and **7a** leads to a slight shift of *E*_p_^ox1^ to the anodic region by 0.01–0.09 V compared to the corresponding thioethers and by 0.08–0.16 V relative to 3,5-DTBC (1.11 V) [23].

**Scheme 2:**
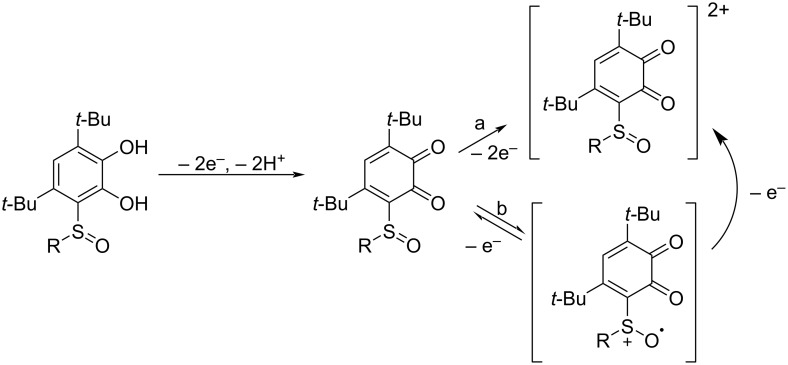
Proposed mechanism of electrochemical transformations of catechol sulfoxides (path a *–* for **1a**, **3a**, **4a**, **6a**, and **7a**; path b *–* for **2a** and **5a**).

It is worth noting that for the pair of compounds **5** and **5a**, bearing an adamantyl substituent at the sulfur atom, no pronounced effect of the S=O group on the *E*_p_^ox1^ value is displayed. This behavior may be explained by a compensatory effect arising from the formation of intramolecular O–H···O=S hydrogen bonds, which results in a partial shift of electron density from the hydrogen atom to the oxygen of the sulfinyl group. This weakens the O–H bond in the catechol, making it more polar and less strong, thereby facilitating the electrooxidation process. These findings are confirmed by IR spectroscopy: for the corresponding thioether, the O–H stretching vibrations are observed as two bands at 3522 and 3282 cm^−1^, whereas for sulfoxide **5a**, they are observed at 3509 and 3276 cm^−1^. For sulfoxides containing primary/secondary aliphatic substituents (**1a**, **3a**, **4a**, **6a**) or a 1-naphthyl moiety (**7a**), the second redox stage proceeds as an irreversible process in the potential range of 1.82–1.91 V. The value of *E*_p_^ox2^ for the above-mentioned compounds is shifted anodically by 0.26–0.31 V relative to the corresponding thioethers, reflecting an increase in the Gibbs free energy (Δ*G*) of the electrooxidation process by 12–15 kcal/mol. Redox transformations at the sulfur atom occur in either one or two stages, depending on the degree of branching of the hydrocarbon substituent. A possible mechanism for the anodic transformations of sulfoxides is the two-electron oxidation of the substituted *o*-benzoquinone formed in the first stage to a dication (Scheme 2, path a).

**Figure 3:**
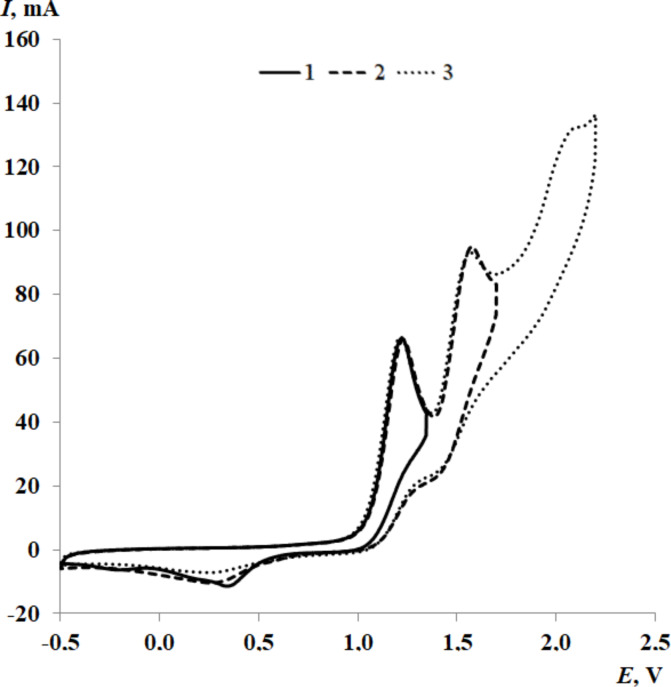
CV curve of **5a** at the potential range from −0.50 to 1.40 V (curve 1); from −0.50 to 1.70 V (curve 2); from −0.50 to 2.20 V (curve 3) (CH_3_CN, GC electrode, Ag/AgCl/KCl(sat.), 0.15 M *n*-Bu_4_NClO_4_, *c* = 3 mmol·L^−1^).

Further transformations of the sulfur-centered intermediate can lead to the corresponding sulfone via a disproportionation reaction or in the presence of traces of water [49]. In the case of compounds **2a** and **5a**, which bear a tertiary hydrocarbon substituent at the sulfinyl group (*tert*-butyl and adamantyl, respectively), the second redox stage splits into two steps. The first yields a relatively stable cation radical (*I_c_*/*I_a_ =* 0.45–0.50) (Scheme 2, path b), while the subsequent redox transition occurs at higher potentials (2.03–2.06 V) (Figure 3). In the case of catechols **7** and **7a**, the second oxidation peak appears in a potential range where oxidation of the naphthyl fragment also takes place, resulting in an increase in the current of this anodic peak (Figure S32 in Supporting Information File 1).

**Figure 4:**
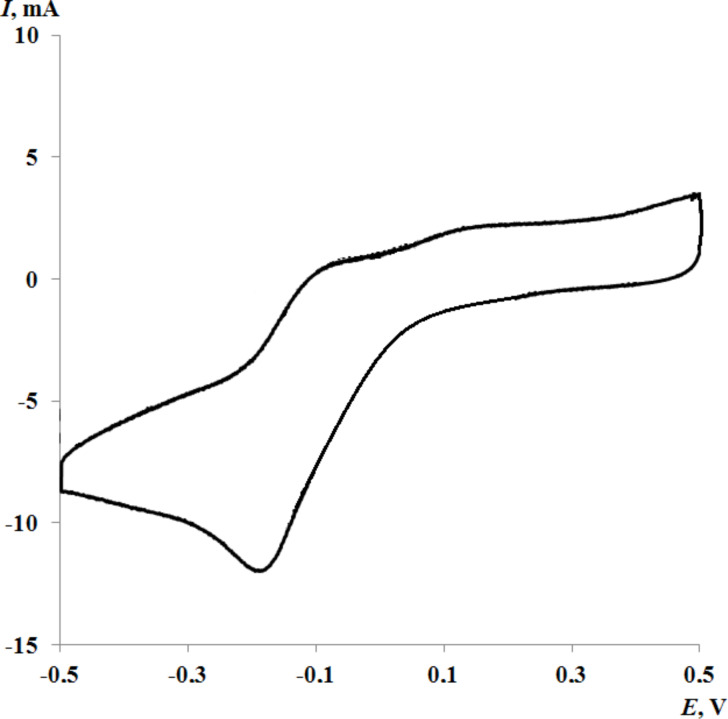
CV curves of electrolysis products of **7a** at the potential ranges from 0.5 to −0.4 V (CH_3_CN, GC electrode, Ag/AgCl/KCl(sat.), 0.15 M *n*-Bu_4_NClO_4_, *c* = 3 mmol·L^−1^, *t* = 1 h).

To confirm the participation of the catechol group in the first redox stage, the microelectrolysis of **5a**–**7a** was performed in MeCN at controlled potentials of 1.25 V (for **5a**) or 1.35 V (for **6a**, **7a**) for 1 h, with a charge consumption of 0.46–0.57 F/mol. After electrolysis, the cyclic voltammograms of these compounds showed a decrease in the first oxidation peak current, with conversions of 22%, 66%, and 48% for **5a**, **6a**, and **7a**, respectively. Coulometric data indicated that the amount of electricity consumed during electrolysis corresponds to the transfer of two electrons. In the cathodic region, a one-electron quasi-reversible peak (*E*_pc_) is displayed (*E*_pc_ = −0.21 V (for **5a**), −0.18 V (for **6a**), −0.16 V (for **7a**) (Figure 4).

**Scheme 3:**
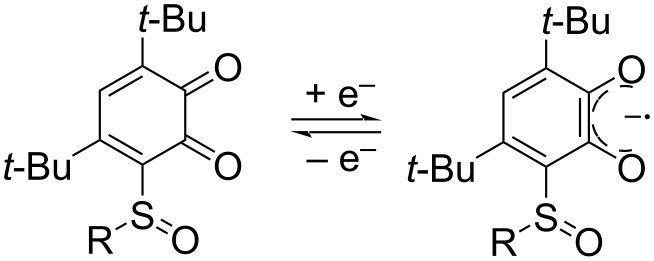
Proposed mechanism of the reduction of electrogenerated *o*-benzoquinone.

This process corresponds to the reduction of electrogenerated *o*-benzoquinone containing S=O group to *o*-benzosemiquinone (Scheme 3).

The obtained *E*_pc_ values are significantly shifted towards positive potentials compared to the reduction potentials of electrogenerated S-functionalized *o*-benzoquinones (−0.41 to −0.37) V [23]) and halogen-substituted 3,6-di-*tert*-butyl-4-chloro-*o*-benzoquinone (−0.44 V [50]), due to the electron-withdrawing effect of the sulfinyl group. This allows the electrogenerated *o*-benzoquinones to be considered as fairly strong electron acceptors. During electrolysis, the color of the solution changes to pale yellow. This is confirmed by the appearance of an absorption maximum in the UV–visible spectrum at λ_max_ = 402 nm (Figure S33 in Supporting Information File 1). This absorption band corresponds to a π→π* transition and is close to the λ_max_ value characteristic of sterically hindered *o*-benzoquinones. The obtained data differ from the spectral pattern characteristic of *o*-benzoquinone thioethers (λ_max_ = 490–505 nm) [23,51–53] and indicate the absence of intramolecular charge transfer between the thioether group and the *o*-benzoquinone fragment. Based on electrochemical data, it can be assumed that the most pronounced antioxidant activity can be exhibited by catechol thioethers than the corresponding sulfoxides.

### The radical scavenging activity of catechol thioethers vs sulfoxides

The radical scavenging properties and antioxidant activity of new catechol sulfoxides **1a**–**7a** were investigated in comparison with thioethers **1**–**7**, 3,5-DTBC and standard antioxidant Trolox using the 2,2′-diphenyl-1-picrylhydrazyl (DPPH) radical and the ABTS^·+^ radical cation. The presence of a catechol fragment acting as a primary antioxidant and thioether or sulfoxide groups serving as a secondary antioxidant center endows these compounds with the ability to neutralize different types of radicals. A comparative evaluation of the antioxidant activity of catechol thioethers **1**–**7**, sulfoxides **1a**–**7a** and Trolox was performed in a reaction with DPPH in acetonitrile at 298 K (Table 2). Addition of the target catechols to a DPPH radical solution leads to a decrease in absorbance at the 517 nm maximum. The antiradical activities of CatH_2_-S-R and CatH_2_-SO-R were evaluated through the determination of IC_50_ and the time to reach the steady-state equilibrium (TEC_50_) values, together with the antiradical efficiency (AE). This complex parameter captures both the hydrogen-atom-donating ability of a compound and the kinetics of its reaction with the DPPH radical.

**Table 2:** Radical scavenging activity characteristics of catechols thioethers **1**–**7**, sulfoxides **1a**–**7a,** 3,5-DTBC and Trolox in DPPH (CH_3_CN, 298 K), ABTS^•+^ assay expressed as IC_50_.

Compound	DPPH			ABTS^∙+^

	IC_50_ (DPPH), µM	TEC_50_, min	AE·10^3^	IC_50_ (ABTS^∙+^), µM

**1**	16.3 ± 0.8	25	2.45	7.2 ± 0.3
**2**	12.5 ± 0.3	40	2.00	8.7 ± 0.5
**3**	14.5 ± 0.6^a^	40^a^	1.72	12.9 ± 0.8
**4**	11.1 ± 0.9^a^	35^a^	2.57	10.1 ± 0.4
**5**	11.7 ± 0.4^b^	40^b^	2.14	11.5 ± 0.8
**6**	11.5 ± 0.4^a^	32^a^	2.72	10.4 ± 0.5
**7**	25.3 ± 1.3	20	1.98	9.7 ± 0.7
**1a**	10.8 ± 0.5	26	3.56	9.3 ± 0.3
**2a**	14.1 ± 1.3	70	1.01	8.4 ± 0.2
**3a**	17.9 ± 1.0	48	1.16	12.9 ± 0.6
**4a**	19.8 ± 1.5	32	1.58	10.2 ± 0.7
**5a**	20.4 ± 1.4	45	1.09	17.5 ± 1.4
**6a**	18.6 ± 1.0	30	1.79	12.2 ± 0.6
**7a**	22.3 ± 2.0	35	1.28	16.1 ± 1.1
3,5-DTBC	13.1 ± 1.3^a^	60^a^	1.33^a^	12.9 ± 0.3
Trolox [41]	12.0 ± 0.5	10.3	8.09	16.0 ± 1.0

^a^Data were obtained from [23]; ^b^data were obtained from [42].

Among catechol thioethers **1**, **2**, and **7**, compound **2**, bearing a *tert*-butyl substituent on the sulfur atom, exhibited the highest activity based on IC_50_ (12.5 ± 0.3 μM). This value is consistent with previously reported IC_50_ values for thioethers **3**–**6** (11.1–14.5 μM) and for 4,6-di-*tert*-butyl-3-(butylsulfanyl)benzene-1,2-diol (12.0 ± 0.5 μM) [23]. In the series of sulfoxides **1a**–**7a**, the IC_50_ values ranged from 10.8 to 22.3 μM, indicating an overall decrease in radical-scavenging activity upon introduction of the sulfinyl group. An exception is catechol sulfoxide **1a** with an isopropyl substituent, which showed the lowest IC_50_ value (10.8 ± 0.5 μM), indicating enhanced antiradical activity relative to its thioether precursor **1** (16.3 ± 0.8 μM) and to 3,5-DTBC (13.1 ± 1.3 μM). The number of converted DPPH molecules per one molecule (*n*_DPPH_) for **1a** is more than two. The high antiradical activity of sulfoxide **1a** may be attributed to its structure, specifically to the smaller size of the alkyl substituent at the S=O group relative to the other compounds. Despite the branched nature of the iPr group, the sulfinyl moiety may serve more pronouncedly as a secondary antioxidant center, as also indicated by the smaller TEC_50_ value. For all other sulfoxides, the *n*_DPPH_ value was lower than that of the corresponding thioethers, a trend that is indirectly supported by the oxidation potential data. As the number of carbon atoms in the aliphatic hydrocarbon substituents of sulfoxides increases, their activity in the DPPH test diminishes. Compounds **7** and **7a**, which contain a naphthyl substituent, exhibit the highest IC_50_ values among the studied catechols, indicating low radical scavenging activity. For sulfoxides **1a**–**7a**, the TEC_50_ ranged from 30 to 70 min, which was longer than that for thioethers **1**–**7** (20–40 min). The antiradical efficiency (AE = 1/(IC_50_·TEC_50_)) values point out a moderate neutralizing capacity for catechol sulfoxide **1a** (AE = 3.56) and its corresponding thioether **1** (AE = 2.45), both containing an isopropyl substituent. However, these AE values exceeded that of 3,5-DTBC, indicating enhanced neutralizing activity of the sulfur-containing derivatives.

The ABTS assay is one of the most widely used methods for assessing the antioxidant capacity of compounds; it is based on electron transfer from a potential antioxidant to the ABTS cation radical (ABTS^∙+^) as an acceptor [54]. The IC_50_(ABTS^∙+^) values for synthesized catechol sulfoxides ranged from 8.4 to 17.5 µM, which is slightly higher than those measured for thioethers (7.2–12.5 µM). The IC_50_ values obtained in the ABTS-test were lower than those determined in the DPPH assay, which is due to the different reactivity of the two radicals. The most pronounced antioxidant activity (7.2–9.3 µM) was exhibited by compounds **1**, **1a**, **2**, and **2a**, bearing an isopropyl or *tert*-butyl substituent at the thioether/sulfoxide group. These values surpassed that of 3,5-DTBC (12.9 ± 0.3 μM). For the catechols **3**/**3a** (with octyl group) and **4**/**4a** (cyclopentyl group), the IC_50_(ABTS^∙+^) values are comparable regardless of the oxidation state of the sulfur atom. Exceptions were the pairs containing adamantyl (**5**/**5a**) and 1-naphthyl (**7**/**7a**) substituents, where an increase in the IC_50_(ABTS^∙+^) was observed upon going from the thioethers to the sulfoxides. Furthermore, sulfoxides **5a** and **7a** showed a decreased efficiency in the ABTS assay relative to 3,5-DTBC. Overall, all studied catechols possessed antioxidant activity comparable to that of Trolox or exceeding it by 19–55%.

## Conclusion

In summary, we have developed a synthetic approach to a new family of unsymmetrical redox-active catechol sulfoxides bearing lipophilic hydrocarbon substituents, obtained in good yields (42–89%) via chemoselective oxidation of the corresponding thioethers. Single-crystal X-ray diffraction analysis of catechol sulfoxides containing isopropyl, cyclopentyl, adamantyl, benzyl, and 1-naphthyl groups, combined with NMR and IR spectroscopy, revealed a consistent tendency of these sulfoxides to form intra- and intermolecular O–H···O=S hydrogen bonds both in the solid state and in solution, highlighting the structural role of the sulfinyl group.

Cyclic voltammetry demonstrated that the oxidation potential of the catechol fragment is systematically modulated by the oxidation state of the sulfur atom. The introduction of the S=O group shifts the first oxidation process (catechol/*o*-benzoquinone) anodically by up to 0.09 V relative to the parent thioethers, reflecting the electron-withdrawing nature of the sulfinyl moiety. The second stage, attributed to transformation of the sulfoxide group, is observed at 1.60–2.05 V, whereas the oxidation of the thioether group appears at lower potentials (1.52–1.66 V). This electronic tuning is directly correlated with the radical-scavenging performance: in both DPPH and ABTS assays. The target sulfoxides exhibited IC_50_ values ranging from 10.8 to 22.3 μM (DPPH) and from 8.4 to 17.5 μM (ABTS^+•^), with the lowest values observed for compounds bearing isopropyl and *tert*-butyl substituents. Notably, sulfoxide **1a** (isopropyl) showed enhanced antiradical activity compared to its thioether precursor, while for most other derivatives the activity decreased upon oxidation of sulfur. The combination of a primary antioxidant catechol unit and a secondary sulfur-based redox center enables a dual mechanism of radical neutralization, the efficiency of which can be tuned by the nature of the hydrocarbon substituent and the oxidation state of sulfur.

The structural, electrochemical, and antiradical data presented herein establish a coherent framework for understanding the behavior of S-functionalized catechols. The ability to control the hydrogen-bonding propensity, redox potentials, and antioxidant activity by varying the substituent at sulfur and its oxidation level opens promising avenues for the design of biomimetic antioxidants, stimuli-responsive materials, and functional ligands for metal complexes with tailored electronic properties.

## Supporting Information

File 1 Experimental procedures and characterization data.

File 2 Crystallographic information files.

## Data Availability

All data that supports the findings of this study is available in the published article and/or the supporting information of this article.
