# Supplementary material for: Unsymmetrical sulfoxides with sterically hindered catechol fragment: synthesis, structure, electrochemical properties, and antiradical activity
Source: Beilstein J Org Chem. 2026 Jun 1;22:828–37. doi: 10.3762/bjoc.22.65 (PMC13245472; doi:10.3762/bjoc.22.65)
Supplement: File 1 — Experimental procedures and characterization data. [file Beilstein_J_Org_Chem-22-828-s001.pdf]

BEILSTEIN JOURNAL OF ORGANIC CHEMISTRY

Supporting Information

for

Unsymmetrical sulfoxides with sterically hindered catechol fragment: synthesis, structure, electrochemical properties, and antiradical activity

Daria A. Burmistrova, Vasiliy A. Fokin, Oleg P. Demidov, Mikhail A. Kiskin, Maxim V. Arsenyev, Andrey I. Poddel’sky, Nadezhda T. Berberova and Ivan V. Smolyaninov

Beilstein J. Org. Chem. **2026,** *22,* 828–837. doi:10.3762/bjoc.22.65

Experimental procedures and characterization data

License and Terms: This is a supporting information file under the terms of the Creative Commons Attribution License (https://creativecommons.org/licenses/by/4.0). Please note that the reuse, redistribution and reproduction in particular requires that the author(s) and source are credited and that individual graphics may be subject to special legal provisions.
The license is subject to the *Beilstein Journal of Organic Chemistry* terms and conditions: (https://www.beilstein-journals.org/bjoc/terms)

Content

S1. Experimental procedures S4

S1.1. General S4

S1.2. Instrumentation S4

S1.3. Synthesis and characterization S5

S1.4. X-ray structures S13

Table S1. Experimental details and crystallographic data for compounds **1a**, **4a**–**7a** S14

Table S2: Selected bond lengths (Å) and bond angles (°) of **1a**, **4a**–**7a** S16

Table S2. C-H···A interactions in crystals of **1a**, **4a**–**7a** S17

Table S3. C–X···π interactions in **1a**, **4a**–**7a** S20

S1.5. Antioxidant activity assay S20

S1.5.1. DPPH radical scavenging activity assay S20

S1.5.2. ABTS assay S20

S2. NMR-spectra S21

Figure S1. The ^{1}H NMR spectrum of 4,6-di-*tert*-butyl-3-(isopropylthio)benzene-1,2-diol (**1**) in $CDCl_3$ S21

Figure S2. The $^{13}C\{^{1}H\}$ NMR spectrum of 4,6-di-*tert*-butyl-3-(isopropylthio)benzene-1,2-diol in $CDCl_3$ (**1**) S21

Figure S3. The ^{1}H NMR spectrum of 4,6-di-*tert*-butyl-3-(isopropylsulfinyl)benzene-1,2-diol (**1a**) in $CDCl_3$ S22

Figure S4. The $^{13}C\{^{1}H\}$ NMR spectrum of of 4,6-di-*tert*-butyl-3-(isopropylsulfinyl)benzene-1,2-diol (**1a**) in $CDCl_3$ S22

Figure S5. The ^{1}H NMR spectrum of 4,6-di-*tert*-butyl-3-(*tert*-butylthio)benzene-1,2-diol (**2**) in $CDCl_3$ S23

Figure S6. The $^{13}C\{^{1}H\}$ NMR spectrum of 4,6-di-*tert*-butyl-3-(*tert*-butylthio)benzene-1,2-diol (**2**) in $CDCl_3$ S23
Figure S7. The ^{1}H NMR spectrum of 4,6-di-*tert*-butyl-3-(*tert*-butylsulfinyl)benzene-1,2-diol (**2a**) in $CDCl_3$ S24
Figure S8. The $^{13}C\{^{1}H\}$ NMR spectrum of 4,6-di-*tert*-butyl-3-(*tert*-butylsulfinyl)benzene-1,2-diol (**2a**) in $CDCl_3$ S24
Figure S9. The ^{1}H NMR spectrum of 4,6-di-*tert*-butyl-3-(octylsulfinyl)benzene-1,2-diol (**3a**) in $CDCl_3$ S25
Figure S10. The $^{13}C\{^{1}H\}$ NMR spectrum of 4,6-di-*tert*-butyl-3-(octylsulfinyl)benzene-1,2-diol (**3a**) in $CDCl_3$ S25
Figure S11. The ^{1}H NMR spectrum of 4,6-di-*tert*-butyl-3-(cyclopentylsulfinyl)benzene-1,2-diol (**4a**) in $CDCl_3$ S26
Figure S12. The $^{13}C\{^{1}H\}$ NMR spectrum of 4,6-di-*tert*-butyl-3-(cyclopentylsulfinyl)-benzene-1,2-diol (**4a**) in $CDCl_3$ S26
Figure S13. The ^{1}H NMR spectrum of 3-(((3s,5s,7s)-adamantan-1-yl)sulfinyl)-4,6-di-*tert*-butylbenzene-1,2-diol (**5a**) in $CDCl_3$ S27
Figure S14. The $^{13}C\{^{1}H\}$ NMR spectrum of 3-(((3s,5s,7s)-adamantan-1-yl)sulfinyl)-4,6-di-*tert*-butylbenzene-1,2-diol (**5a**) in $CDCl_3$ S27
Figure S15. The ^{1}H NMR spectrum of 3-(benzylsulfinyl)-4,6-di-*tert*-butylbenzene-1,2-diol in $CDCl_3$ (**6a**) S28
Figure S16. The $^{13}C\{^{1}H\}$ NMR spectrum of 3-(benzylsulfinyl)-4,6-di-*tert*-butylbenzene-1,2-diol in $CDCl_3$ (**6a**) S28
Figure S17. The ^{1}H NMR spectrum of 4,6-di-*tert*-butyl-3-(naphthalen-1-ylthio)benzene-1,2-diol in $CDCl_3$ (**7**) S29
Figure S18. The $^{13}C\{^{1}H\}$ NMR spectrum of 4,6-di-*tert*-butyl-3-(naphthalen-1-ylthio)benzene-1,2-diol in DMSO-d_6 (**7**) S29
Figure S19. The ^{1}H NMR spectrum of 4,6-di-*tert*-butyl-3-(naphthalen-1-ylsulfinyl)benzene-1,2-diol in $CDCl_3$ (**7a**) S30
Figure S20. The $^{13}C\{^{1}H\}$ NMR spectrum of 4,6-di-*tert*-butyl-3-(naphthalen-1-ylsulfinyl)benzene-1,2-diol (**7a**) S30
S3. HRMS-Spectra S31
Figure S21. The HRMS spectrum of **1a** S31
Figure S22. The HRMS spectrum of **2a** S31
Figure S23. The HRMS spectrum of **3a** S32
Figure S24. The HRMS spectrum of **6a** S32

Figure S25. The HRMS spectrum of **7** S33
Figure S26. The HRMS spectrum of **7a** S33
S4. Electrochemical data S34
Figure S27. The CV curve of **2** S34
Figure S28. The CV curve of **2a** S34
Figure S29. The CV curve of **3a** S35
Figure S30. The CV curve of **4a** S35
Figure S31. The CV curve of **6a** S36
Figure S32. The CV curve of **7** and **7a** S36
S5. UV–vis spectroscopy S37
Figure S33. UV–vis spectra of the products of electrolysis of **5a** S37
S6. References S37

S1. Experimental procedures

S1.1. General

All starting reagents were commercially available: 3,5-di-*tert*-butyl-*o*-benzoquinone (99%, Alfa Aesar), 2-propanethiol (98%, Aldrich), 2-methyl-2-propanethiol (99%, Aldrich), 1-naphthalenethiol (99%, Aldrich), tetra-*n*-butylammonium perchlorate (>99%, Alfa Aesar), hydrogen peroxide (30%, Aldrich), acetic acid (99.5%, AppliChem), 2,2'-azino-bis(3-ethylbenzothiazoline-6-sulfonic acid) (≥98%, TCI), potassium persulfate (99%, Sigma Aldrich), 2,2-diphenyl-1-picrylhydrazyl (Aldrich). The catechol thioethers **3–6** were synthesized by the known methods [1,2]: 4,6-di-*tert*-butyl-3-(octylthio)benzene-1,2-diol (**3**) [1], 4,6-di-*tert*-butyl-3-(cyclopentylthio)benzene-1,2-diol (**4**) [1], 3-((3s,5s,7s)-adamantan-1-ylthio)-4,6-di-*tert*-butylbenzene-1,2-diol (**5**) [2], 4,6-di-*tert*-butyl-3-(benzylthio)benzene-1,2-diol (**6**) [1]. Standard procedures have been used to purify solvents [3].

S1.2. Instrumentation

An FSM-1201 FT-IR spectrometer was used to record IR spectra in KBr pellets. The ^{1}H and ^{13}C{^{1}H} NMR spectra were measured in $CDCl_3$ or DMSO on Bruker Avance HD 400 spectrometer with a frequency of 400 MHz for ^{1}H and 100 MHz for ^{13}C{1H} NMR spectra or Bruker Avance DPX-300 with a frequency of 300 MHz for ^{1}H and 75 MHz for ^{13}C{1H} NMR spectra. The chemical shift values are given in ppm with reference to the solvent, and the coupling constants (*J*) are given in Hz. The Euro EA 3000 (C,H,N) elemental analyzer was used to determine the elemental composition of the synthesized substances. High-resolution mass spectra (HRMS) were recorded on a mass spectrometer Bruker UHR-TOF Maxis™ (ESI). The UV–vis spectra were recorded with a SF-104 spectrophotometer (Akvilon, Podol'sk, Russia) in a range of 300–1000 nm. To determine the electrochemical potentials of catechol thioethers **1–7** and sulfoxides **1a–7a**, a technique of cyclic voltammetry (CV) was used. The measurements occurred in

a three-electrode cell on a VersaSTAT3 (Princeton Applied Research) potentiostate in CH_3CN under argon. A stationary glassy carbon (GC) electrode with a diameter of 2 mm was the working electrode and a platinum plate (S = 18 mm^2) served as the auxiliary electrode. The potentials of the complexes were measured versus the reference electrode (Ag/AgCl/KCl) with a waterproof membrane. The number of electrons transferred during the electrode process was estimated relative to ferrocene as the standard. The concentration of compounds was 3 mmol in CH_3CN containing 0.1 M n-Bu_4NClO_4 as supporting electrolyte, with scan rates of 0.2 $V\cdot s^{-1}$.

Microelectrolysis of catechol sulfoxides **5a**–**7a** was performed on a VersaSTAT3 potentiostate at stationary platinum electrodes (S = 18 mm^2) in an undivided three-electrode cell (2 mL) under anaerobic conditions. Catechol was added to an electrochemical cell containing a solution of the supporting electrolyte (0.1 M Bu_4NClO_4) in acetonitrile. Electrolysis was performed in the potentiostatic mode at a potential of 1.25–1.35 V.

S1.3. Synthesis and characterization

New catechol thioethers **1**, **2**, and **7** were obtained from 3,5-di-*tert*-butyl-*o*-benzoquinone (1.0 g, 4.5 mmol) and corresponding thiol (5.4 mmol) in ethanol (10 mL) under an inert atmosphere (argon). The synthesis was carried out for 4–5 hours until the reaction mixture became completely colorless. The solvent was evaporated under reduced pressure. The formed precipitate was recrystallized from acetonitrile. Compounds **3**–**6** were previously prepared [1,2]. Catechol sulfoxides **1a**–**7a** were synthesized by oxidation of the corresponding thioether **1**–**7** (1.6 mmol) in the presence of 30% H_2O_2 (1.0 mmol) and AcOH (2:1 v/v) in acetone (3 mL) with stirring for 4–5 h at 50 °C. After the reaction, 10 mL of water was added to the solution; the resulting precipitate was filtered off, dried in a vacuum, and recrystallized from acetonitrile, hexane (for **4a**) or chloroform (for **7a**).

4,6-Di-*tert*-butyl-3-(isopropylthio)benzene-1,2-diol (1)

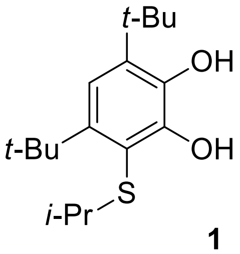

1

Yield 0.840 g (63%). White crystalls with m.p. 88–90ºC.

IR (KBr, v/cm^{-1}): 3480, 3390, 2997, 2959, 2907, 2868, 1700, 1685, 1654, 1606, 1559, 1542, 1522, 1520, 1506, 1483, 1458, 1446, 1397, 1364, 1349, 1293, 1263, 1236, 1201, 1179, 1155, 1044, 1025, 964, 865.

^{1}H NMR (400 MHz, $CDCl_3$, δ, ppm): 1.27 (d, ^{3}J(H,H) = 6.7 Hz, 6H, iPr), 1.40 (s, 9H, tBu), 1.48 (s, 9H, tBu), 3.13 (hept, ^{3}J(H,H) = 6.7 Hz, 1H, CH), 5.55 (s, 1H, OH), 6.92 (s, 1H, arom. C_6H_1), 7.28 (s, 1H, OH).

^{13}C{^{1}H} NMR (100 MHz, $CDCl_3$, δ, ppm): 23.4, 29.4, 31.9, 35.0, 35.3, 37.1, 41.9, 114.7, 116.2, 136.1, 140.5, 143.8, 145.9.

HR-MS: Found m/z: 295.1744 [M-H]$^{+}$. $C_{17}H_{27}O_2S$. Calcd. m/z: 295.1737.

4,6-Di-*tert*-butyl-3-(isopropylsulfinyl)benzene-1,2-diol (1a)

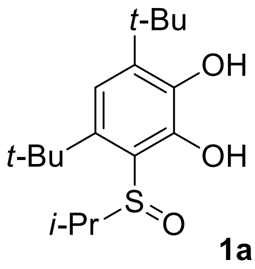

1a

Yield 0.290 g (58%). White crystalls with m.p. 140–142ºC.

IR (KBr, v/cm^{-1}): 3418, 3368, 2991, 2966, 2906, 2872, 1701, 1654, 1604, 1570, 1558, 1540, 1508, 1485, 1467, 1403, 1367, 1345, 1292, 1270, 1248, 1218, 1207, 1169, 1162, 1062, 1026, 977, 960, 946 (S=O), 865.

^{1}H NMR (400 MHz, $CDCl_3$, δ, ppm): 1.25 (d, ^{3}J(H,H) = 7.0 Hz, 3H, iPr), 1.40 (br. s, 18H, 2 tBu), 1.50 (d, ^{3}J(H,H) = 6.6 Hz, 3H, iPr), 3.53-3.66 (m, 1H, CH), 6.08 (s, 1H, OH), 6.87 (s, 1H, arom. C_6H_1), 11.47 (s, 1H, OH).

$^{13}C\{^{1}H\}$ NMR (100 MHz, $CDCl_3$, δ, ppm): 16.3, 19.0, 29.2, 33.3, 35.3, 36.7, 54.4, 116.0, 117.1, 137.9, 139.1, 143.1, 148.9.

HR-MS: Found m/z: 335.1656 $[M+Na]^+$. $C_{17}H_{28}NaO_3S$. Calcd. m/z: 335.1651.

4,6-Di-*tert*-butyl-3-(*tert*-butylthio)benzene-1,2-diol (2)

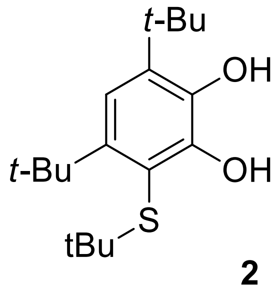

2

Yield 0.711 g (51%). White crystalls with m.p. 94–96ºC.

IR (KBr, ν/cm^{-1}): 3506, 3257, 3003, 2959, 2910, 2868, 1730, 1608, 1566, 1487, 1475, 1459, 1396, 1367, 1309, 1288, 1261, 1240, 1170, 1151, 1026, 962, 935, 866.

^{1}H NMR (400 MHz, $CDCl_3$, δ, ppm): 1.36 (s, 9H, tBu), 1.42 (s, 9H, tBu), 1.48 (s, 9H, tBu), 5.55 (s, 1H, OH), 6.93 (s, 1H, arom. C_6H_1), 7.11 (s, 1H, OH).

$^{13}C\{^{1}H\}$ NMR (100 MHz, $CDCl_3$, δ, ppm): 29.5, 32.5, 35.2, 37.2, 49.9, 114.7, 116.3, 136.1, 140.4, 144.2, 146.1.

HR-MS: Found m/z: 309.1895 $[M-H]^+$. $C_{18}H_{29}O_2S$. Calcd. m/z: 309.1894.

4,6-Di-*tert*-butyl-3-(*tert*-butylsulfinyl)benzene-1,2-diol (2a)

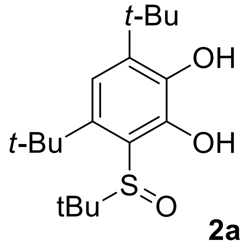

2a

Yield 0.313 g (60%). White crystalls with m.p. 139–141ºC.

IR (KBr, ν/cm^{-1}): 3465, 2990, 2965, 2912, 2874, 1733, 1600, 1573, 1545, 1484, 1460, 1413, 1394, 1369, 1355, 1288, 1266, 1235, 1160, 1093, 1028, 970, 947 (S=O), 869.

^{1}H NMR (400 MHz, $CDCl_3$, δ, ppm): 1.38 (s, 9H, tBu), 1.39 (s, 9H, tBu), 1.42 (s, 9H, tBu), 6.04 (s, 1H, OH), 6.88 (s, 1H, arom. C_6H_1), 11.74 (s, 1H, OH).

^{13}C{^{1}H} NMR (100 MHz, $CDCl_3$, δ, ppm): 26.4, 29.3, 34.1, 35.3, 37.2, 62.2, 114.8, 116.5, 138.0, 140.1, 142.7, 149.6.

HR-MS: Found m/z: 349.1812 [M+Na]$^{+}$. $C_{18}H_{30}NaO_3S$. Calcd. m/z: 349.1808.

4,6-Di-*tert*-butyl-3-(octylsulfinyl)benzene-1,2-diol (3a)

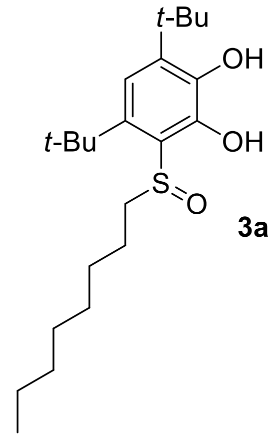

3a

Yield 0.300 g (49%). White powder with m.p. 99–101ºC.

IR (KBr, ν/cm^{-1}): 3260, 2959, 2926, 2858, 1653, 1601, 1567, 1540, 1508, 1482, 1470, 1458, 1400, 1364, 1341, 1291, 1268, 1221, 1205, 1168, 1025, 966, 867.

^{1}H NMR (400 MHz, $CDCl_3$, δ, ppm): 0.88 (t, *J* = 6.1 Hz, 3H, CH_3), 1.22-1.35 (m, 8H, 4 CH_2), 1.38 (s, 9 H, tBu), 1.39 (s, 9 H, tBu), 1.43-1.54 (m, 2H, CH_2), 1.88-1.94 (m, 2H, CH_2), 2.84-2.94 (m, 1H, CH_2S), 3.54-3.62 (m, 1H, CH_2S), 6.10 (s, 1H, OH), 6.84 (s, 1H, arom. C_6H_1), 11.33 (s, 1H, OH).

$^{13}C\{^1H\}$ NMR (100 MHz, $CDCl_3$, δ, ppm): 14.2, 22.7, 24.3, 28.6, 29.2, 29.2, 29.3, 31.9, 32.6, 35.3, 36.3, 52.4, 115.5, 119.6, 137.8, 137.9, 143.4, 148.5.

HR-MS: Found m/z: 405.2440 $[M+Na]^+$. $C_{22}H_{38}NaO_3S$. Calcd. m/z: 405.2434.

4,6-Di-*tert*-butyl-3-(cyclopentylsulfinyl)benzene-1,2-diol (4a)

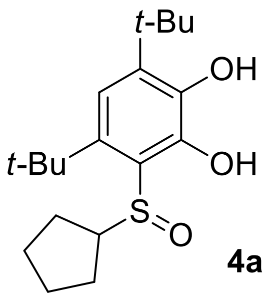

4a

Yield 0.297 g (55%). White powder with m.p. 124–126ºC.

IR (KBr, ν/cm^{-1}): 3259, 2958, 2914, 2870, 1653, 1602, 1570, 1561, 1485, 1472, 1456, 1403, 1367, 1349, 1291, 1267, 1227, 1170, 1025, 973, 945 (S=O), 864.

^{1}H NMR (400 MHz, $CDCl_3$, δ, ppm): 1.39 (s, 9 H, tBu), 1.40 (s, 9 H, tBu), 1.60-1.94 (m, 4H, CH_2), 2.00-2.09 (m, 2H, CH_2), 2.25-2.34 (m, 2H, CH_2), 3.80 (p, J = 7.7 Hz, 1H, CH, cyclopentyl), 6.09 (s, 1H, OH), 6.85 (s, 1H, arom. C_6H_1), 11.37 (br.s., 1H, OH).

$^{13}C\{^1H\}$ NMR (100 MHz, $CDCl_3$, δ, ppm): 25.2, 25.4, 27.1, 29.0, 29.2, 33.2, 35.3, 36.6, 63.5, 115.9, 118.5, 137.8, 138.5, 143.1, 148.8.

Calcd. for $C_{19}H_{30}O_3S$ (%): C, 67.42; H, 8.93 Found (%): C, 67.21; H, 9.18.

3-(((3s,5s,7s)-Adamantan-1-yl)sulfinyl)-4,6-di-*tert*-butylbenzene-1,2-diol (5a)

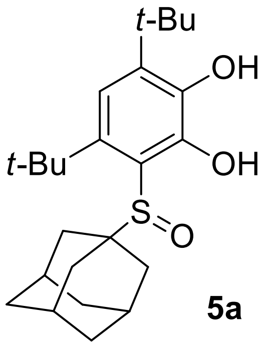

5a

Yield 0.272 g (42%). White powder with m.p. 162–164ºC.

IR (KBr, ν/cm^{-1}): 3509, 3276, 2997, 2962, 2906, 2853, 1602, 1566, 1484, 1472, 1451, 1401, 1366, 1345, 1298, 1269, 1247, 1223, 1170, 1104, 1036, 972, 962, 951 (S=O), 935, 865, 816, 788.

^{1}H NMR (300 MHz, $CDCl_3$, δ, ppm): 1.40 (s, 9H, tBu), 1.41 (s, 9H, tBu), 1.65-1.71 (m, 6H, $3CH_2$), 1.90-2.08 (m, 6H, S-$C(CH_2)_3$), 2.12-2.20 (m, 3H, 3CH), 6.04 (s, 1H, OH), 6.88 (s, 1H, C_6H_1 arom.), 11.85 (s, 1H, OH).

$^{13}C\{^{1}H\}$ NMR (75 MHz, $CDCl_3$, δ, ppm): 29.3, 29.9, 34.2, 35.3, 36.1, 37.2, 37.9, 64.4, 113.4, 116.6, 137.8, 140.3, 142.6, 145.0.

Calcd. for $C_{24}H_{36}O_3S$ (%): C, 71.24; H, 8.97. Found (%): C, 71.05; H, 9.45.

3-(Benzylsulfinyl)-4,6-di-*tert*-butylbenzene-1,2-diol (6a)

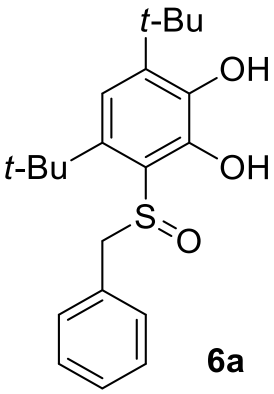

6a

Yield 0.422 g (73%). White crystalls with m.p. 151–152ºC.

IR (KBr, ν/cm^{-1}): 3340, 3091, 3067, 2959, 2909, 2870, 1653, 1604, 1559, 1541, 1521, 1507, 1495, 1483, 1473, 1456, 1437, 1401, 1366, 1347, 1292, 1266, 1228, 1205, 1171, 1071, 1026, 975, 949 (S=O), 910, 878, 863, 787.

^{1}H NMR (400 MHz, $CDCl_3$, δ, ppm): 1.41 (s., 9H, tBu), 1.43 (s., 9H, tBu), 4.27 (d, $^2J(H,H)$ = 13.1 Hz, 1H, CH_2), 4.66 (d, $^2J(H,H)$ = 13.1 Hz, 1H, CH_2), 6.17 (s., 1H, OH), 6.89 (s., 1H, arom. C_6H_1), 7.32 – 7.45 (m., 5H, Ph), 11.38 (s., 1H, OH).

$^{13}C\{^1H\}$ NMR (100 MHz, $CDCl_3$, δ, ppm): 29.2, 32.7, 35.4, 36.4, 58.4, 115.7, 119.3, 128.9, 129.2, 130.4, 131.1, 138.2, 138.3, 143.4, 148.4.

HR-MS: Found m/z: 383.1652 $[M+Na]^+$. $C_{21}H_{28}NaO_3S$. Calcd. m/z: 383.1651.

4,6-Di-*tert*-butyl-3-(naphthalen-1-ylthio)benzene-1,2-diol (7)

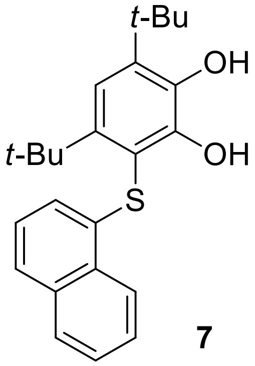

7

Yield 1.045 g (61%). White powder with m.p. 118–120ºC.

IR (KBr, ν/cm^{-1}): 3537, 3403, 3081, 3055, 2998, 2989, 2961, 2909, 2867, 1733, 1700, 1685, 1653, 1591, 1564, 1446, 1365, 1350, 1289, 1259, 1236, 1171, 1143, 1060, 1024, 965, 870.

1H NMR (400 MHz, $CDCl_3$, δ, ppm): 1.44 (s., 9H, tBu), 1.48 (s., 9H, tBu), 5.62 (s., 1H, OH), 6.60 (d., J = 7.4 Hz, 1H, arom. $C_{10}H_7$), 6.75 (s., 1H, arom. C_6H_1), 7.10 (s, 1H, OH), 7.26 (d., 1H, arom. $C_{10}H_7$), 7.56 (t., J = 7.4 Hz, 1H, arom. $C_{10}H_7$), 7.62 (t., J = 7.7 Hz, 2H, arom. $C_{10}H_7$), 7.88 (d., J = 8.1 Hz, 1H, arom. $C_{10}H_7$), 8.37 (d., J = 8.3 Hz, 1H, arom. $C_{10}H_7$).

$^{13}C\{^{1}H\}$ NMR (100 MHz, DMSO-d$_6$, δ, ppm): 29.3, 31.1, 34.9, 36.5, 111.9, 115.8, 121.0, 123.6, 124.5, 126.0, 126.1, 126.3, 128.5, 129.8, 133.3, 135.3, 137.0, 142.8, 142.9, 148.5.

HR-MS: Found m/z: 403.1710 $[M+Na]^+$. $C_{24}H_{28}NaO_2S$. Calcd. m/z: 403.1702.

4,6-Di-*tert*-butyl-3-(naphthalen-1-ylsulfinyl)benzene-1,2-diol (7a)

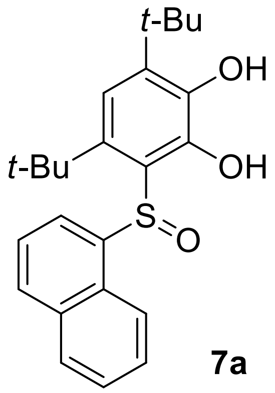

7a

Yield 0.565 g (89%). White crystalls with m.p. 216–218ºC.

IR (KBr, ν/cm^{-1}): 3223, 3059, 2984, 2965, 2953, 2907, 2869, 1699, 1600, 1593, 1569, 1560, 1507, 1482, 1468, 1406, 1366, 1342, 1291, 1266, 1223, 1201, 1171, 1027, 972, 957, 947 (S=O), 864.

^{1}H NMR (400 MHz, $CDCl_3$, δ, ppm): 1.13 (s., 9H, tBu), 1.31 (s., 9H, tBu), 6.04 (s., 1H, OH), 6.82 (s., 1H, arom. C_6H_1), 7.10 (t., 2H, arom. $C_{10}H_7$), 7.53 (dd., 2H, arom. $C_{10}H_7$), 7.84 (t., 2H, arom. $C_{10}H_7$), 8.56 (t., 1H, arom. $C_{10}H_7$), 11.42 (s., 1H, OH).

$^{13}C\{^{1}H\}$ NMR (100 MHz, $CDCl_3$, δ, ppm): 29.3, 32.6, 35.5, 36.4, 114.5, 115.9, 123.7, 125.3, 126.2, 127.2, 128.2, 129.2, 131.0, 133.1, 134.5, 136.7, 138.8, 139.6, 143.9, 149.9.

HR-MS: Found m/z: 419.1658 $[M+Na]^+$. $C_{24}H_{28}NaO_3S$. Calcd. m/z: 419.1651.

S1.4. X-ray structures

Single crystal X–ray diffraction data were collected using an in a SuperNova, Dual, Cu at home/near, AtlasS2 diffractometer (CuK/α radiation, λ = 1.54186 Å) for **1a** and **7a**, Bruker APEX II diffractometer (CCD detector, MoK/α radiation, λ = 0.71073 Å) for **4a** and **6a**, a Bruker D8 Venture diffractometer (CCD detector, MoK/α radiation, λ = 0.71073 Å) for **5a**. Semi-empirical empirical absorption corrections for all compounds were applied [4,5]. The structures of the compounds were solved by direct methods and refined in the full-matrix anisotropic approximation for all non–hydrogen atoms. The crystal structures **4a** and **7a** were solved taking into account the disordering at two positions of cyclopentyl and naphthyl fragments respectively. The hydrogen atoms of the carbon-containing ligands were positioned geometrically and refined using the riding model. The crystal structure was solved by using Olex2 packages and/or SHELXL-2014 [6–8]. The crystallographic parameters and the structure refinement statistics for **1a**, **4a**–**7a** are shown in **Table S1**. The structural data for the compounds have been deposited with the Cambridge Crystallographic Data Centre (CCDC 2534166 (**1a**), 2539526 (**4a**), 2539527 (**5a**), 2539528 (**6a**), 2534168 (**7a**)) and are available at deposit@ccdc.cam.ac.uk or http://www.ccdc.cam.ac.uk/data_request/cif.

Table S1. Experimental details and crystallographic data for compounds **1a**, **4a**–**7a**

Compound/ Parameter	**1a**	**4a**	**5a**	**6a**	**7a**
CCDC number	2534166	2539526	2539527	2539528	2534168
Empirical formula	$C_{17}H_{28}O_3S$	$C_{39.5}H_{63.5}O_6S_2$	$C_{48}H_{72}O_6S_2$	$C_{21}H_{28}O_3S$	$C_{24}H_{28}O_3S$
Formula weight	312.45	698.52	809.17	360.49	396.52
Temperature [K]	100.15	293(2)	100.00	100.00	293(2)
Crystal system	orthorhombic	monoclinic	monoclinic	orthorhombic	monoclinic
Space group (number)	$Pbca$ (61)	$P2_1/n$ (14)	$P2_1/c$ (14)	$P2_12_12_1$ (19)	$P2_1/c$ (14)
a [Å]	11.10107(17)	10.419(8)	10.5266(6)	10.1078(9)	15.0697(6)
b [Å]	9.68288(17)	29.386(16)	14.0422(8)	12.8261(12)	8.5154(3)
c [Å]	32.3713(5)	15.691(10)	29.9683(16)	15.2212(15)	19.8597(7)
β [°]	90	101.33(3)	91.149(2)	90	108.128(4)
V [Å^3]	3479.60(9)	4711(5)	4428.9(4)	1973.3(3)	2421.99(16)
Z	8	4	4	4	4
ρ_{calc} [g cm^{-3}]	1.193	0.985	1.214	1.213	1.087
μ [mm^{-1}]	1.708	0.149	0.168	0.180	1.331
F(000)	1360	1522	1760	776	848
Crystal size [mm^3]	0.34×0.27×0.21	0.7×0.18×0.08	0.15×0.08×0.06	0.3×0.27×0.24	0.36×0.246×0.06
Crystal colour	colourless	colourless	colourless	colourless	colourless
Crystal shape	block	parallelepiped	parallelepiped	prismatic	plate
Radiation	CuK_α (λ=1.54184 Å)	MoK_α (λ=0.71073 Å)	MoK_α (λ=0.71073 Å)	MoK_α (λ=0.71073 Å)	Cu K_α (λ=1.54184 Å)
2Θ range [°]	5.46 to 152.13 (0.79 Å)	4.22 to 51.36 (0.82 Å)	3.87 to 56.56 (0.75 Å)	4.84 to 66.36 (0.65 Å)	6.17 to 152.47 (0.79 Å)

Compound/ Parameter	**1a**	**4a**	**5a**	**6a**	**7a**
Index ranges	-13 ≤ h ≤ 13<br>-12 ≤ k ≤ 10<br>-40 ≤ l ≤ 40	-12 ≤ h ≤ 12<br>-35 ≤ k ≤ 35<br>-19 ≤ l ≤ 19	-14 ≤ h ≤ 12<br>-18 ≤ k ≤ 18<br>-39 ≤ l ≤ 39	-15 ≤ h ≤ 15<br>-19 ≤ k ≤ 19<br>-21 ≤ l ≤ 17	-18 ≤ h ≤ 18<br>-7 ≤ k ≤ 10<br>-24 ≤ l ≤ 24
Reflections collected	26013	42998	42302	15579	40282
Independent reflections	3617<br>R_{int} = 0.0513<br>R_{sigma} = 0.0264	8917<br>R_{int} = 0.0812<br>R_{sigma} = 0.0677	10977<br>R_{int} = 0.0948<br>R_{sigma} = 0.0881	6725<br>R_{int} = 0.0325<br>R_{sigma} = 0.0435	5047<br>R_{int} = 0.0468<br>R_{sigma} = 0.0223
Completeness	99.9 %	99.7 %	99.9 %	99.0 %	100.0 %
Data / Restraints / Parameters	3617/0/206	8917/139/512	10977/0/521	6725/0/235	5047/169/366
Goodness-of-fit on F^2	1.067	1.151	1.068	1.046	1.045
Final R indexes [I≥2σ(I)]	R_1 = 0.0652<br>wR_2 = 0.1697	R_1 = 0.1158<br>wR_2 = 0.2716	R_1 = 0.0656<br>wR_2 = 0.1337	R_1 = 0.0344<br>wR_2 = 0.0859	R_1 = 0.0469<br>wR_2 = 0.1325
Final R indexes [all data]	R_1 = 0.0672<br>wR_2 = 0.1707	R_1 = 0.1591<br>wR_2 = 0.2965	R_1 = 0.0972<br>wR_2 = 0.1467	R_1 = 0.0368<br>wR_2 = 0.0871	R_1 = 0.0527<br>wR_2 = 0.1414
Largest peak/hole [eÅ^{-3}]	0.91/-0.41	0.86/-0.28	0.66/-0.41	0.50/-0.24	0.22/-0.37
Flack X parameter	-	-	-	0.50(6)	-

Table S2: Selected bond lengths (Å) and bond angles (°) of **1a**, **4a**–**7a**.

Compound/ Parameter	**1a**	**4a**	**5a**	**6a**	**7a**
C(cat)-C(cat)	1.390(4)-1.404(4)	1.390(7)-1.415(7)	1.387(3)-1.405(3)	1.397(2)-1.404(2)	1.395(2)-1.406(2)
O1(OH)–C(cat)	1.362(4)	1.358(5), 1.373(6)	1.360(3), 1.360(3)	1.364(18)	1.353(18)
O2(OH)–C(cat)	1.364(4)	1.360(6), 1.363(6)	1.371(3), 1.367(3)	1.364(18)	1.351(2)
C(^{t}Bu)-C(cat)	1.534(4), 1.545(4)	1.539(7)-1.576(7)	1.530(3)-1.557(3)	1.537(2), 1.546(2)	1.539(2), 1.539(2)
S–C(cat)	1.789(3)	1.788(5), 1.795(5)	1.789(2), 1.788(2)	1.784(15)	1.782(15)
S–O	1.532(2)	1.512(4), 1.526(4)	1.528(17), 1.527(17)	1.525(11)	1.519(14)
S–C(R)	1.838(3)	1.846(7), 1.811(7)	1.865(2), 1.892(2)	1.838(15)	1.810(8), 1.797(10)
O–S–C(cat)	105.37(13)	105.7(2), 104.2(2)	105.69(10), 105.03(10)	106.75(7)	105.85(7)
O–S–C(R)	104.26(13)	110.0(3), 103.6(3)	105.37(10), 106.30(10)	105.16(7)	108.9(4), 100.9(5)
C(cat)–S–C(R)	100.16(14)	100.5(3), 101.1(3)	104.45(11), 103.30(11)	97.00	103.4(3) 100.8(4)
C(OH)–C(cat)–S	115.2(2)	116.7(3), 115.2(4)	116.92(18), 116.38(18)	116.33(11)	116.15(11)
C(OH)–C(cat)–S	124.5(2)	123.3(3), 124.9(4)	122.95(18), 123.28(17)	122.93(11)	123.22(13)
C(R)–S–C(cat)–C(OH)	66.7(2)	83.3(5), 76.4(9)	77.4(2), 72.4(2)	74.35(12)	84.3(5)

Table S3. C-H···A interactions in crystals of **1a**, **4a**–**7a**

Interaction	Symmetry	C–H, Å	H···A, Å	C···A, Å	C–H–A, deg.
**1a**					
O16-H16...O17	-	0.79(4)	2.17(4)	2.630(3)	118(3)
O16-H16...O18	3/2-x, -1/2+y, z	0.79(4)	2.13(4)	2.808(3)	145(4)
O17-H17...S1		0.78(4)	2.50(3)	2.957(3)	119(3)
O17-H17...O18		0.78(4)	1.91(4)	2.639(3)	156(3)
C10-H10A...S1		0.98	2.61	3.308(3)	129
C11-H11C...S1		0.98	2.63	3.357(4)	131
C13-H13C...O16		0.98	2.41	3.054(4)	123
C14-H14A...O16		0.98	2.29	2.958(4)	125
C19-H19...O17		1.00	2.49	3.141(4)	123
C21-H21B...O17	1-x, -1/2+y, 1/2-z	0.98	2.58	3.190(4)	120
**4a**					
O2-H2 ...S1		0.82	2.64	2.988(4)	108
O2-H2 ...O1		0.82	1.87	2.563(6)	141
O3-H3 ...O2		0.82	2.18	2.640(5)	116
O3-H3 ...O4	1/2+x, 3/2-y, 1/2+z	0.82	2.01	2.774(6)	154
O5-H5 ...S2		0.82	2.43	2.935(4)	121
O5-H5 ...O4		0.82	1.83	2.579(6)	151
O6-H6 ...O5		0.82	2.19	2.649(6)	116
O6-H6 ...O1	1/2+x, 3/2-y, -1/2+z	0.82	1.99	2.758(6)	155
C9-H9C...O3		0.96	2.36	3.019(10)	126
C10-H10A..O3		0.96	2.35	2.993(7)	124
C12-H12C..S1		0.96	2.49	3.218(7)	132
C13-H13B..S1		0.96	2.66	3.374(7)	131
C19-H19A..O1		0.97	2.57	3.114(12)	115
C27-H27A..O6		0.96	2.32	2.989(9)	126
C29-H29B..O6		0.96	2.36	3.012(8)	124
C31-H31A..S2		0.96	2.59	3.281(7)	130
C32-H32B..S2		0.96	2.70	3.407(8)	131
C34-H34...O5		0.98	2.47	3.125(8)	124
**5a**					
O1-H1...S1		0.84	2.54	2.9982(17)	115
O1-H1...O3		0.84	1.80	2.573(2)	152
O2-H2...O1		0.84	2.21	2.651(2)	113
O2-H2...O6		0.84	2.02	2.790(2)	152
O4-H4A ...S2		0.84	2.53	2.9569(17)	113
O4-H4A ...O6		0.84	1.85	2.608(2)	150

**Interaction**	Symmetry	**C–H, Å**	**H···A, Å**	**C···A, Å**	**C–H–A, deg.**
O5-H5...O3	-1+x, y, z	0.84	2.03	2.803(2)	153
O5-H5...O4		0.84	2.19	2.662(2)	115
C9-H9B ...O2		0.98	2.39	3.042(3)	124
C10-H10C...O2		0.98	2.35	3.015(3)	124
C12-H12A...S1		0.98	2.72	3.429(2)	130
C13-H13C...S1		0.98	2.62	3.311(3)	127
C34-H34A...O5		0.98	2.36	3.024(3)	124
C35-H35C...O5		0.98	2.35	3.011(3)	124
C37-H37A...S2		0.98	2.55	3.284(3)	131
C38-H38C...S2		0.98	2.65	3.354(3)	129
C44-H44A...O1		0.99	2.51	3.397(3)	148
C47-H47A...O4		0.99	2.49	3.255(3)	134
**6a**					
O1-H1...S1		0.84	2.50	2.9642(12)	115
O1-H1...O3		0.84	1.81	2.5864(16)	152
O2-H2...O1		0.84	2.21	2.6537(16)	113
O2-H2...O3	1/2+x, 1/2-y, 1-z	0.84	2.13	2.9306(16)	159
C9-H9A...O2		0.98	2.36	3.028(2)	125
C10-H10C...O2		0.98	2.36	3.013(2)	123
C12-H12C...S1		0.98	2.65	3.3421(17)	128
C13-H13A...S1		0.98	2.53	3.2219(17)	127
**7a**					
O1-H1...O3		0.87(3)	1.72(3)	2.5498(19)	158(3)
O2-H2...O1		1.02(3)	2.23(3)	2.632(2)	102(2)
O2-H2...O3	1-x, 1/2+y, 3/2-z	1.02(3)	1.74(3)	2.7240(19)	160(3)
C8-H8A...O2		0.96	2.40	3.043(3)	124
C9-H9C...O2		0.96	2.28	2.948(3)	126

Table S4. C–X···π interactions in **1a**, **4a**–**7a** (C_g is centroid of aromatic 5,6-membered ring; X-Perp is perpendicular distance of X on ring; γ is angle X→C_g vector and normal to ring plane).

**Interaction**	**Symmetry**	**X···Cg, Å**	**X–Perp, Å**	**γ, deg.**	**C–X···Cg, deg.**	**C···Cg, Å**
**4a**						
C27-H27B…Cg(C1-C6)		2.97	2.96	5.07	139	3.748(8)
**6a**						
C13-H13C…Cg(C16-C21)	1-x, 1/2+y, 3/2-z	2.84	2.79	11.27	162	3.7886(19)
**7a**						
C16A-H16A…Cg(C1-C6)		2.86	2.32	35.70	142	3.643(17)

S1.5. Antioxidant activity assay

S1.5.1. DPPH radical scavenging activity assay

DPPH radical scavenging activity was performed according to the known method [9] with some modifications [10]. A CH_3CN solution (C_0 = 50 μmol) of the radical DPPH was prepared daily and protected from light. The decrease in absorbance was determined at 517 nm every minute during the first five minutes of the experiment and every next 5 min until the reaction reached a plateau at room temperature. The parameter EC_{50} is the concentration of an antioxidant necessary for decreasing the amount of DPPH radical by 50% of the initial value. To determine IC_{50}, the plot of the residual concentration of the stable radical vs molar ratio, expressed as the number of moles of the antioxidant per 1 mole of the DPPH, was constructed. The parameter (n_{DPPH}) is the number of molecules of converted DPPH radical per one molecule of the compound ($n_{DPPH} = C_0/(2 \times IC_{50})$, where C_0 is the initial concentration of radical). TEC_{50} is the time of achievement of an equilibrium state at the antioxidant concentration equal to IC_{50}. The antiradical efficiency (AE) was determined with the equation $AE = 1/(IC_{50} \times TEC_{50})$. All experiments were performed in triplicate at room temperature.

S1.5.2. ABTS assay

The radical cation $ABTS^{\cdot+}$ is generated by the oxidation of ABTS with the $K_2S_2O_8$. The reduction in the intensity of greenish coloration characteristic of this radical reflects the ability of the antioxidants to scavenge the radical cation [11]. The absorbance of ABTS radical cation solutions (λ = 734 nm) in the presence of compounds concentrations (1–40 μM) and calculation of the IC_{50} values were carried out following a previously published method [12]. Ethanol was the solvent for all compounds. The absorbance (A_i) reading was taken at room temperature exactly 1 min after initial mixing and up to 6 min. All measurements were carried out at least three times. The IC_{50} values were calculated as the minimum concentration of each sample required to inhibit 50% of the ABTS radical.

S2. NMR-spectra

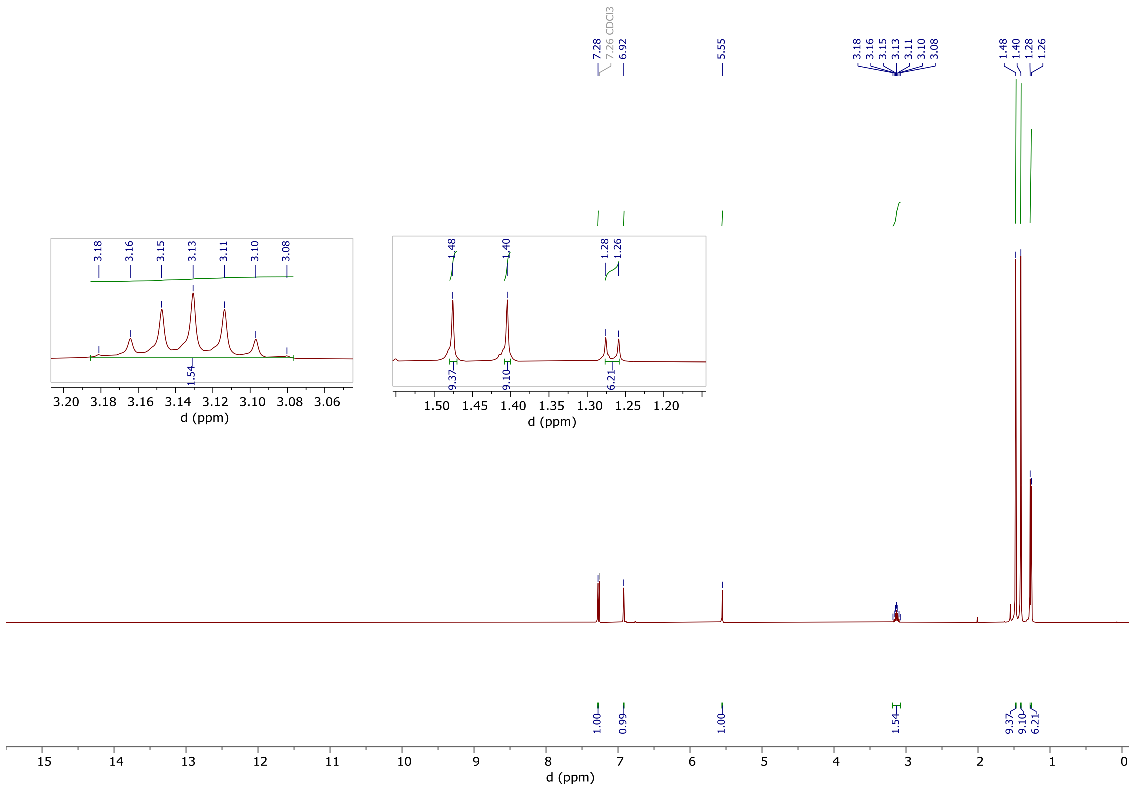

Figure S1. The ^{1}H NMR spectrum of 4,6-di-*tert*-butyl-3-(isopropylthio)benzene-1,2-diol (**1**) (400 MHz, $CDCl_3$).

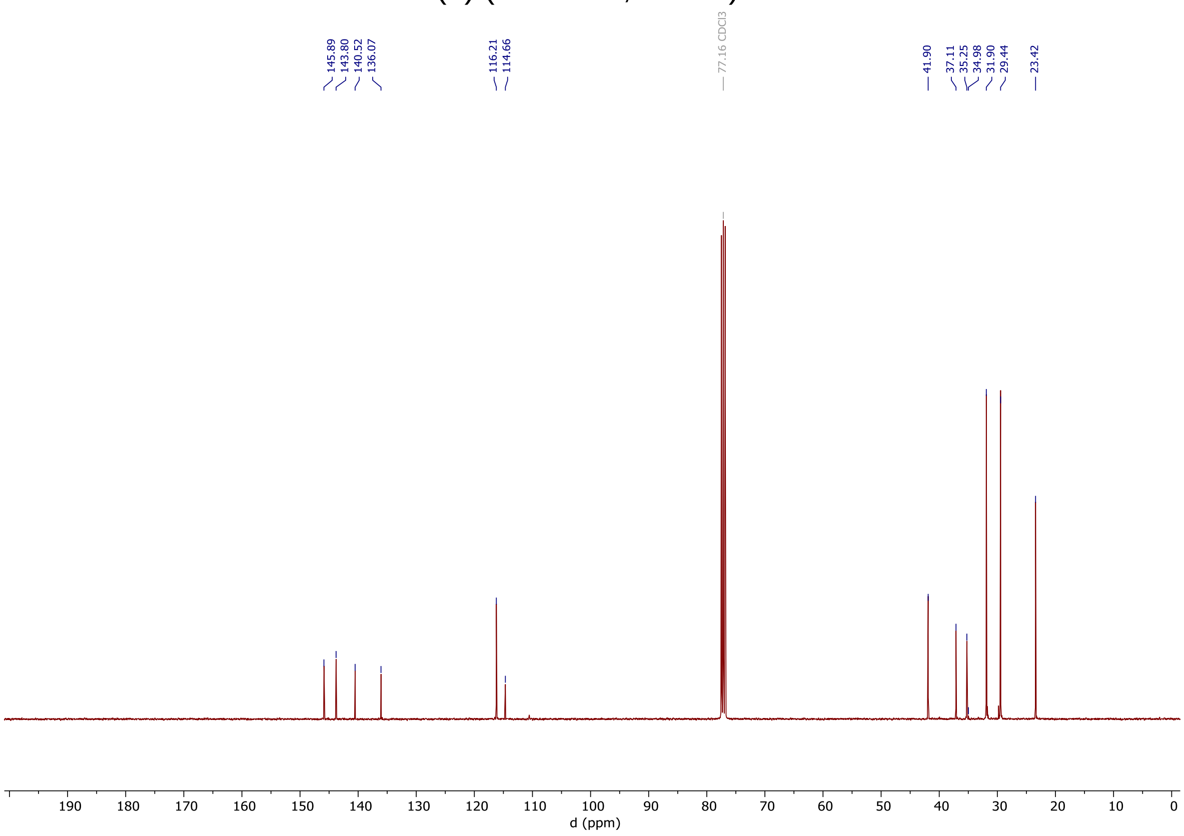

Figure S2. The $^{13}C\{^{1}H\}$ NMR spectrum of 4,6-di-*tert*-butyl-3-(isopropylthio)benzene-1,2-diol (**1**) (100 MHz, $CDCl_3$).

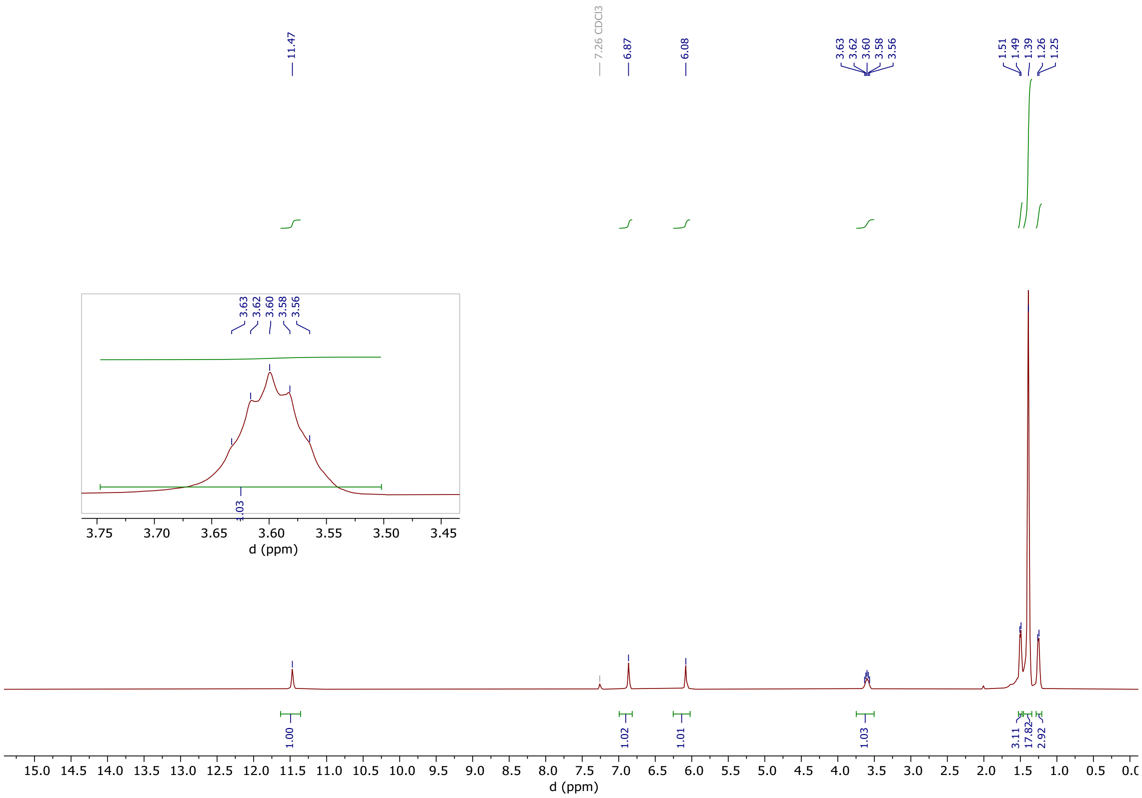

Figure S3. The ^{1}H NMR spectrum of 4,6-di-*tert*-butyl-3-(isopropylsulfinyl)benzene-1,2-diol (**1a**) (400 MHz, $CDCl_3$).

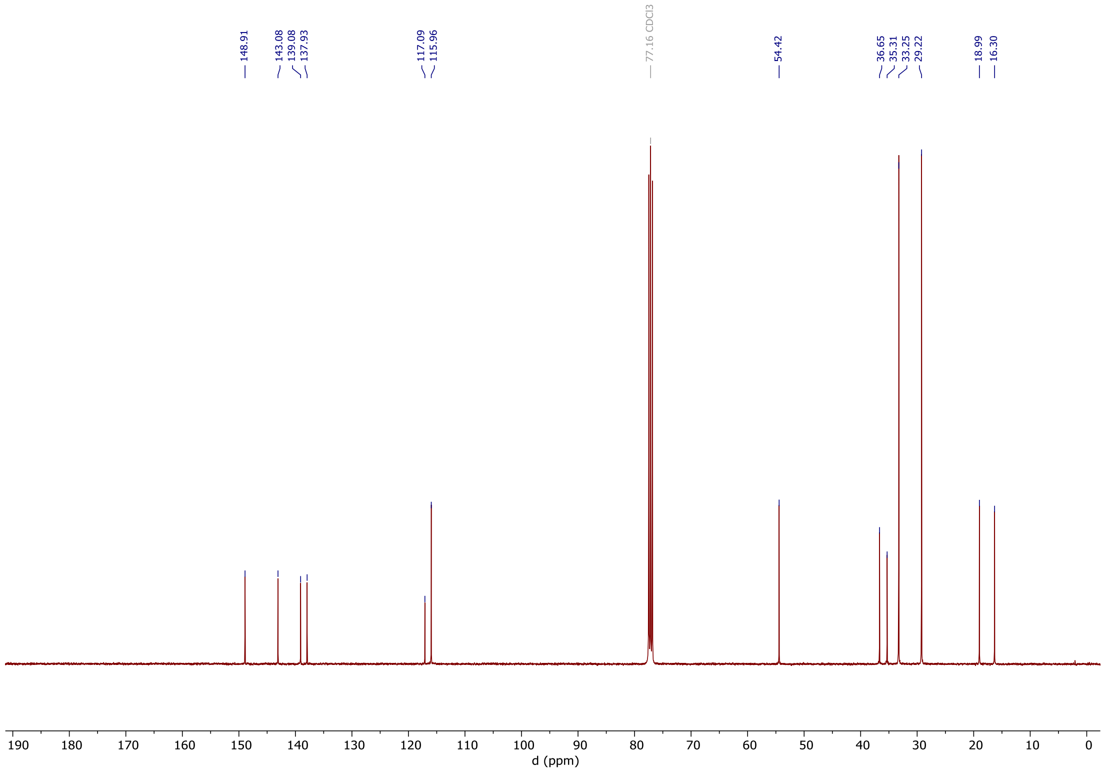

Figure S4. The ^{13}C{^{1}H} NMR spectrum of of 4,6-di-*tert*-butyl-3-(isopropylsulfinyl)benzene-1,2-diol (**1a**) (100 MHz, $CDCl_3$).

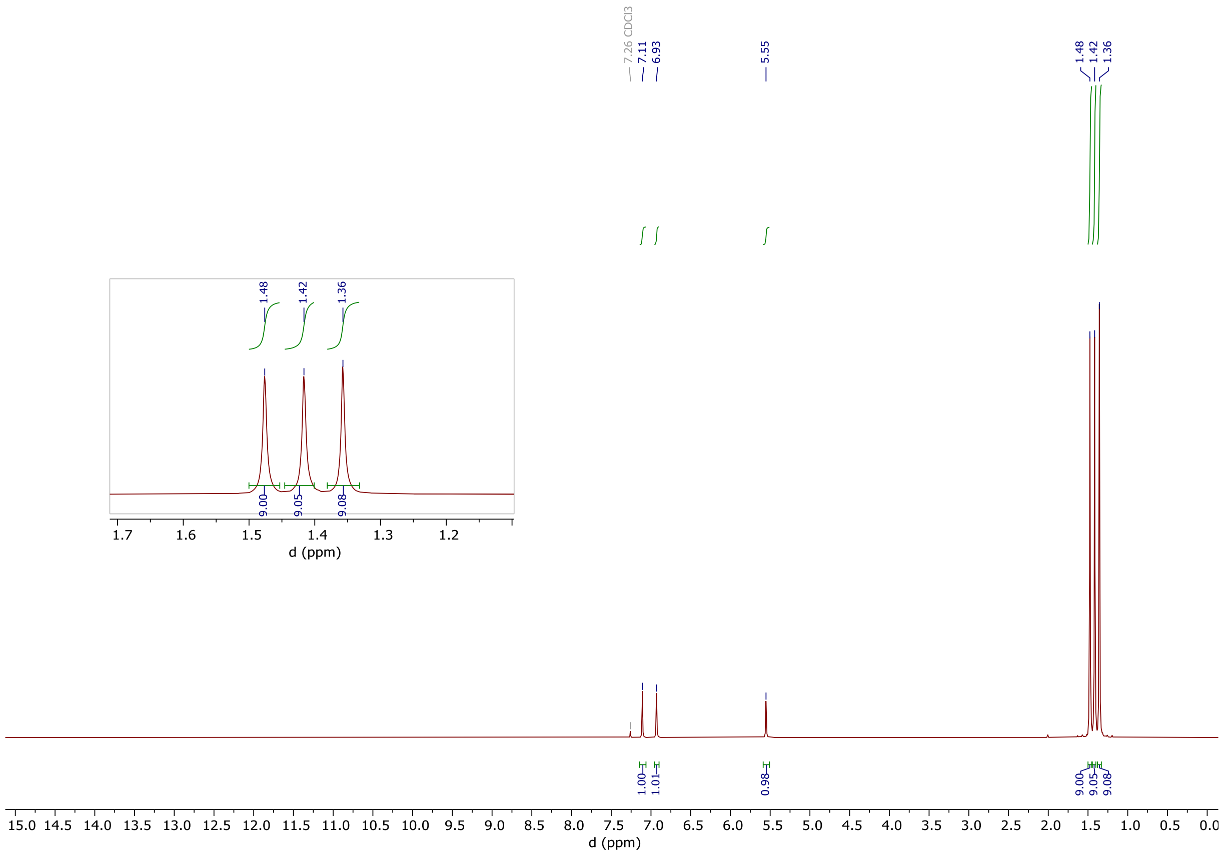

Figure S5. The ^{1}H NMR spectrum of 4,6-di-*tert*-butyl-3-(*tert*-butylthio)benzene-1,2-diol (**2**) (400 MHz, $CDCl_3$).

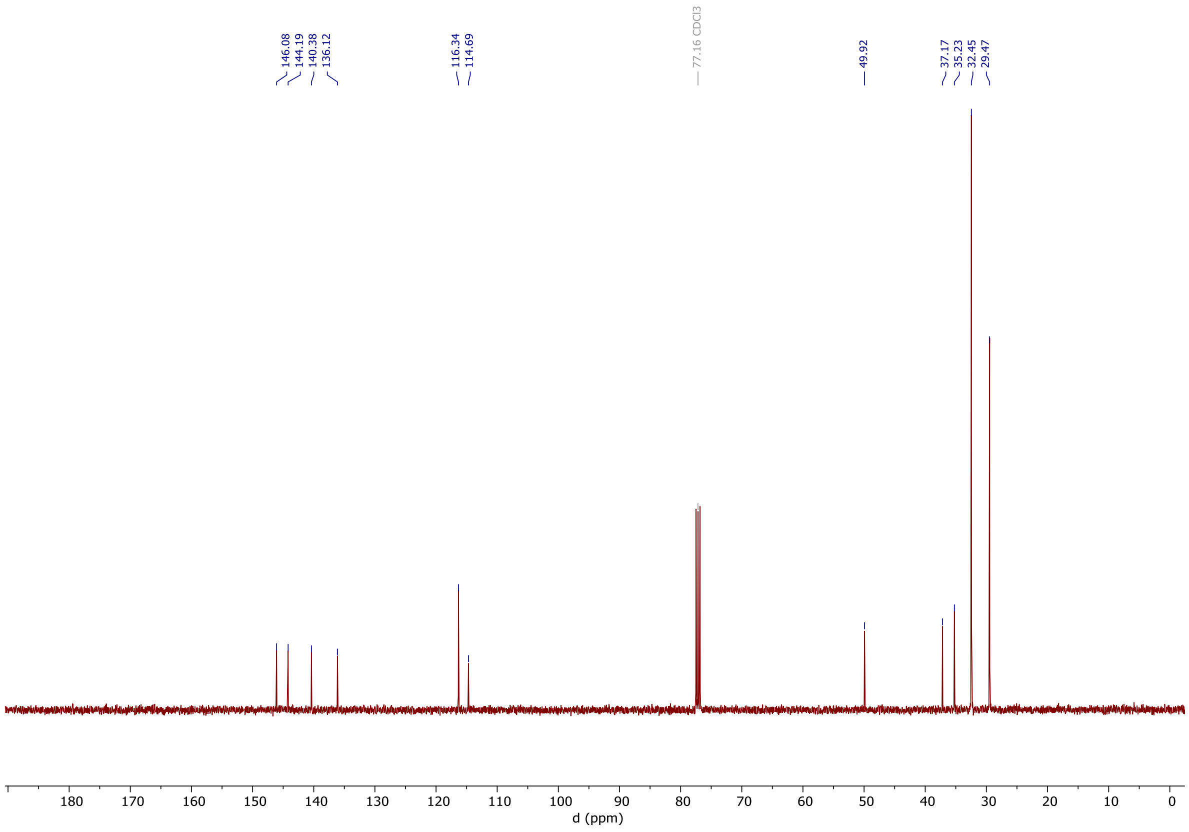

Figure S6. The $^{13}C\{^{1}H\}$ NMR spectrum of 4,6-di-*tert*-butyl-3-(*tert*-butylthio)benzene-1,2-diol (**2**) (100 MHz, $CDCl_3$).

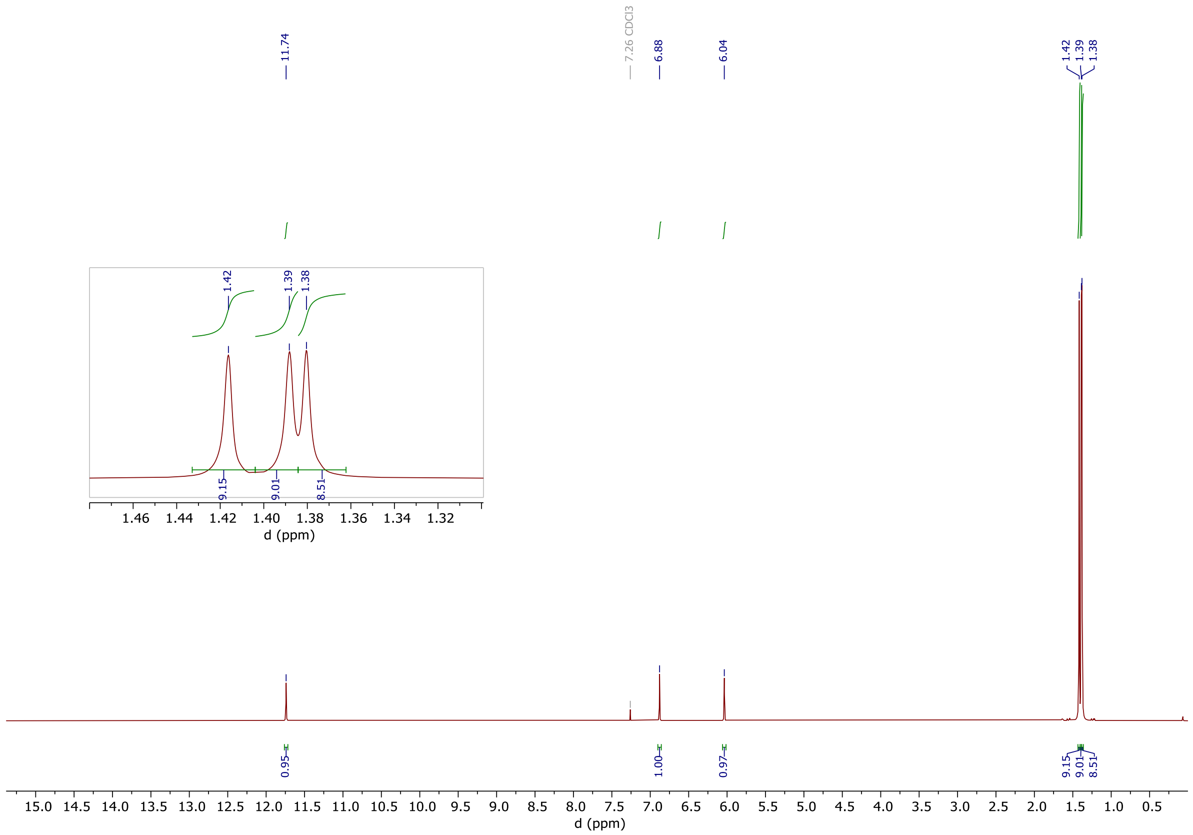

Figure S7. The ^{1}H NMR spectrum of 4,6-di-*tert*-butyl-3-(*tert*-butylsulfinyl)benzene-1,2-diol (**2a**) (400 MHz, $CDCl_3$).

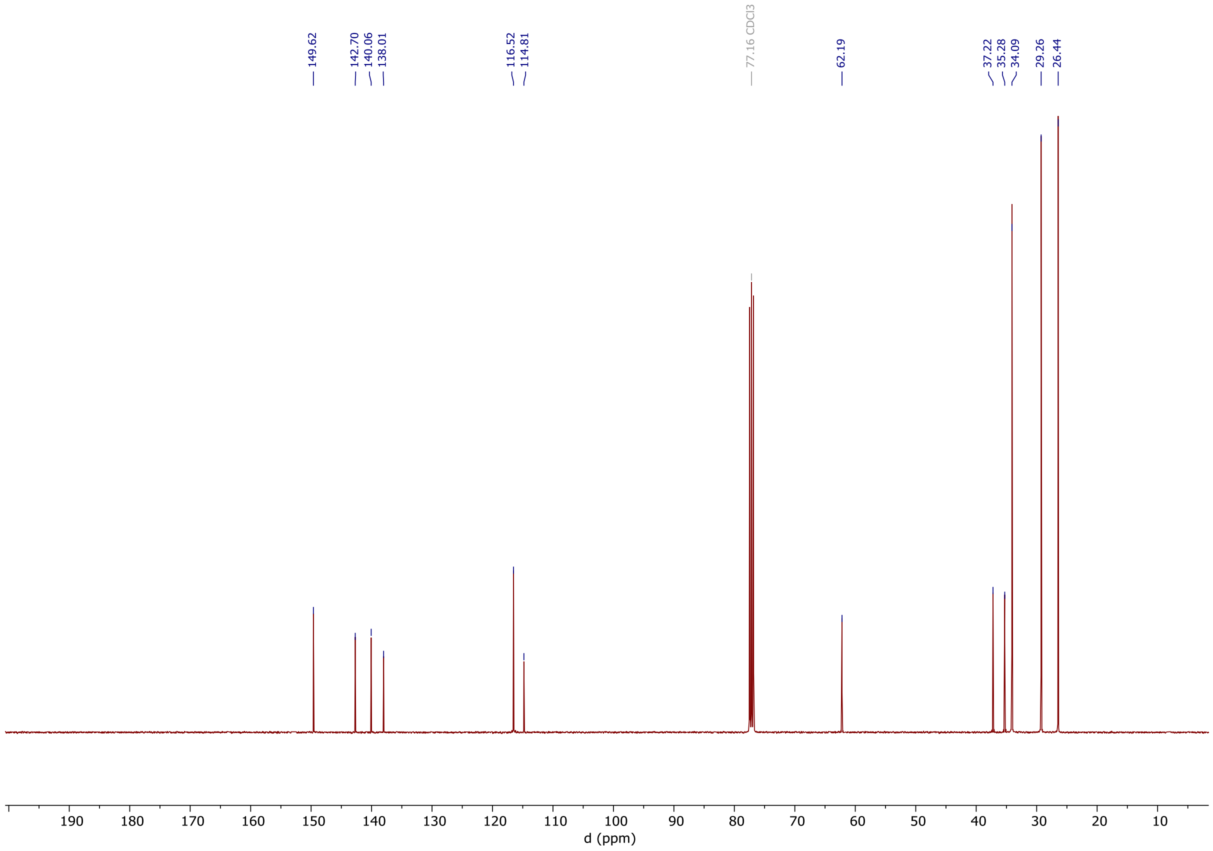

Figure S8. The $^{13}C\{^{1}H\}$ NMR spectrum of 4,6-di-*tert*-butyl-3-(*tert*-butylsulfinyl)benzene-1,2-diol (**2a**) (100 MHz, $CDCl_3$).

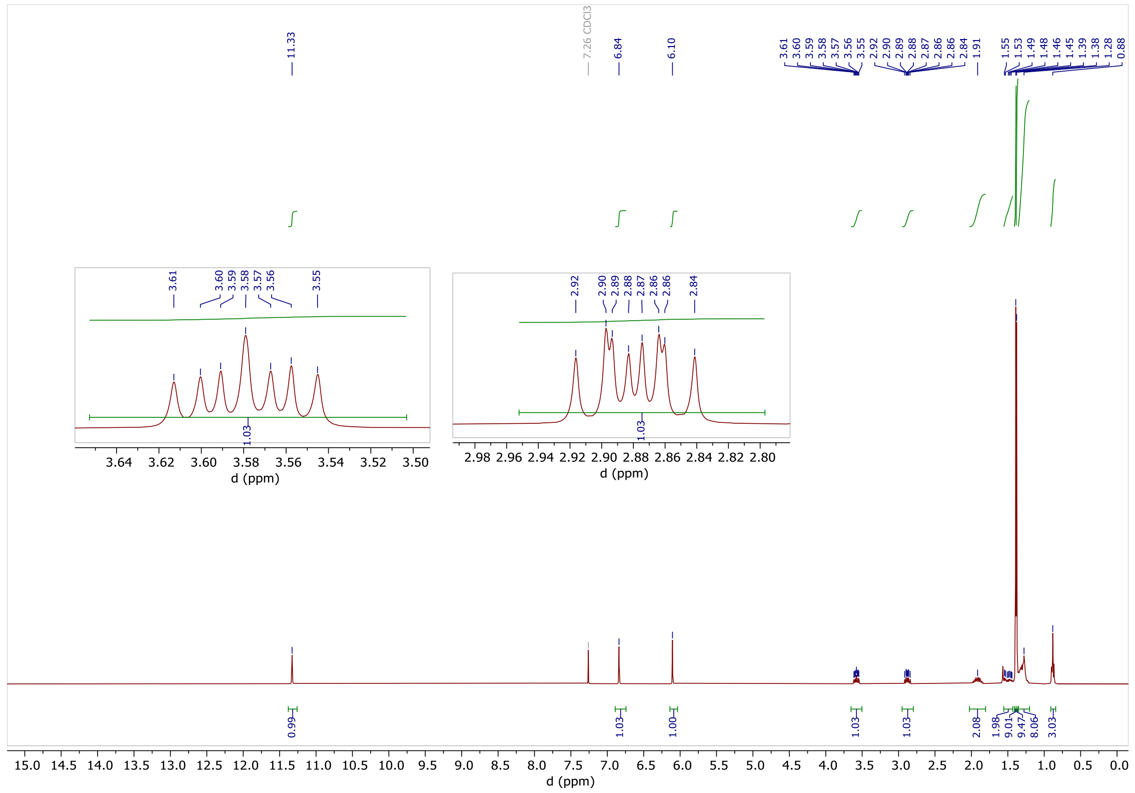

Figure S9. The ^{1}H NMR spectrum of 4,6-di-*tert*-butyl-3-(octylsulfinyl)benzene-1,2-diol (**3a**) (400 MHz, $CDCl_3$).

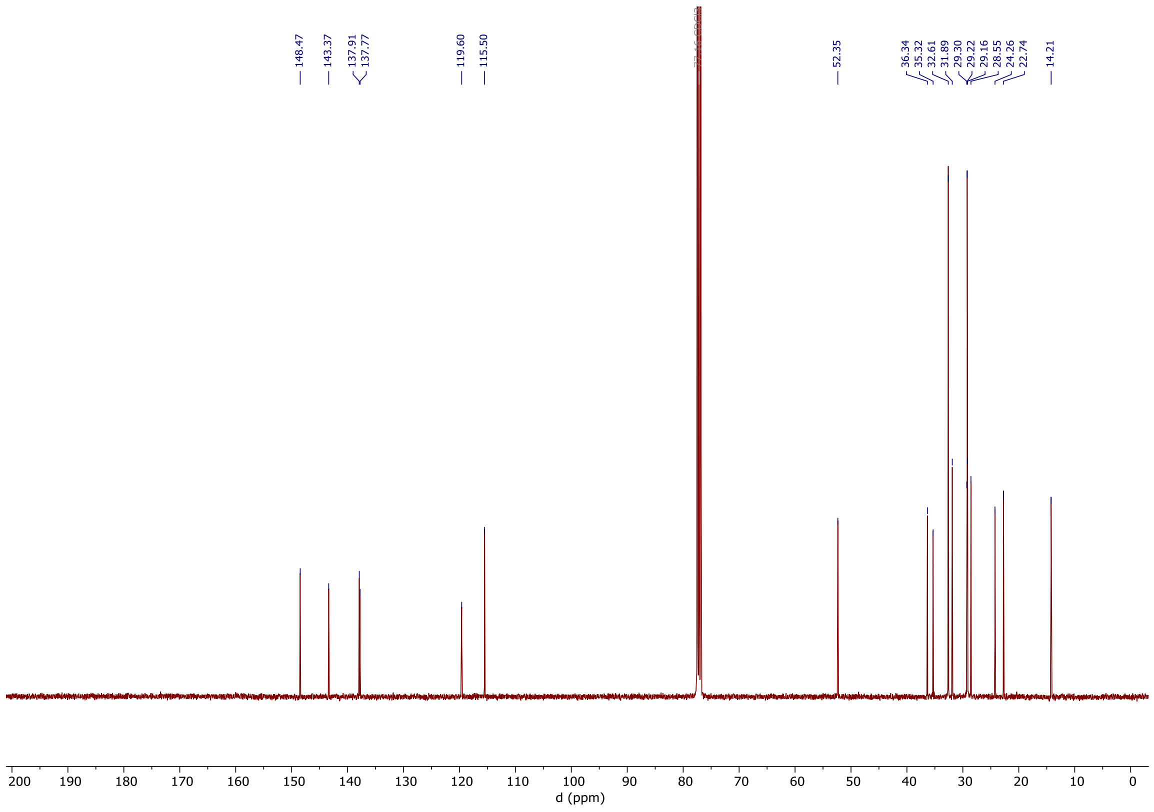

Figure S10. The $^{13}C\{^{1}H\}$ NMR spectrum of 4,6-di-*tert*-butyl-3-(octylsulfinyl)benzene-1,2-diol (**3a**) (100 MHz, $CDCl_3$).

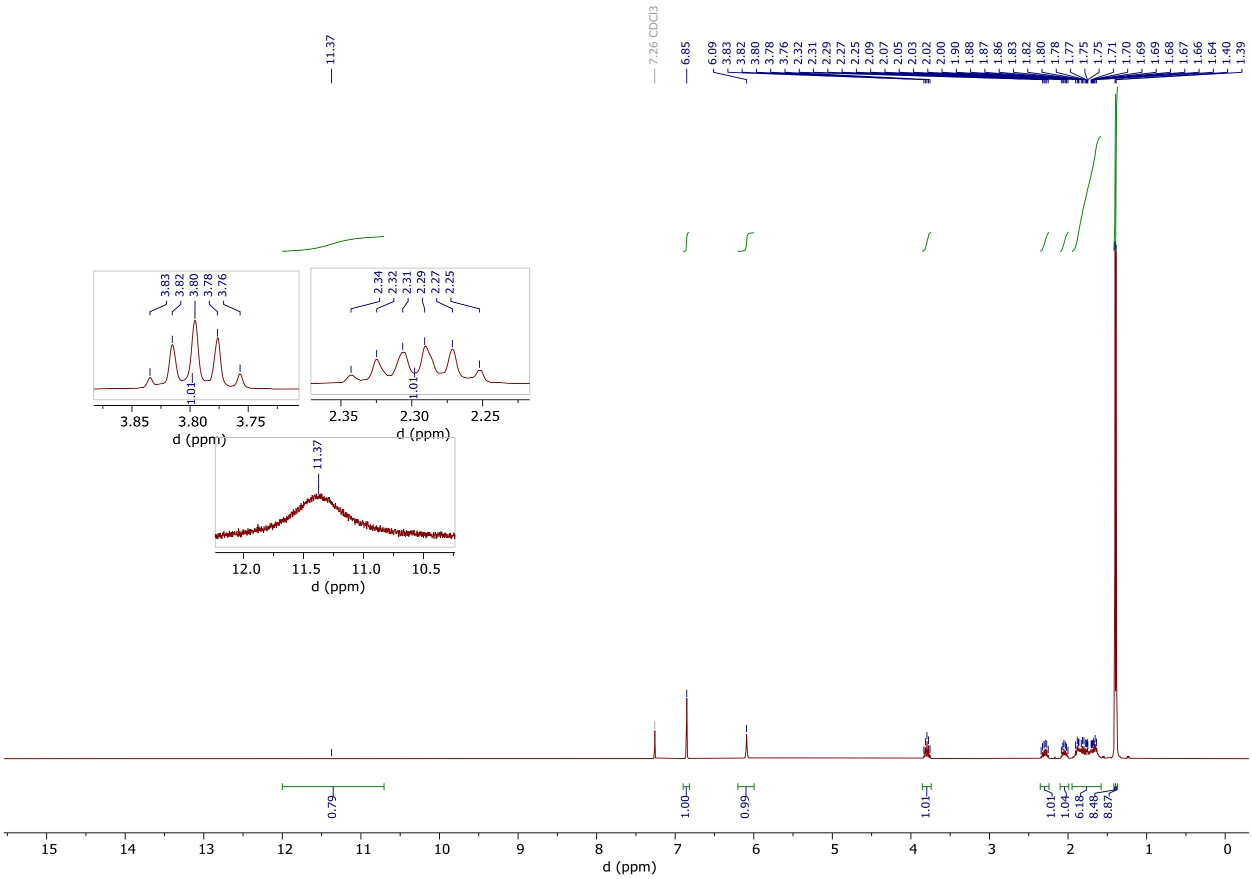

Figure S11. The ^{1}H NMR spectrum of 4,6-di-*tert*-butyl-3-(cyclopentylsulfinyl)benzene-1,2-diol (**4a**) (400 MHz, $CDCl_3$).

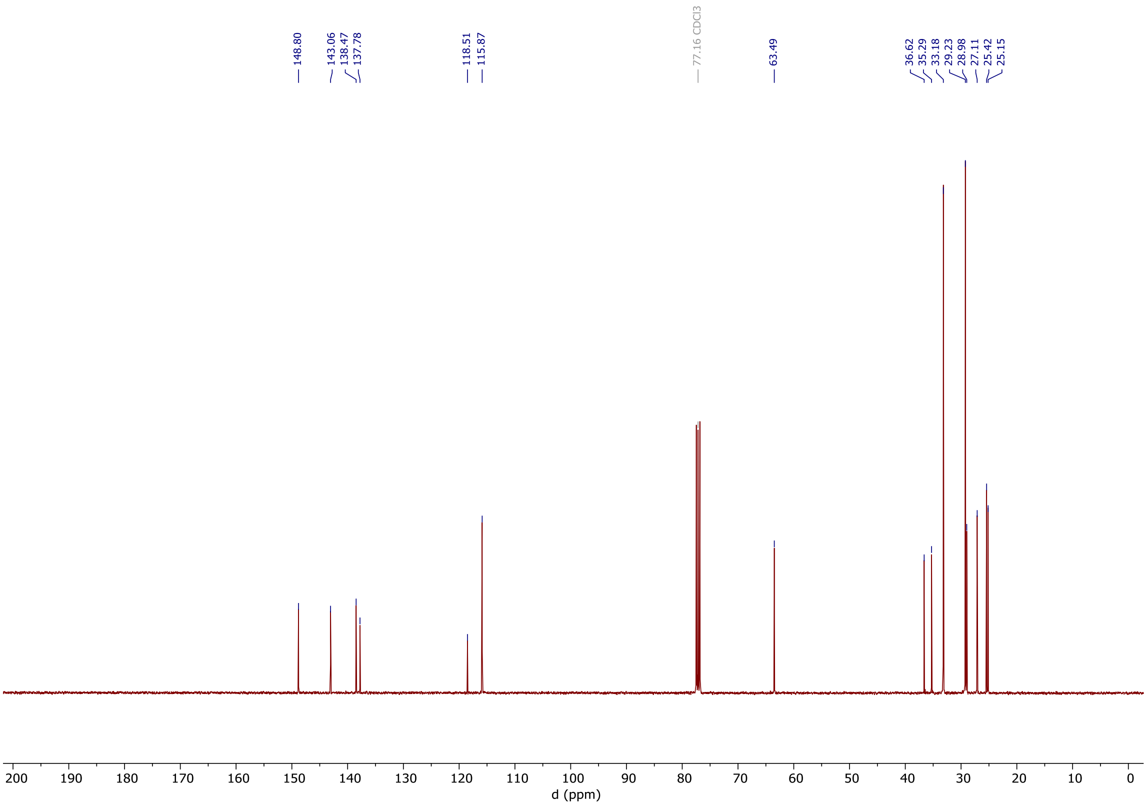

Figure S12. The $^{13}C\{^{1}H\}$ NMR spectrum of 4,6-di-*tert*-butyl-3-(cyclopentylsulfinyl)benzene-1,2-diol (**4a**) (100 MHz, $CDCl_3$).

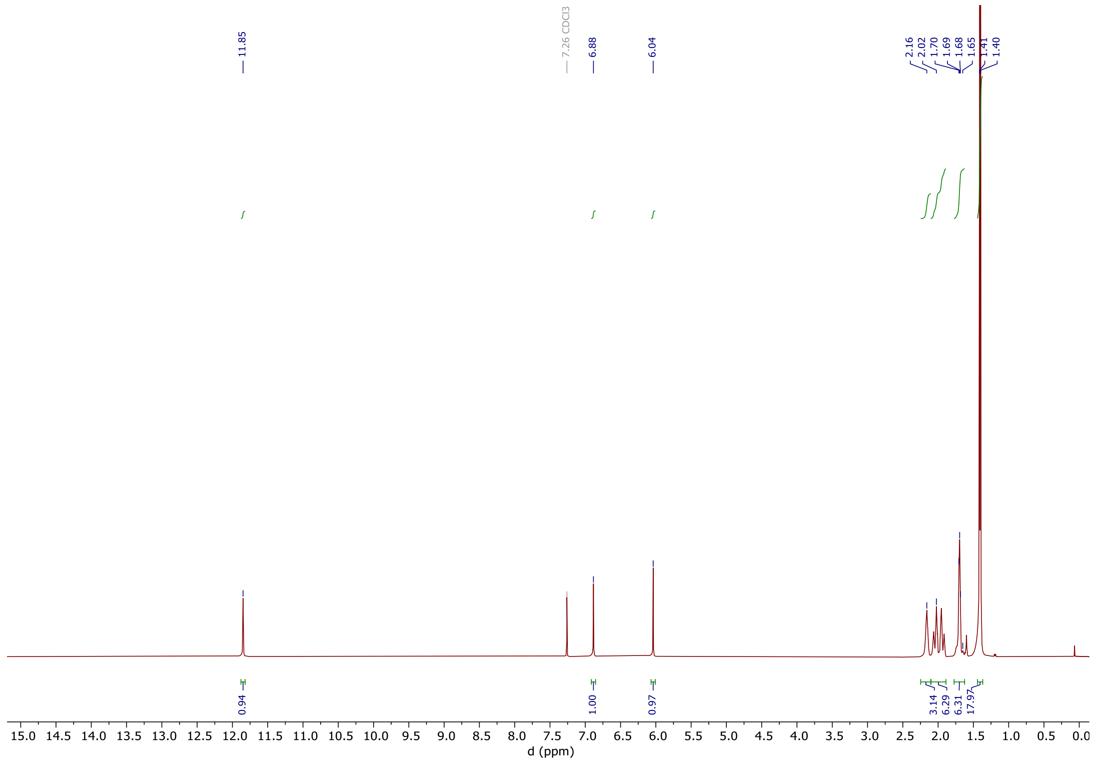

Figure S13. The ^{1}H NMR spectrum of 3-(((3s,5s,7s)-adamantan-1-yl)sulfinyl)-4,6-di-*tert*-butylbenzene-1,2-diol (**5a**) (300 MHz, $CDCl_3$).

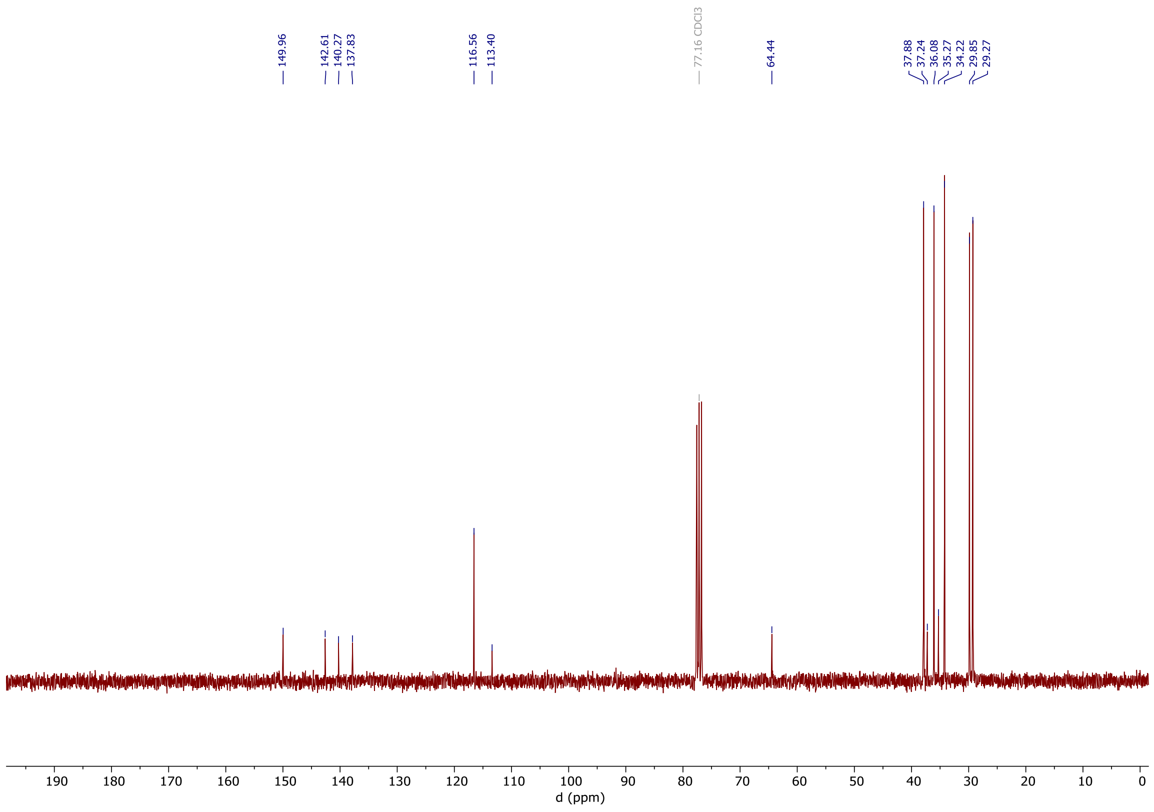

Figure S14. The ^{13}C{^{1}H} NMR spectrum of 3-(((3s,5s,7s)-adamantan-1-yl)sulfinyl)-4,6-di-*tert*-butylbenzene-1,2-diol (**5a**) (75 MHz, $CDCl_3$).

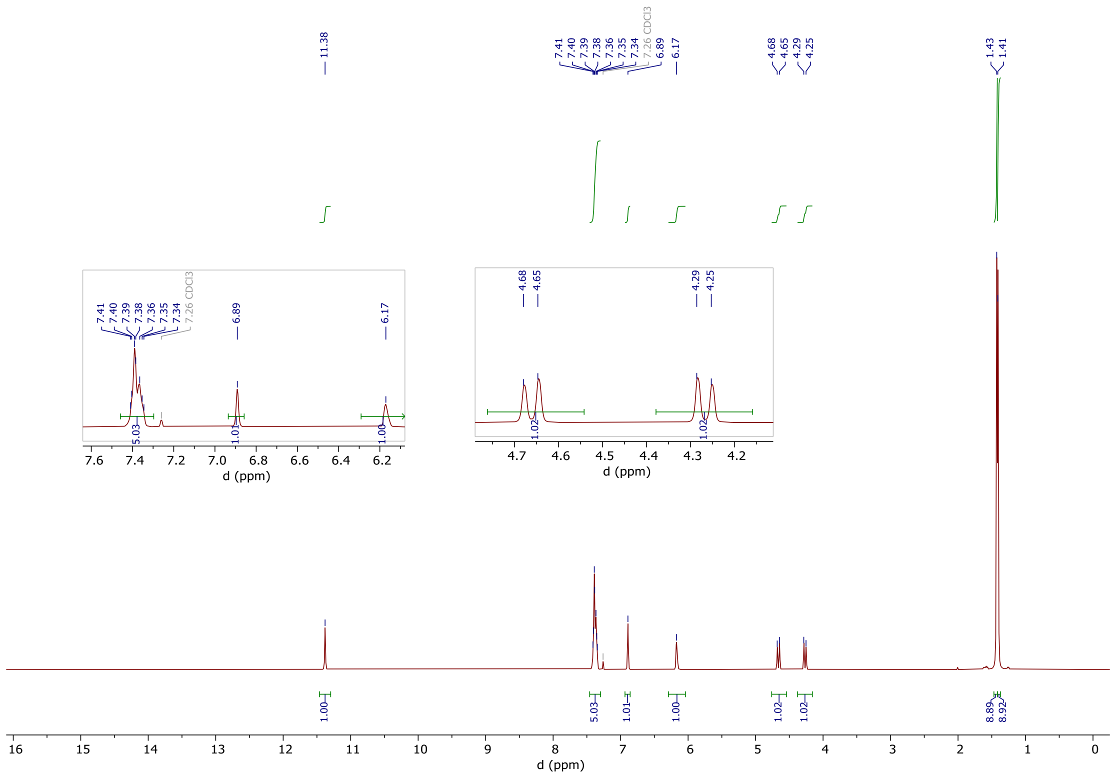

Figure S15. The ^{1}H NMR spectrum of 3-(benzylsulfinyl)-4,6-di-*tert*-butylbenzene-1,2-diol (**6a**) (400 MHz, $CDCl_3$).

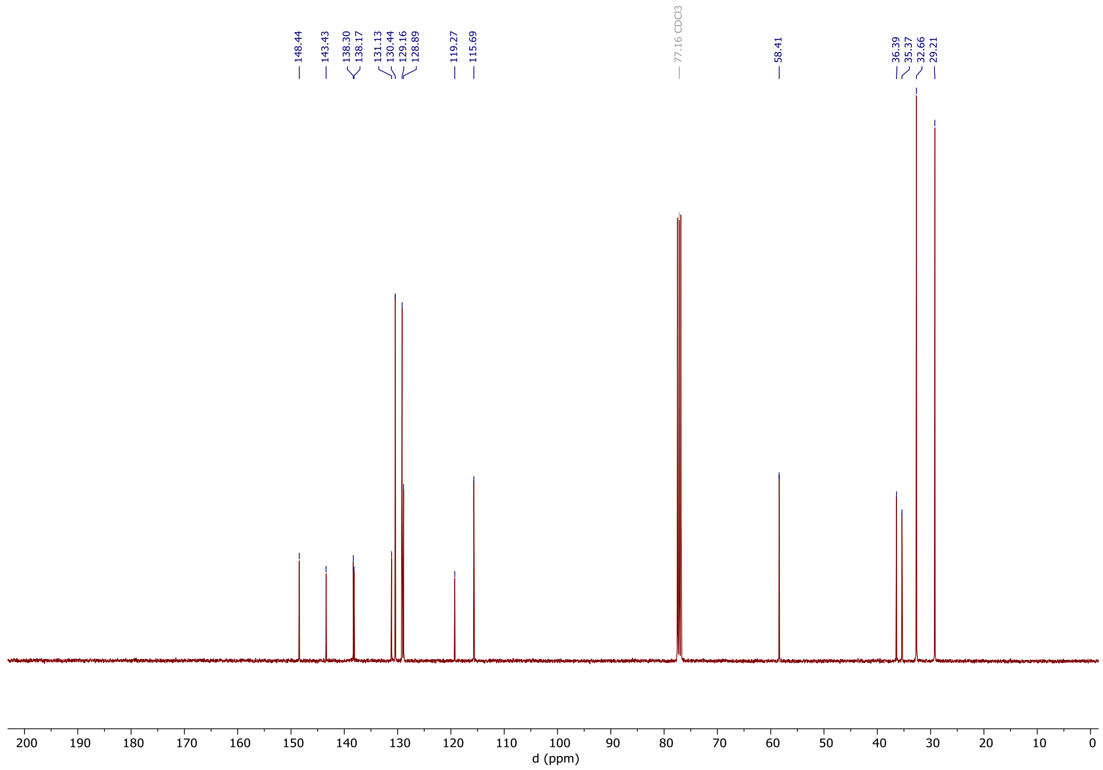

Figure S16. The $^{13}C\{^{1}H\}$ NMR spectrum of 3-(benzylsulfinyl)-4,6-di-*tert*-butylbenzene-1,2-diol (**6a**) (100 MHz, $CDCl_3$).

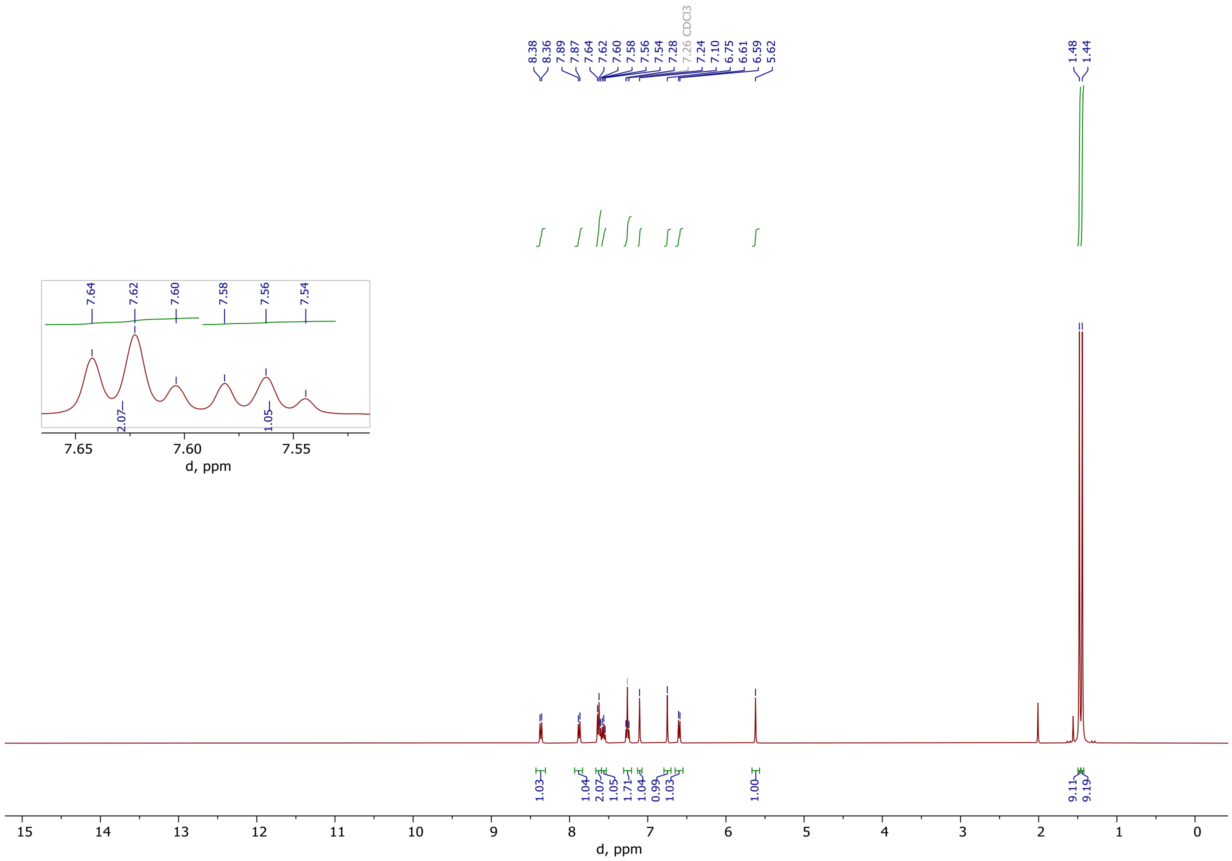

Figure S17. The ^{1}H NMR spectrum of 4,6-di-*tert*-butyl-3-(naphthalen-1-ylthio)benzene-1,2-diol (**7**) (400 MHz, $CDCl_3$).

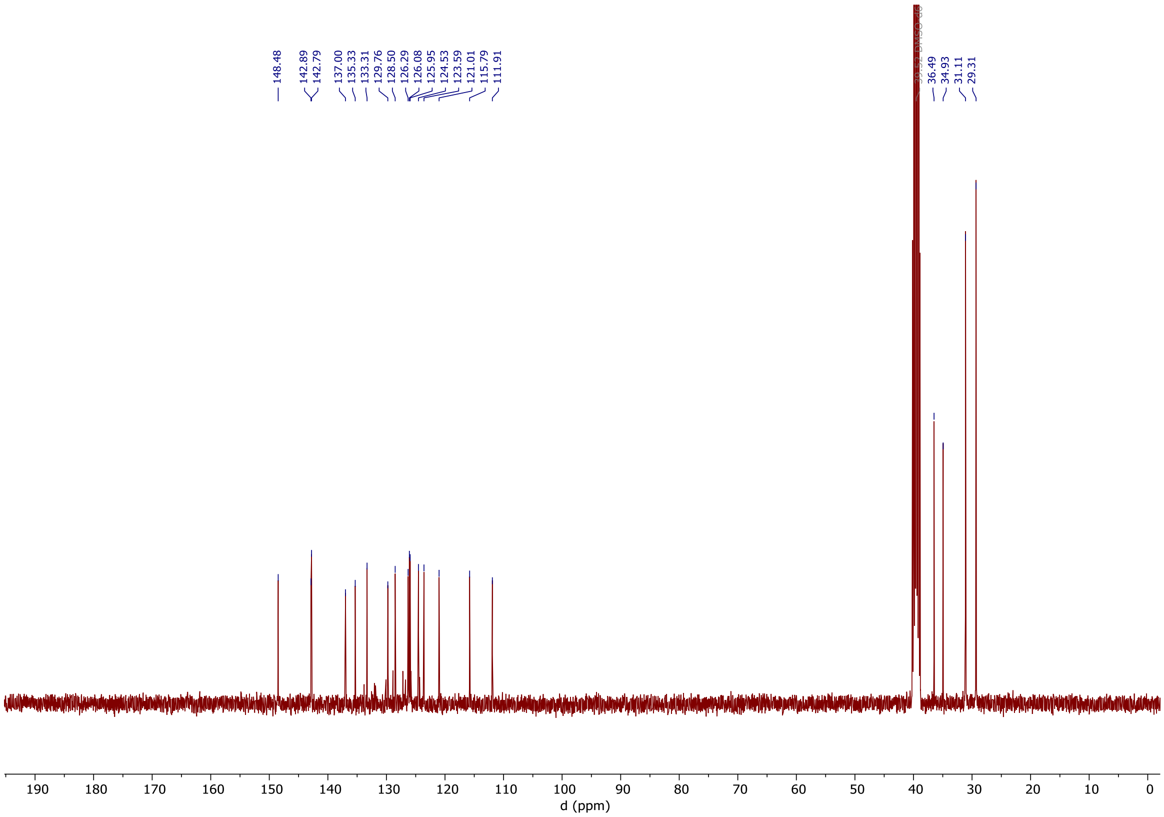

Figure S18. The $^{13}C\{^{1}H\}$ NMR spectrum of 4,6-di-*tert*-butyl-3-(naphthalen-1-ylthio)benzene-1,2-diol (**7**) (100 MHz, DMSO-d_6).

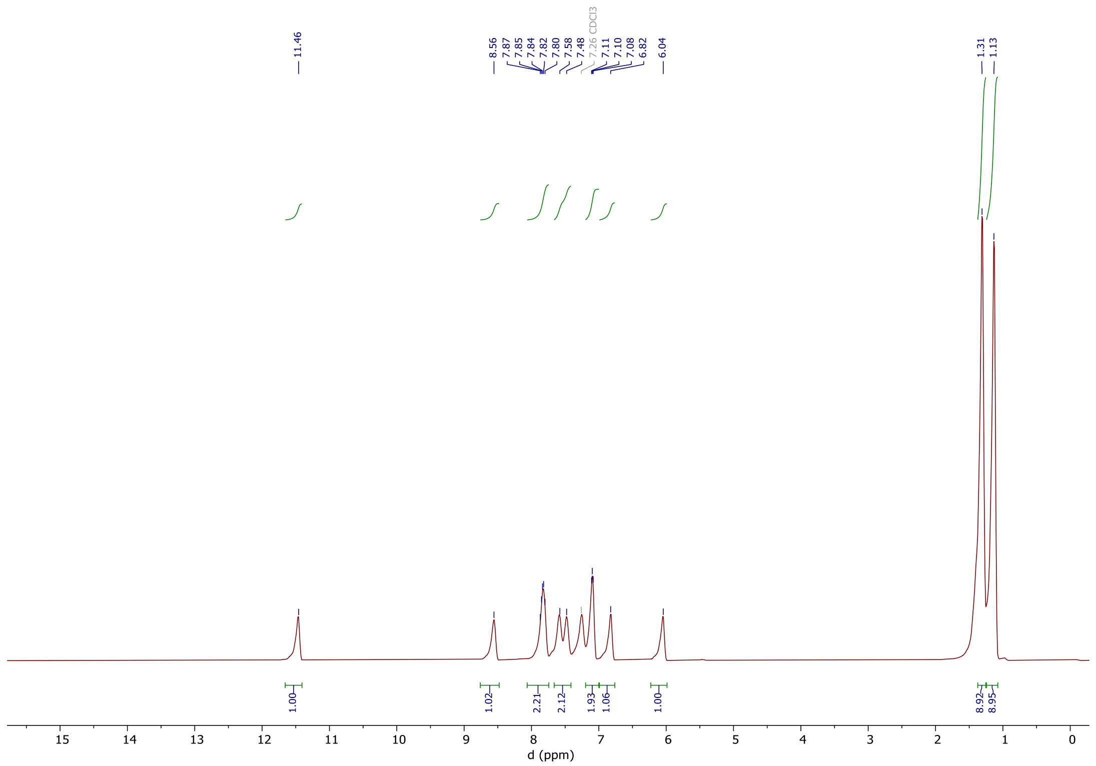

Figure S19. The ^{1}H NMR spectrum of 4,6-di-*tert*-butyl-3-(naphthalen-1-ylsulfinyl)benzene-1,2-diol (**7a**) (400 MHz, $CDCl_3$).

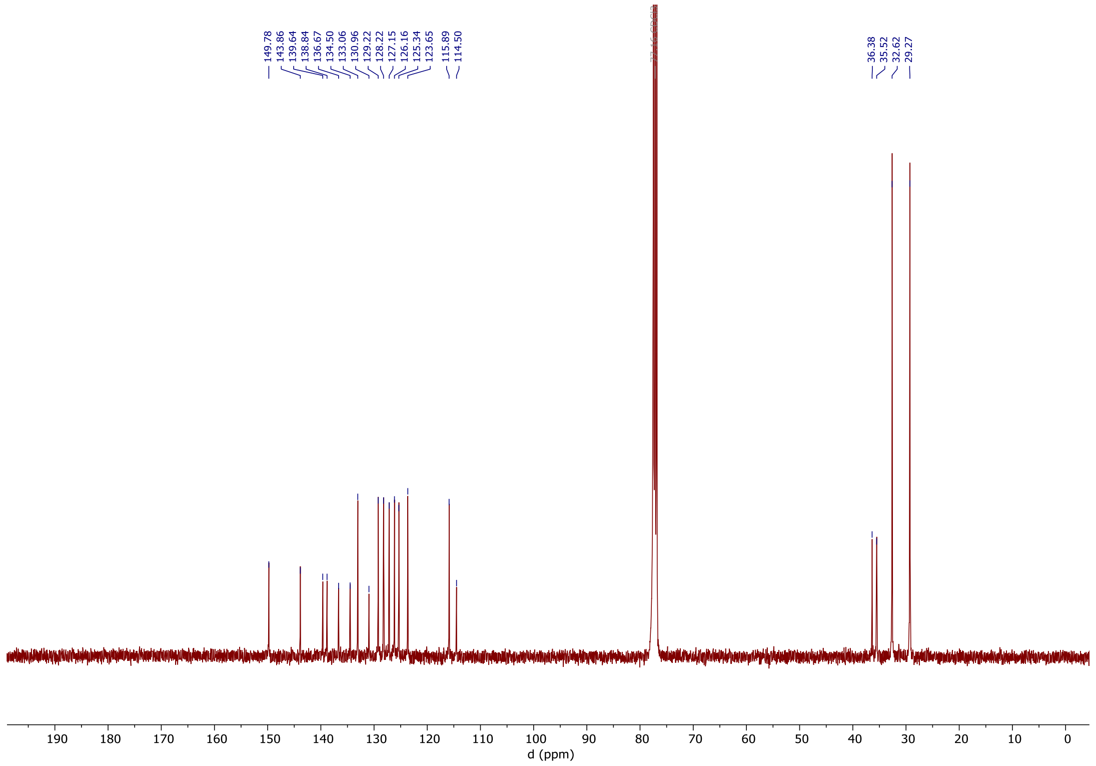

Figure S20. The $^{13}C\{^{1}H\}$ NMR spectrum of 4,6-di-*tert*-butyl-3-(naphthalen-1-ylsulfinyl)benzene-1,2-diol (**7a**) (100 MHz, $CDCl_3$).

S3. HRMS-Spectra

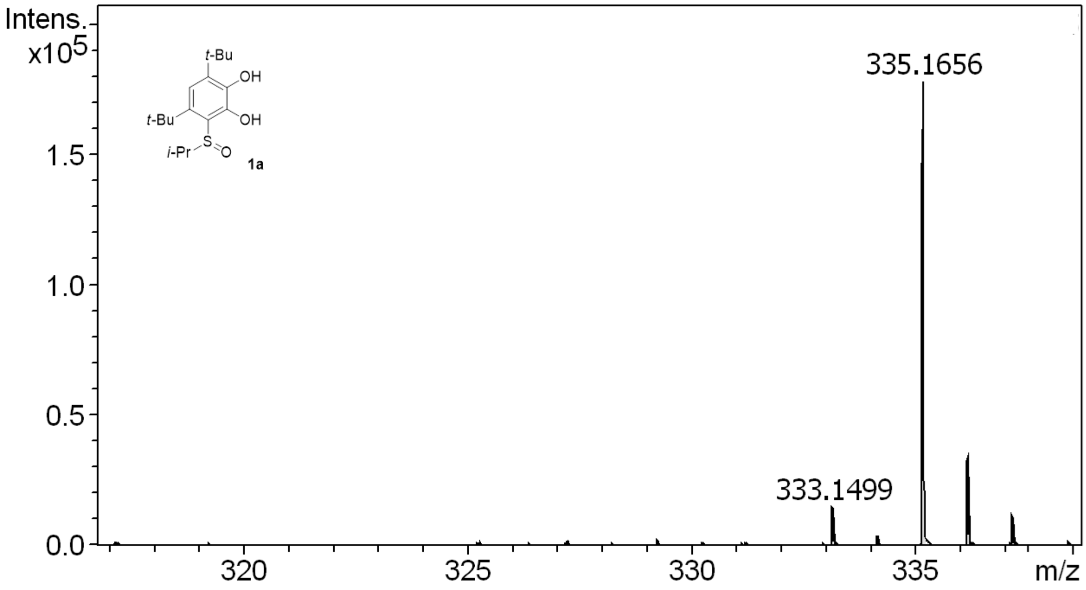

Meas. m/z	#	Ion Formula	m/z	err [ppm]	mSigma		Score	rdb	e^- Conf	N-Rule
335.1656	1	C17H28NaO3S	335.1651	-1.4	3.3	1	100.00	3.5	even	ok

Figure S21. HRMS spectra of **1a**.

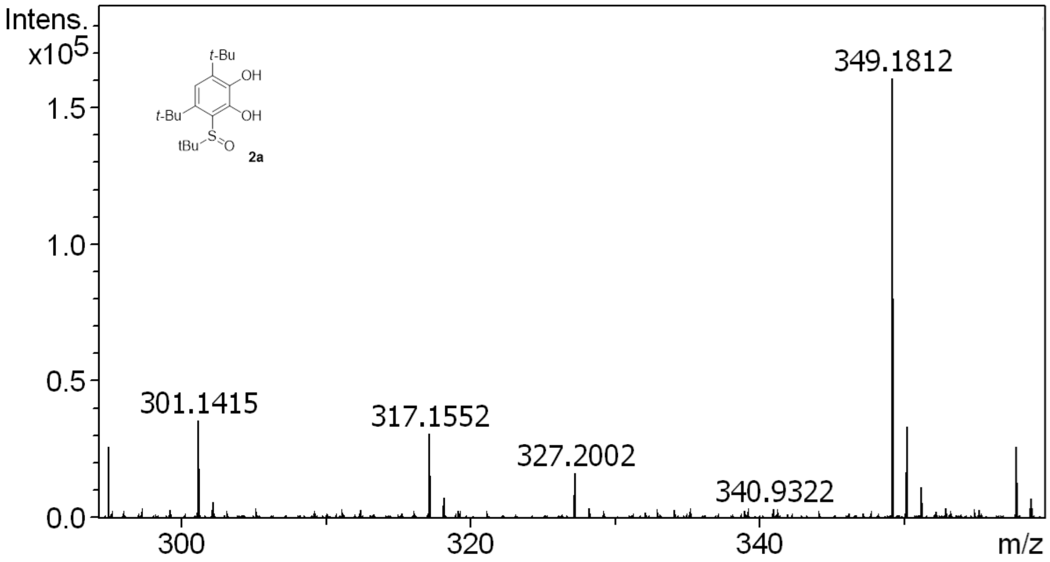

Meas. m/z	#	Ion Formula	m/z	err [ppm]	mSigma		Score	rdb	e^- Conf	N-Rule
349.1812	1	C18H30NaO3S	349.1808	-1.1	1.4	1	100.00	3.5	even	ok

Figure S22. HRMS spectra of **2a**.

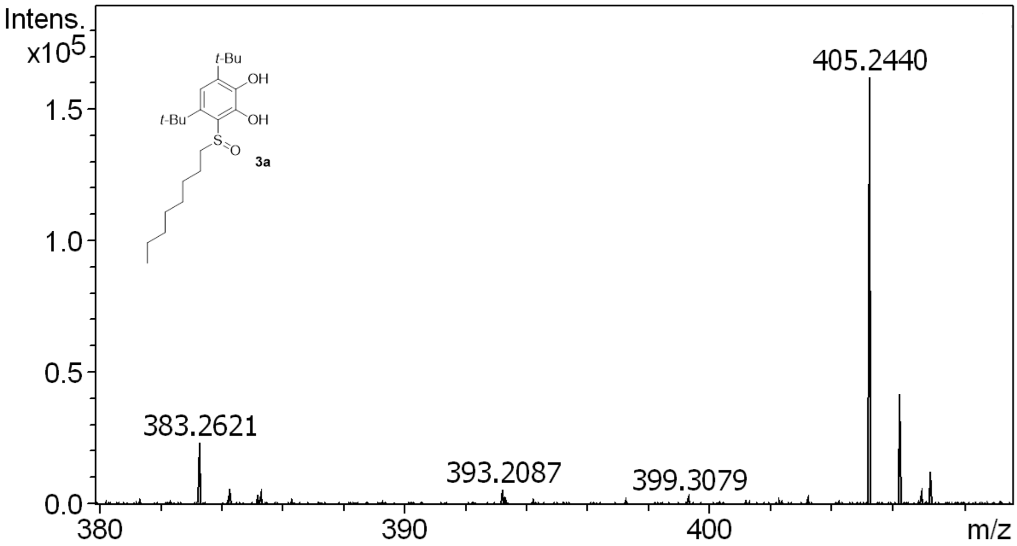

Meas. m/z	#	Ion Formula	m/z	err [ppm]	mSigma		Score	rdb	e⁻ Conf	N-Rule
405.2440	1	C22H38NaO3S	405.2434	-1.6	4.5	1	100.00	3.5	even	ok

Figure S23. HRMS spectra of **3a**.

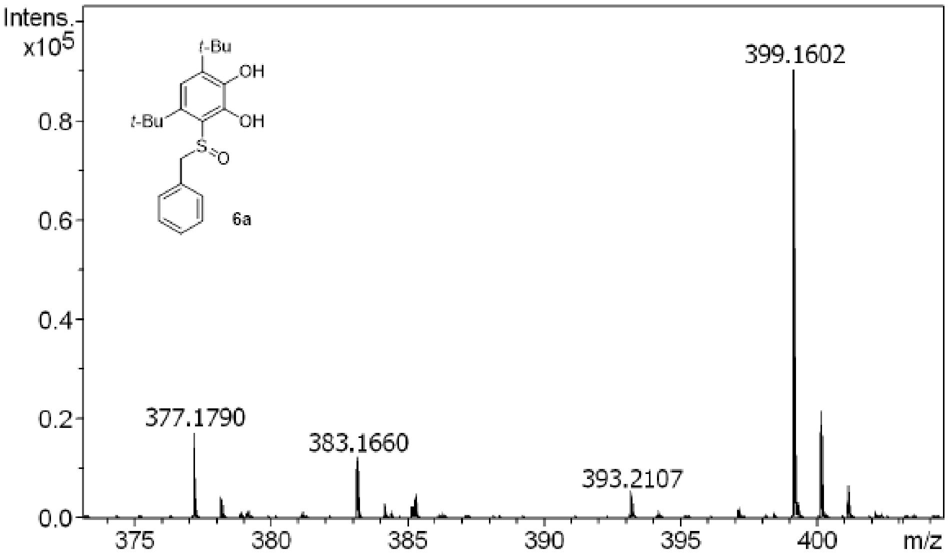

Meas. m/z	#	Ion Formula	m/z	err [ppm]	mSigma		Score	rdb	e⁻ Conf	N-Rule
399.1602	1	C21H28NaO4S	399.1601	-0.3	3.5	1	100.00	7.5	even	ok

Figure S24. HRMS spectra of **6a**.

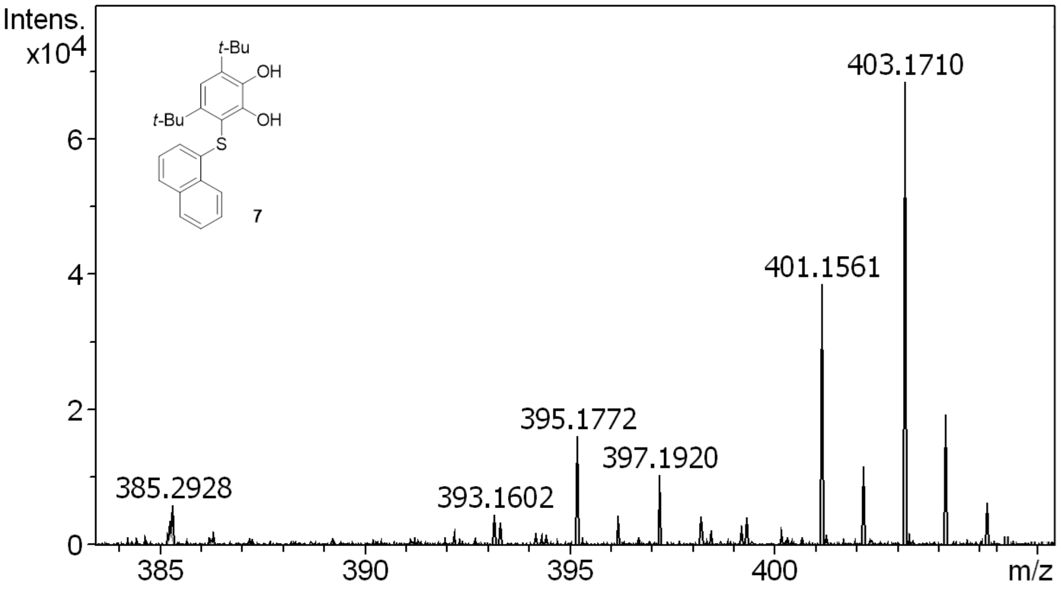

Meas. m/z	#	Ion Formula	m/z	err [ppm]	mSigma		Score	rdb	e⁻ Conf	N-Rule
403.1710	1	C24H28NaO2S	403.1702	-1.9	6.6	1	100.00	10.5	even	ok

Figure S25. HRMS spectra of **7**.

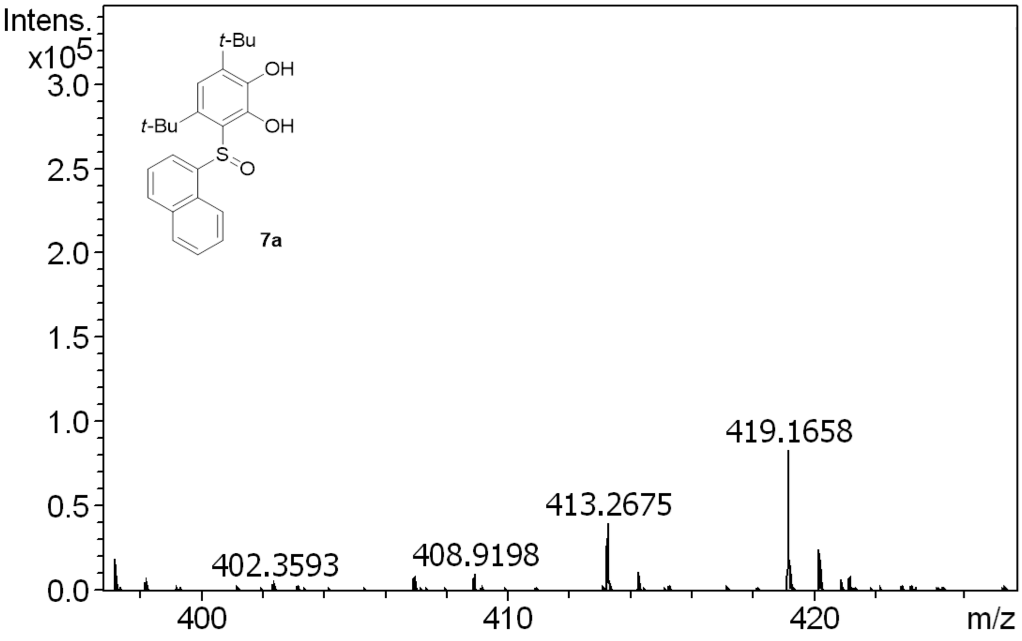

Meas. m/z	#	Ion Formula	m/z	err [ppm]	mSigma		Score	rdb	e⁻ Conf	N-Rule
419.1658	1	C24H28NaO3S	419.1651	-1.6	13.3	1	100.00	10.5	even	ok

Figure S26. HRMS spectra of **7a**.

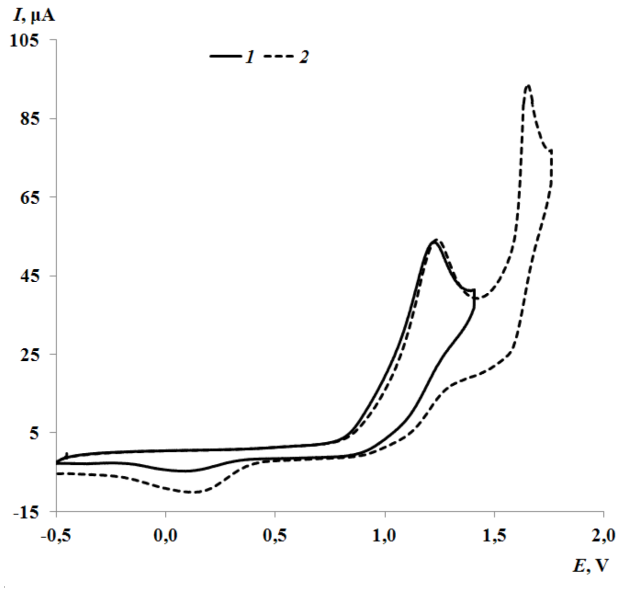

Figure S27. The CV curve of **2** at the potential range from –0.50 to 1.40 V (*curve 1*); from –0.50 to 1.80 V (*curve 2*) (CH_3CN, GC electrode, Ag/AgCl/KCl(sat.), 0.15 M *n*-Bu_4NClO_4, C = 3 mmol·L^{-1}).

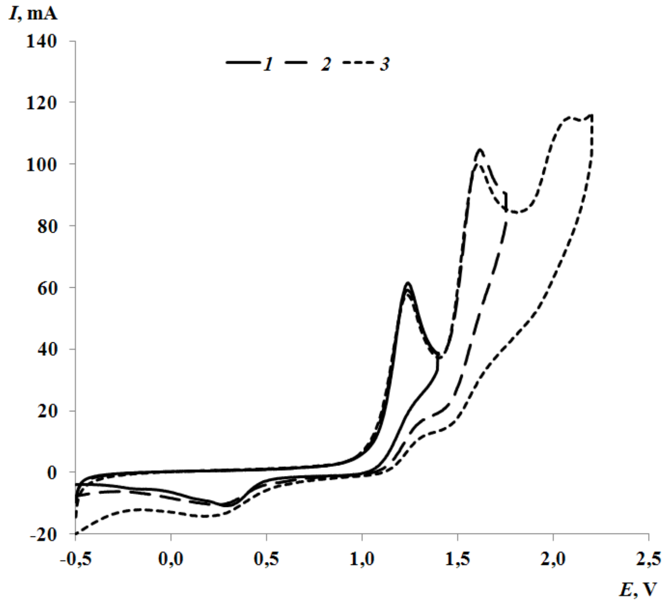

Figure S28. The CV curve of **2a** at the potential range from –0.50 to 1.40 V (*curve 1*); from –0.50 to 1.70 V (*curve 2*); from –0.50 to 2.20 V (*curve 3*) (CH_3CN, GC electrode, Ag/AgCl/KCl(sat.), 0.15 M *n*-Bu_4NClO_4, C = 3 mmol·L^{-1}).

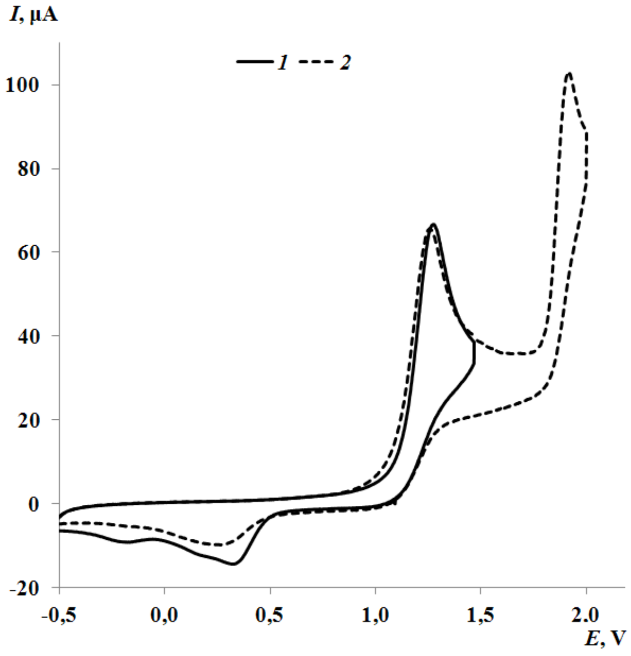

Figure S29. The CV curve of **3a** at the potential range from –0.50 to 1.50 V (*curve 1*); from –0.50 to 2.10 V (*curve 2*) (CH_3CN, GC electrode, Ag/AgCl/KCl(sat.), 0.15 M *n*-Bu_4NClO_4, C = 3 mmol·L^{-1}).

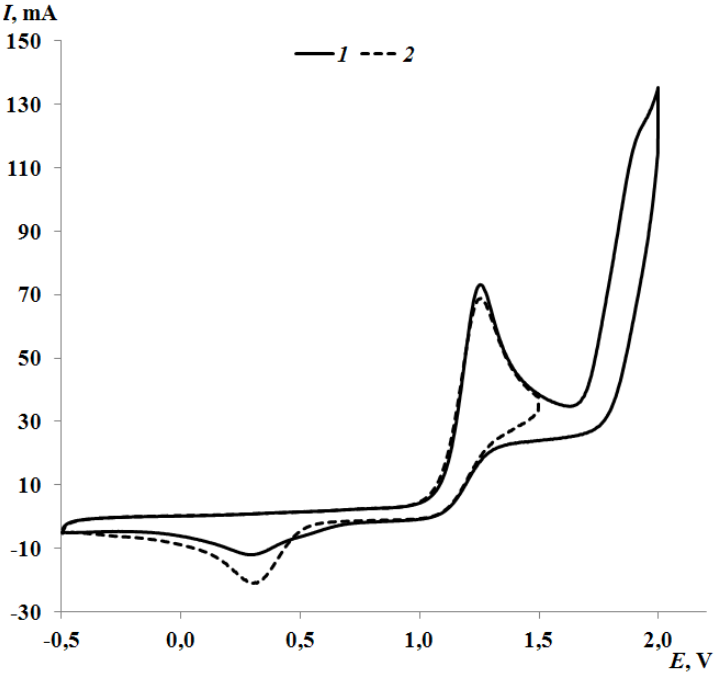

Figure S30. The CV curve of **4a** at the potential range from –0.5 to 1.5 V (*curve 1*); from –0.5 to 2.0 V (*curve 2*) (CH_3CN, GC electrode, Ag/AgCl/KCl(sat.), 0.15 M *n*-Bu_4NClO_4, C = 3 mmol·L^{-1}).

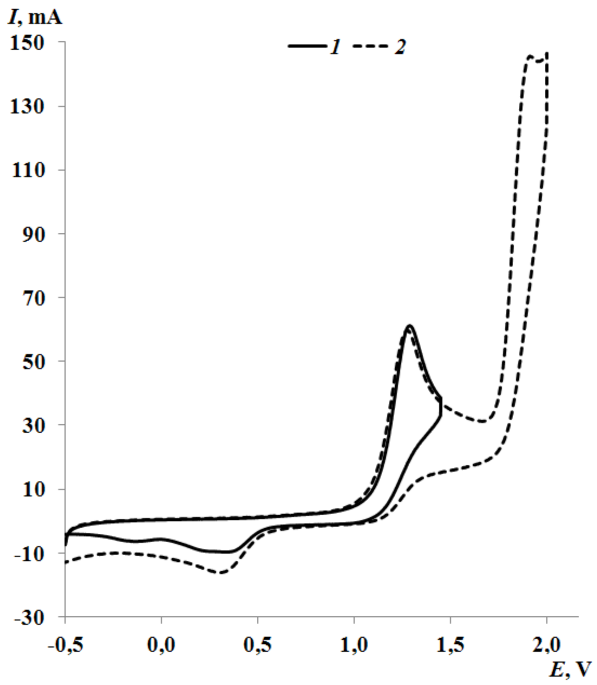

Figure S31. The CV curve of **6a** at the potential range from –0.50 to 1.50 V (*curve 1*); from –0.50 to 1.20 V (*curve 2*) (CH_3CN, GC electrode, Ag/AgCl/KCl(sat.), 0.15 M *n*-Bu_4NClO_4, C = 3 mmol·L^{-1}).

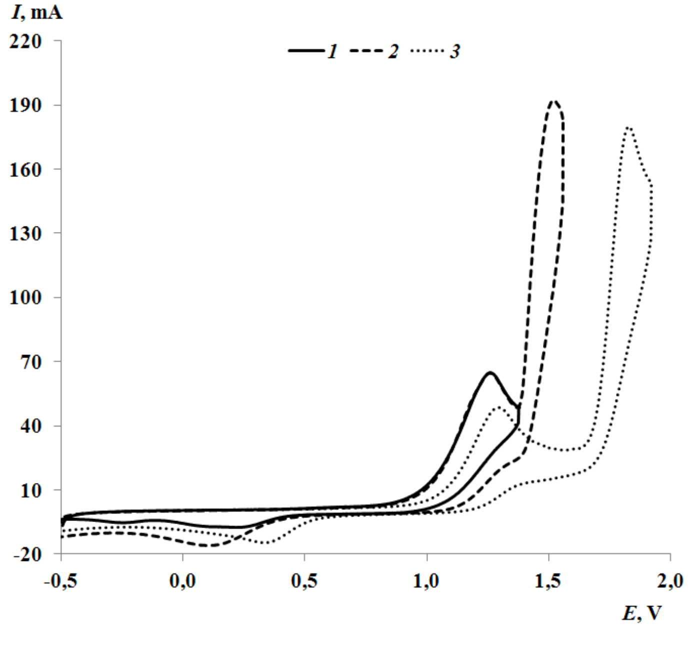

Figure S32. The CV curves of **7** and **7a** at the potential ranges: from –0.5 to 1.35 V for **7** (*curve 1*); from –0.5 to 1.5 V for **7** (*curve 2*); from –0.5 to 1.9 V for **7a** (*curve 3*); (CH_3CN, GC electrode, Ag/AgCl/KCl(sat.), 0.15 M *n*-Bu_4NClO_4, C = 3 mmol·L^{-1}).

S5. UV–vis spectroscopy

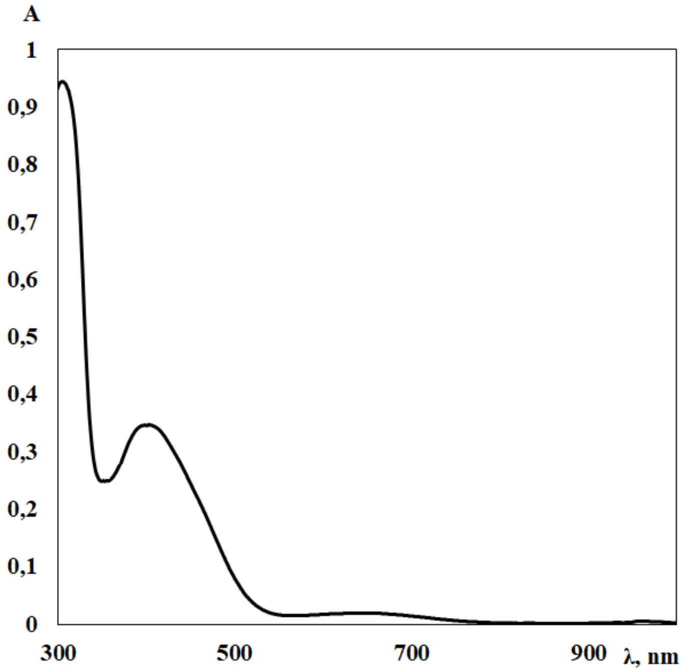

Figure S33. UV–vis spectra of the products of electrolysis of **5a** (MeCN, 293 K).

S6. References

1. Smolyaninov, I.V., Pitikova, O.V., Poddel'sky, A.I., Berberova, N.T. *Russ. Chem. Bull.* **2018**, *67*, 1857–1867. https://doi.org/10.1007/s11172-018-2299-9.
2. Smolyaninov, I.V., Burmistrova, D.A., Arsenyev, M.V., Polovinkina, M.A., Pomortseva, N.P., Fukin, G.K., Poddel'sky, A.I., Berberova, N.T. *Molecules* **2022**, *27 (10)*, 3169. https://doi.org/10.3390/molecules27103169.
3. Gordon, A.J., Ford, R.A. The chemist's companion. A Wiley interscience publication, New York, **1972**, 541 pp.
4. J. Koziskova, F. Hahn, J. Richter, J. Kozisek, *Acta Chimica Slovaca*, 2016, 9, 136.
5. Krause, L., Herbst-Irmer, R., Sheldrick G.M. & Stalke D., J. Appl. Cryst. 48 (2015) 3-10.
6. Sheldrick, G. M. SHELXS-2014, Program for Crystal Structure Solution; University of Göttingen: Göttingen, Germany, 2014.

7. Sheldrick, G. M. Crystal Structure Refinement with SHELXL. Acta Crystallogr., Sect. A: Found. Crystallogr. 2008, 64, 112−122.
8. Dolomanov, O. V.; Bourhis, L. J.; Gildea, R. J.; Howard, J. A. K.; Puschmann, H. OLEX2: a complete structure solution, refinement and analysis program. J. Appl. Crystallogr. 2009, 42, 339−341.
9. Bondet, V., Brand-Williams, W., Berset, C. *LWT - Food Sci.Technol.* **1997**, *30 (6)*, 609–615. https://doi.org/10.1006/fstl.1997.0240.
10. Smolyaninov, I.V., Antonova, N.A., Poddel'sky, A.I., Smolyaninova, S.A., Osipova, V.P., Berberova, N.T. *J. Organomet. Chem.* **2011**, *696 (13)*, 2611–2620. https://doi.org/10.1016/j.jorganchem.2011.04.004.
11. Re, R., Pellergrini, N., Proteggente, A., Pannala, A., Yang, M., Rice-Evans, C. *Free Radic. Biol. Med.* **1999**, *26*, 1231–1237. https://doi.org/10.1016/S0891-5849(98)00315-3.
12. Smolyaninov, I.V., Poddel'sky, A.I., Burmistrova, D.A., Voronina, Y.K., Pomortseva, N.P., Polovinkina, M.A., Almyasheva, N.R., Zamkova, M.A., Berberova, N.T., Eremenko, I.L. *Int. J. Mol. Sci.* **2023**, *24 (9)*, 8319. https://doi.org/10.3390/ijms24098319.
