# Supplementary material for: Unsymmetrical sulfoxides with sterically hindered catechol fragment: synthesis, structure, electrochemical properties, and antiradical activity
Source: Beilstein J Org Chem. 2026 Jun 1;22:828–37. doi: 10.3762/bjoc.22.65 (PMC13245472; doi:10.3762/bjoc.22.65)

checkCIF/PLATON report

Structure factors have been supplied for datablock(s) 109_autored

THIS REPORT IS FOR GUIDANCE ONLY. IF USED AS PART OF A REVIEW PROCEDURE FOR PUBLICATION, IT SHOULD NOT REPLACE THE EXPERTISE OF AN EXPERIENCED CRYSTALLOGRAPHIC REFEREE.

No syntax errors found.   CIF dictionary   Interpreting this report

Datablock: 109_autored

Bond precision:	C-C = 0.0026 A		Wavelength=1.54184
Cell:	a=15.0697(6)	b=8.5154(3)	c=19.8597(7)
	alpha=90	beta=108.128(4)	gamma=90
Temperature:	293 K		

	Calculated	Reported
Volume	2421.99(16)	2421.99(16)
Space group	P 21/c	P 1 21/c 1
Hall group	-P 2ybc	-P 2ybc
Moiety formula	C24 H28 O3 S [+ solvent]	C24 H28 O3 S
Sum formula	C24 H28 O3 S [+ solvent]	C24 H28 O3 S
Mr	396.52	396.52
Dx,g cm-3	1.087	1.087
Z	4	4
Mu (mm-1)	1.331	1.331
F000	848.0	848.0
F000'	851.58	
h,k,lmax	18,10,25	18,10,24
Nref	5071	5047
Tmin,Tmax	0.709,0.923	0.472,1.000
Tmin'	0.590	

Correction method= # Reported T Limits: Tmin=0.472 Tmax=1.000
AbsCorr = GAUSSIAN

Data completeness= 0.995   Theta(max)= 76.233

R(reflections)= 0.0469(4329)   wR2(reflections)= 0.1414(5047)

S = 1.045   Npar= 366

The following ALERTS were generated. Each ALERT has the format
test-name_ALERT_alert-type_alert-level.
Click on the hyperlinks for more details of the test.

Alert level C

```
PLAT230_ALERT_2_C Hirshfeld Test Diff for    S1       --O3       .         5.3 s.u.
PLAT242_ALERT_2_C Low    'MainMol' Ueq as Compared to Neighbors of          C7 Check
PLAT355_ALERT_3_C Long   O-H (X0.82,N0.98A)  O2       - H2       .        1.02 Ang.
```

Alert level G

```
PLAT002_ALERT_2_G Number of Distance or Angle Restraints on AtSite          35 Note
PLAT176_ALERT_4_G The CIF-Embedded .res File Contains SADI Records          19 Report
PLAT187_ALERT_4_G The CIF-Embedded .res File Contains RIGU Records           1 Report
PLAT199_ALERT_1_G Reported _cell_measurement_temperature ..... (K)         293 Check
PLAT200_ALERT_1_G Reported   _diffrn_ambient_temperature ..... (K)         293 Check
PLAT301_ALERT_3_G Main Residue  Disorder ..............(Resd    1)         36% Note
PLAT605_ALERT_4_G Largest Solvent Accessible VOID in the Structure         215 A**3
PLAT811_ALERT_5_G No ADDSYM Analysis: Too Many Excluded Atoms ....           ! Info
PLAT860_ALERT_3_G Number of Least-Squares Restraints ............          169 Note
PLAT868_ALERT_4_G ALERTS Due to the Use of _smtbx_masks Suppressed           ! Info
PLAT912_ALERT_4_G Missing # of FCF Reflections Above STh/L=  0.600          23 Note
PLAT969_ALERT_5_G The 'Henn et al.' R-Factor-gap value ..........        4.845 Note
              Predicted wR2: Based on SigI**2  2.91 or SHELX Weight 13.53
PLAT978_ALERT_2_G Number C-C Bonds with Positive Residual Density.           2 Info
PLAT992_ALERT_5_G Repd & Actual _reflns_number_gt Values Differ by           3 Check
```

0 **ALERT level A** = Most likely a serious problem - resolve or explain
0 **ALERT level B** = A potentially serious problem, consider carefully
3 **ALERT level C** = Check. Ensure it is not caused by an omission or oversight
14 **ALERT level G** = General information/check it is not something unexpected

2 ALERT type 1 CIF construction/syntax error, inconsistent or missing data
4 ALERT type 2 Indicator that the structure model may be wrong or deficient
3 ALERT type 3 Indicator that the structure quality may be low
5 ALERT type 4 Improvement, methodology, query or suggestion
3 ALERT type 5 Informative message, check

It is advisable to attempt to resolve as many as possible of the alerts in all categories. Often the minor alerts point to easily fixed oversights, errors and omissions in your CIF or refinement strategy, so attention to these fine details can be worthwhile. In order to resolve some of the more serious problems it may be necessary to carry out additional measurements or structure refinements. However, the purpose of your study may justify the reported deviations and the more serious of these should normally be commented upon in the discussion or experimental section of a paper or in the "special_details" fields of the CIF. checkCIF was carefully designed to identify outliers and unusual parameters, but every test has its limitations and alerts that are not important in a particular case may appear. Conversely, the absence of alerts does not guarantee there are no aspects of the results needing attention. It is up to the individual to critically assess their own results and, if necessary, seek expert advice.

Publication of your CIF in IUCr journals

A basic structural check has been run on your CIF. These basic checks will be run on all CIFs submitted for publication in IUCr journals (*Acta Crystallographica*, *Journal of Applied Crystallography*, *Journal of Synchrotron Radiation*); however, if you intend to submit to *Acta Crystallographica Section C* or *E* or *IUCrData*, you should make sure that full publication checks are run on the final version of your CIF prior to submission.

Publication of your CIF in other journals

Please refer to the *Notes for Authors* of the relevant journal for any special instructions relating to CIF submission.

PLATON version of 28/01/2025; check.def file version of 06/01/2025

Datablock 109_autored - ellipsoid plot

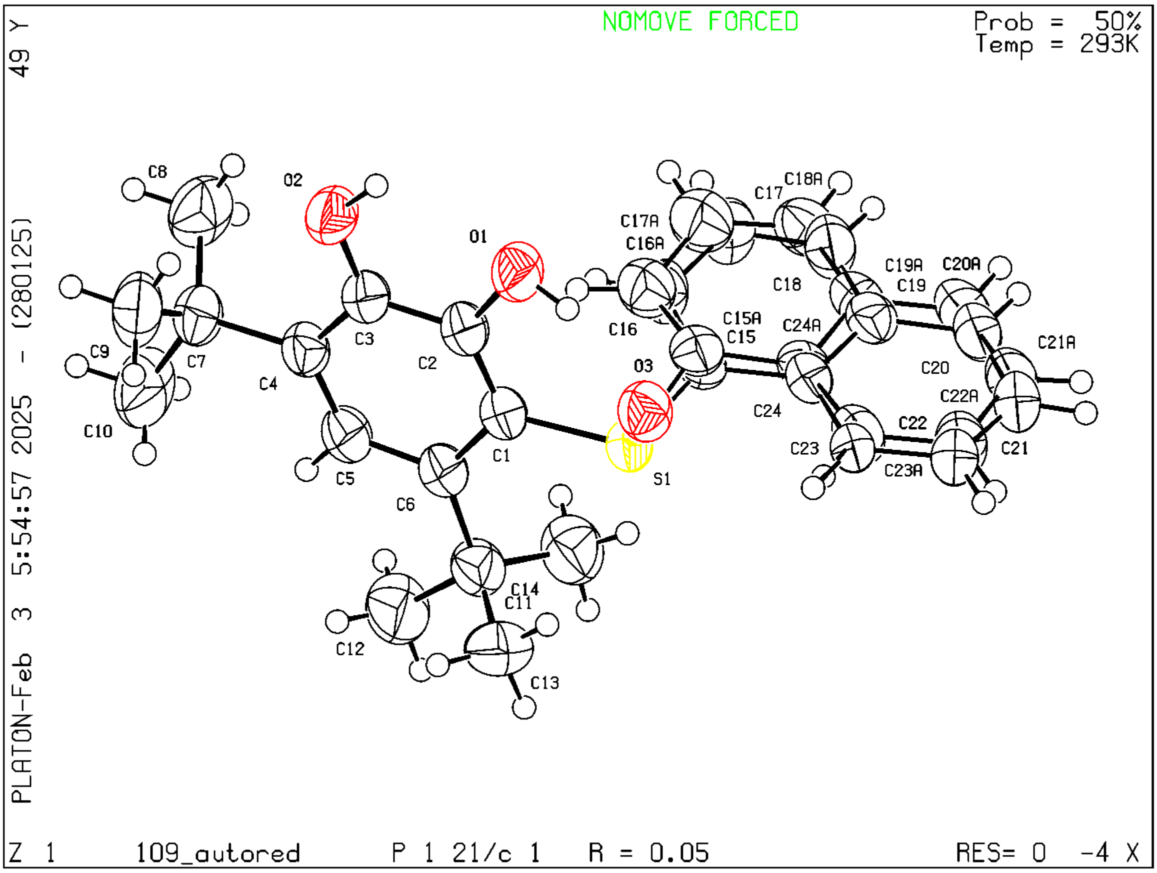

Supplement: File 2 — Crystallographic information files. [file Beilstein_J_Org_Chem-22-828-s002.zip › 7a_checkCIF.pdf]
