# Supplementary material for: Unsymmetrical sulfoxides with sterically hindered catechol fragment: synthesis, structure, electrochemical properties, and antiradical activity
Source: Beilstein J Org Chem. 2026 Jun 1;22:828–37. doi: 10.3762/bjoc.22.65 (PMC13245472; doi:10.3762/bjoc.22.65)

checkCIF/PLATON report

Structure factors have been supplied for datablock(s) sm_110_autored

THIS REPORT IS FOR GUIDANCE ONLY. IF USED AS PART OF A REVIEW PROCEDURE FOR PUBLICATION, IT SHOULD NOT REPLACE THE EXPERTISE OF AN EXPERIENCED CRYSTALLOGRAPHIC REFEREE.

No syntax errors found. CIF dictionary Interpreting this report

Datablock: sm_110_autored

```
Bond precision:   C-C = 0.0043 A           Wavelength=1.54184

Cell:            a=11.10107(17)     b=9.68288(17)     c=32.3713(5)
                 alpha=90           beta=90           gamma=90
Temperature:     100 K

                Calculated                    Reported
Volume          3479.60(10)                   3479.60(9)
Space group     P b c a                       P b c a
Hall group      -P 2ac 2ab                    -P 2ac 2ab
Moiety formula  C17 H28 O3 S                  C17 H28 O3 S
Sum formula     C17 H28 O3 S                  C17 H28 O3 S
Mr              312.45                        312.45
Dx,g cm-3       1.193                         1.193
Z               8                             8
Mu (mm-1)       1.708                         1.708
F000            1360.0                        1360.0
F000'           1366.19
h,k,lmax        13,12,40                      13,12,40
Nref            3641                          3617
Tmin,Tmax       0.604,0.699                   0.325,1.000
Tmin'           0.533

Correction method= # Reported T Limits: Tmin=0.325 Tmax=1.000
AbsCorr = GAUSSIAN

Data completeness= 0.993           Theta(max)= 76.067

                                                      wR2(reflections)=
R(reflections)= 0.0652( 3414)                         0.1707( 3617)

S = 1.067                  Npar= 206
```

The following ALERTS were generated. Each ALERT has the format
test-name_ALERT_alert-type_alert-level.
Click on the hyperlinks for more details of the test.

Alert level C

```
PLAT094_ALERT_2_C Ratio of Maximum / Minimum Residual Density ....       2.23 Report
PLAT340_ALERT_3_C Low Bond Precision on  C-C Bonds ...............    0.00425 Ang.
PLAT906_ALERT_3_C Large K Value in the Analysis of Variance ......     11.091 Check
PLAT911_ALERT_3_C Missing FCF Refl Between Thmin & STh/L=    0.600          3 Report
                2   6   0,    4   8   0,    0   0 12,
```

Alert level G

```
PLAT083_ALERT_2_G SHELXL Second Parameter in WGHT  Unusually Large      15.11 Why ?
PLAT883_ALERT_1_G Absent Datum for _atom_sites_solution_primary ..     Please Do !
PLAT912_ALERT_4_G Missing # of FCF Reflections Above STh/L=  0.600         21 Note
PLAT933_ALERT_2_G Number of HKL-OMIT Records in Embedded .res File          2 Note
                2   6   0,    4   8   0,
PLAT969_ALERT_5_G The 'Henn et al.' R-Factor-gap value ..........       6.205 Note
              Predicted wR2: Based on SigI**2  2.75 or SHELX Weight 16.00
PLAT978_ALERT_2_G Number C-C Bonds with Positive Residual Density.          7 Info
```

0 **ALERT level A** = Most likely a serious problem - resolve or explain
0 **ALERT level B** = A potentially serious problem, consider carefully
4 **ALERT level C** = Check. Ensure it is not caused by an omission or oversight
6 **ALERT level G** = General information/check it is not something unexpected

1 ALERT type 1 CIF construction/syntax error, inconsistent or missing data
4 ALERT type 2 Indicator that the structure model may be wrong or deficient
3 ALERT type 3 Indicator that the structure quality may be low
1 ALERT type 4 Improvement, methodology, query or suggestion
1 ALERT type 5 Informative message, check

It is advisable to attempt to resolve as many as possible of the alerts in all categories. Often the minor alerts point to easily fixed oversights, errors and omissions in your CIF or refinement strategy, so attention to these fine details can be worthwhile. In order to resolve some of the more serious problems it may be necessary to carry out additional measurements or structure refinements. However, the purpose of your study may justify the reported deviations and the more serious of these should normally be commented upon in the discussion or experimental section of a paper or in the "special_details" fields of the CIF. checkCIF was carefully designed to identify outliers and unusual parameters, but every test has its limitations and alerts that are not important in a particular case may appear. Conversely, the absence of alerts does not guarantee there are no aspects of the results needing attention. It is up to the individual to critically assess their own results and, if necessary, seek expert advice.

Publication of your CIF in IUCr journals

A basic structural check has been run on your CIF. These basic checks will be run on all CIFs submitted for publication in IUCr journals (*Acta Crystallographica*, *Journal of Applied Crystallography*, *Journal of Synchrotron Radiation*); however, if you intend to submit to *Acta Crystallographica Section C* or *E* or *IUCrData*, you should make sure that full publication checks are run on the final version of your CIF prior to submission.

Publication of your CIF in other journals

Please refer to the *Notes for Authors* of the relevant journal for any special instructions relating to CIF submission.

PLATON version of 02/02/2025; check.def file version of 02/02/2025

Datablock sm_110_autored - ellipsoid plot

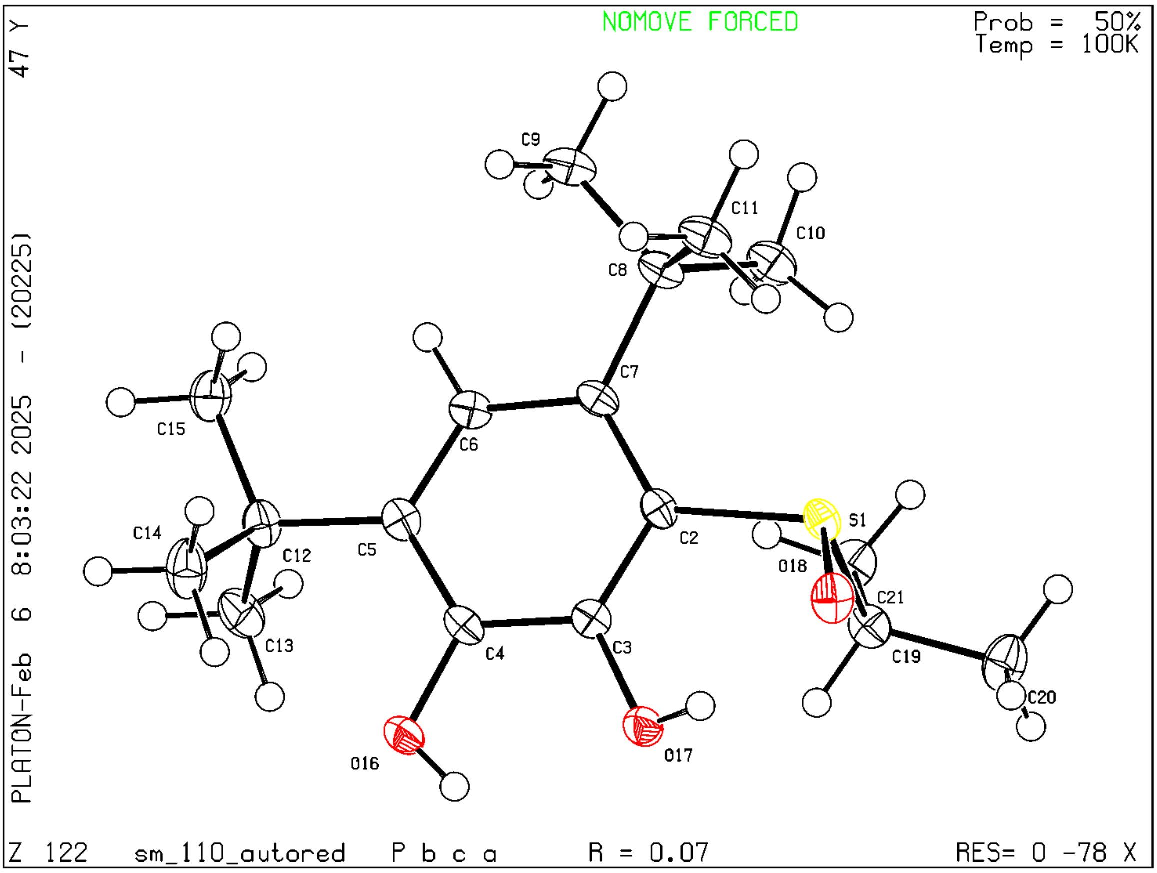

Supplement: File 2 — Crystallographic information files. [file Beilstein_J_Org_Chem-22-828-s002.zip › 1a_checkCIF.pdf]
