# Supplementary material for: Unsymmetrical sulfoxides with sterically hindered catechol fragment: synthesis, structure, electrochemical properties, and antiradical activity
Source: Beilstein J Org Chem. 2026 Jun 1;22:828–37. doi: 10.3762/bjoc.22.65 (PMC13245472; doi:10.3762/bjoc.22.65)

checkCIF/PLATON report

Structure factors have been supplied for datablock(s) lpg_a

THIS REPORT IS FOR GUIDANCE ONLY. IF USED AS PART OF A REVIEW PROCEDURE FOR PUBLICATION, IT SHOULD NOT REPLACE THE EXPERTISE OF AN EXPERIENCED CRYSTALLOGRAPHIC REFEREE.

No syntax errors found. CIF dictionary Interpreting this report

Datablock: lpg_a

Bond precision:	C-C = 0.0094 A		Wavelength=0.71073
Cell:	a=10.419(8)	b=29.386(16)	c=15.691(10)
	alpha=90	beta=101.33(3)	gamma=90
Temperature:	293 K		

	Calculated	Reported
Volume	4711(5)	4711(5)
Space group	P 21/n	P 1 21/n 1
Hall group	-P 2yn	-P 2yn
Moiety formula	8(C19 H30 O3 S), C6 H14	8(C19 H30 O3 S), C6 H14
Sum formula	C158 H254 O24 S8	C158 H254 O24 S8
Mr	2794.09	2794.08
Dx,g cm-3	0.985	0.985
Z	1	1
Mu (mm-1)	0.149	0.149
F000	1522.0	1522.0
F000'	1523.62	
h,k,lmax	12,35,19	12,35,19
Nref	8947	8917
Tmin,Tmax	0.968,0.988	0.573,0.747
Tmin'	0.901	

Correction method= # Reported T Limits: Tmin=0.573 Tmax=0.747
AbsCorr = MULTI SCAN

Data completeness= 0.997 Theta(max)= 25.682

R(reflections)= 0.1158(5979) wR2(reflections)= 0.2965(8917)

S = 1.151 Npar= 512

The following ALERTS were generated. Each ALERT has the format
test-name_ALERT_alert-type_alert-level.
Click on the hyperlinks for more details of the test.

Alert level B

```
PLAT049_ALERT_1_B Calculated Density Less Than 1.0 gcm-3 .........     0.9849 Check
```

Alert level C

```
PLAT082_ALERT_2_C High R1 Value ..................................       0.12 Report
PLAT084_ALERT_3_C High wR2 Value (i.e. > 0.25) ...................       0.30 Report
PLAT094_ALERT_2_C Ratio of Maximum / Minimum Residual Density ....       3.02 Report
PLAT220_ALERT_2_C NonSolvent   Resd 1  C   Ueq(max)/Ueq(min) Range        3.9 Ratio
PLAT220_ALERT_2_C NonSolvent   Resd 2  C   Ueq(max)/Ueq(min) Range        4.8 Ratio
PLAT230_ALERT_2_C Hirshfeld Test Diff for    C15       --C16      .       6.1 s.u.
PLAT230_ALERT_2_C Hirshfeld Test Diff for    C15       --C19      .       5.2 s.u.
PLAT234_ALERT_4_C Large Hirshfeld Difference C34       --C38      .      0.20 Ang.
PLAT234_ALERT_4_C Large Hirshfeld Difference C34       --C38A     .      0.19 Ang.
PLAT234_ALERT_4_C Large Hirshfeld Difference C37       --C38      .      0.16 Ang.
PLAT241_ALERT_2_C High   'MainMol' Ueq as Compared to Neighbors of        C16 Check
PLAT241_ALERT_2_C High   'MainMol' Ueq as Compared to Neighbors of        C18 Check
PLAT242_ALERT_2_C Low    'MainMol' Ueq as Compared to Neighbors of        C26 Check
PLAT242_ALERT_2_C Low    'MainMol' Ueq as Compared to Neighbors of        C30 Check
PLAT242_ALERT_2_C Low    'MainMol' Ueq as Compared to Neighbors of         S1 Check
PLAT242_ALERT_2_C Low    'MainMol' Ueq as Compared to Neighbors of         C7 Check
PLAT242_ALERT_2_C Low    'MainMol' Ueq as Compared to Neighbors of        C15 Check
PLAT260_ALERT_2_C Large Average Ueq of Residue Including       C1S      0.162 Check
PLAT340_ALERT_3_C Low Bond Precision on  C-C Bonds ...............    0.00939 Ang.
PLAT767_ALERT_4_C INS Embedded LIST 6 Instruction Should be LIST 4     Please Check
PLAT790_ALERT_4_C Centre of Gravity not Within Unit Cell: Resd.  #          1 Note
              C19 H30 O3 S
PLAT906_ALERT_3_C Large K Value in the Analysis of Variance ......     24.630 Check
PLAT906_ALERT_3_C Large K Value in the Analysis of Variance ......      4.378 Check
PLAT906_ALERT_3_C Large K Value in the Analysis of Variance ......      2.104 Check
PLAT911_ALERT_3_C Missing FCF Refl Between Thmin & STh/L=    0.600         25 Report
                1   2   0,   1   3   0,   2   3   0,   1   7   0,  -1   0   1,  -1   2   1,
                0   3   1,  -1   4   1,   0   4   1,  -2   0   2,   0   0   2,  -1   1   2,
                0   1   2,  -1   3   2,   0   3   2,  -1   0   3,  -1   2   3,   0   2   3,
               -1   3   3,   0   0   4,  -1   1   4,   7  19   6,   9   1  10,  -7  11  13,
               -3   2  15,
PLAT913_ALERT_3_C Missing # of Very Strong Reflections in FCF ....          8 Note
                0   2   0,   1   2   0,   1   3   0,   2   3   0,  -2   0   2,   0   1   2,
                0   2   3,   0   0   4,
PLAT918_ALERT_3_C Reflection(s) with I(obs) much Smaller I(calc) .          1 Check
PLAT934_ALERT_3_C Number of (Iobs-Icalc)/Sigma(W) > 10 Outliers ..          1 Check
               -1   4   4,
```

Alert level G

```
PLAT002_ALERT_2_G Number of Distance or Angle Restraints on AtSite         20 Note
PLAT003_ALERT_2_G Number of Uiso or U(i,j)  Restrained non-H-Atoms         19 Report
PLAT007_ALERT_5_G Number of Unrefined Donor-H Atoms ..............          4 Report
              H5    H6    H2    H3
PLAT063_ALERT_4_G Crystal Size Possibly too Large for Beam Size ..       0.70 mm
```

```
PLAT083_ALERT_2_G SHELXL Second Parameter in WGHT  Unusually Large      10.00 Why ?
PLAT171_ALERT_4_G The CIF-Embedded .res File Contains EADP Records          2 Report
PLAT172_ALERT_4_G The CIF-Embedded .res File Contains DFIX Records          6 Report
PLAT177_ALERT_4_G The CIF-Embedded .res File Contains DELU Records          2 Report
PLAT186_ALERT_4_G The CIF-Embedded .res File Contains ISOR Records          7 Report
PLAT192_ALERT_3_G A Non-default DELU Restraint Value for First Par     0.0010 Report
PLAT192_ALERT_3_G A Non-default DELU Restraint Value for SecondPar     0.0020 Report
PLAT192_ALERT_3_G A Non-default DELU Restraint Value for SecondPar     0.0200 Report
PLAT199_ALERT_1_G Reported _cell_measurement_temperature ..... (K)        293 Check
PLAT200_ALERT_1_G Reported   _diffrn_ambient_temperature ..... (K)        293 Check
PLAT300_ALERT_4_G Atom Site Occupancy of C1S        Constrained at       0.25 Check
PLAT300_ALERT_4_G Atom Site Occupancy of C2S        Constrained at       0.25 Check
PLAT300_ALERT_4_G Atom Site Occupancy of C3S        Constrained at       0.25 Check
PLAT300_ALERT_4_G Atom Site Occupancy of C4S        Constrained at       0.25 Check
PLAT300_ALERT_4_G Atom Site Occupancy of C5S        Constrained at       0.25 Check
PLAT300_ALERT_4_G Atom Site Occupancy of C6S        Constrained at       0.25 Check
PLAT300_ALERT_4_G Atom Site Occupancy of H1SA       Constrained at       0.25 Check
PLAT300_ALERT_4_G Atom Site Occupancy of H1SB       Constrained at       0.25 Check
PLAT300_ALERT_4_G Atom Site Occupancy of H1SC       Constrained at       0.25 Check
PLAT300_ALERT_4_G Atom Site Occupancy of H2SA       Constrained at       0.25 Check
PLAT300_ALERT_4_G Atom Site Occupancy of H2SB       Constrained at       0.25 Check
PLAT300_ALERT_4_G Atom Site Occupancy of H3SA       Constrained at       0.25 Check
PLAT300_ALERT_4_G Atom Site Occupancy of H3SB       Constrained at       0.25 Check
PLAT300_ALERT_4_G Atom Site Occupancy of H4SA       Constrained at       0.25 Check
PLAT300_ALERT_4_G Atom Site Occupancy of H4SB       Constrained at       0.25 Check
PLAT300_ALERT_4_G Atom Site Occupancy of H5SA       Constrained at       0.25 Check
PLAT300_ALERT_4_G Atom Site Occupancy of H5SB       Constrained at       0.25 Check
PLAT300_ALERT_4_G Atom Site Occupancy of H6SA       Constrained at       0.25 Check
PLAT300_ALERT_4_G Atom Site Occupancy of H6SB       Constrained at       0.25 Check
PLAT300_ALERT_4_G Atom Site Occupancy of H6SC       Constrained at       0.25 Check
PLAT301_ALERT_3_G Main Residue  Disorder .............(Resd    1)        17% Note
PLAT302_ALERT_4_G Anion/Solvent/Minor-Residue Disorder (Resd    3)       100% Note
PLAT411_ALERT_2_G Short Inter H...H Contact  H18B     ..H37C     .       1.71 Ang.
                                     1/2+x,3/2-y,-1/2+z  =      4_675 Check
PLAT412_ALERT_2_G Short Intra XH3 .. XHn     H31A     ..H38C     .       1.87 Ang.
                                                  x,y,z  =      1_555 Check
PLAT720_ALERT_4_G Number of Unusual/Non-Standard Labels ..........         14 Note
              H1SA    H1SB    H1SC    H2SA    H2SB    H3SA    H3SB    H4SA
              H4SB    H5SA    H5SB    H6SA    H6SB    H6SC
PLAT721_ALERT_1_G Bond    Calc     0.96000, Rep     0.97000 Dev...       0.01 Ang.
              C37A     -H37D           1_555   1_555 ........     #  117 Check
PLAT721_ALERT_1_G Bond    Calc     0.96000, Rep     0.97000 Dev...       0.01 Ang.
              C5S      -H5SA           1_555   1_555 ........     #  134 Check
PLAT721_ALERT_1_G Bond    Calc     0.97000, Rep     0.96000 Dev...       0.01 Ang.
              C6S      -H6SC           1_555   1_555 ........     #  139 Check
PLAT790_ALERT_4_G Centre of Gravity not Within Unit Cell: Resd.  #          2 Note
              C19 H30 O3 S
PLAT790_ALERT_4_G Centre of Gravity not Within Unit Cell: Resd.  #          3 Note
              C6 H14
PLAT860_ALERT_3_G Number of Least-Squares Restraints ............         139 Note
PLAT883_ALERT_1_G Absent Datum for _atom_sites_solution_primary ..     Please Do !
PLAT910_ALERT_3_G Missing FCF Reflection(s) Below Theta(Min)[Deg]=       2.11 Note
               0  2  0,   0  1  1,   0  2  1,
PLAT912_ALERT_4_G Missing # of FCF Reflections Above STh/L=  0.600          1 Note
PLAT933_ALERT_2_G Number of HKL-OMIT Records in Embedded .res File         16 Note
               -1  0  3,  -1  1  2,  -1  1  4,  -1  2  1,  -1  2  3,  -1  3  2,
               -1  3  3,  -1  4  1,   0  0  2,   0  2  1,   0  3  1,   0  3  2,
```

```
              0  4  1,   1  2  0,   1  7  0,   2  3  0,
PLAT941_ALERT_3_G Average HKL Measurement Multiplicity ..........        4.8 Low
PLAT969_ALERT_5_G The 'Henn et al.' R-Factor-gap value ..........      5.010 Note
              Predicted wR2: Based on SigI**2  5.92 or SHELX Weight 25.77
PLAT978_ALERT_2_G Number C-C Bonds with Positive Residual Density.          1 Info
```

```
  0 ALERT level A = Most likely a serious problem - resolve or explain
  1 ALERT level B = A potentially serious problem, consider carefully
 28 ALERT level C = Check. Ensure it is not caused by an omission or oversight
 52 ALERT level G = General information/check it is not something unexpected

  7 ALERT type 1 CIF construction/syntax error, inconsistent or missing data
 21 ALERT type 2 Indicator that the structure model may be wrong or deficient
 16 ALERT type 3 Indicator that the structure quality may be low
 35 ALERT type 4 Improvement, methodology, query or suggestion
  2 ALERT type 5 Informative message, check
```

It is advisable to attempt to resolve as many as possible of the alerts in all categories. Often the minor alerts point to easily fixed oversights, errors and omissions in your CIF or refinement strategy, so attention to these fine details can be worthwhile. In order to resolve some of the more serious problems it may be necessary to carry out additional measurements or structure refinements. However, the purpose of your study may justify the reported deviations and the more serious of these should normally be commented upon in the discussion or experimental section of a paper or in the "special_details" fields of the CIF. checkCIF was carefully designed to identify outliers and unusual parameters, but every test has its limitations and alerts that are not important in a particular case may appear. Conversely, the absence of alerts does not guarantee there are no aspects of the results needing attention. It is up to the individual to critically assess their own results and, if necessary, seek expert advice.

Publication of your CIF in IUCr journals

A basic structural check has been run on your CIF. These basic checks will be run on all CIFs submitted for publication in IUCr journals (*Acta Crystallographica*, *Journal of Applied Crystallography*, *Journal of Synchrotron Radiation*); however, if you intend to submit to *Acta Crystallographica Section C* or *E* or *IUCrData*, you should make sure that full publication checks are run on the final version of your CIF prior to submission.

Publication of your CIF in other journals

Please refer to the *Notes for Authors* of the relevant journal for any special instructions relating to CIF submission.

PLATON version of 04/06/2025; check.def file version of 30/05/2025

Datablock lpg_a - ellipsoid plot

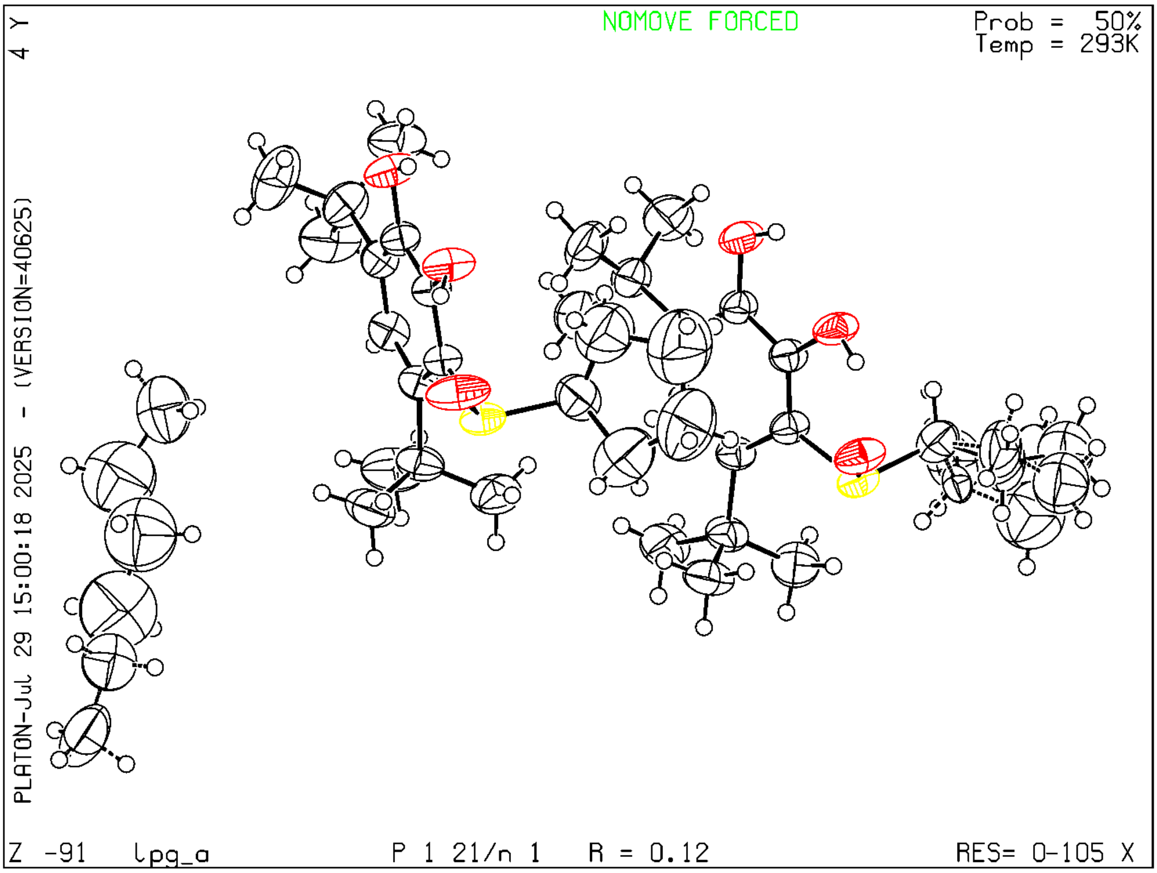

Supplement: File 2 — Crystallographic information files. [file Beilstein_J_Org_Chem-22-828-s002.zip › 4a_checkCIF.pdf]
