# Supplementary material for: Unsymmetrical sulfoxides with sterically hindered catechol fragment: synthesis, structure, electrochemical properties, and antiradical activity
Source: Beilstein J Org Chem. 2026 Jun 1;22:828–37. doi: 10.3762/bjoc.22.65 (PMC13245472; doi:10.3762/bjoc.22.65)

checkCIF/PLATON report

Structure factors have been supplied for datablock(s) 1_a

THIS REPORT IS FOR GUIDANCE ONLY. IF USED AS PART OF A REVIEW PROCEDURE FOR PUBLICATION, IT SHOULD NOT REPLACE THE EXPERTISE OF AN EXPERIENCED CRYSTALLOGRAPHIC REFEREE.

No syntax errors found. CIF dictionary Interpreting this report

Datablock: 1_a

Bond precision:	C-C = 0.0021 A		Wavelength=0.71073
Cell:	a=10.1078(9)	b=12.8261(12)	c=15.2212(15)
	alpha=90	beta=90	gamma=90
Temperature:	100 K		

	Calculated	Reported
Volume	1973.3(3)	1973.3(3)
Space group	P 21 21 21	P 21 21 21
Hall group	P 2ac 2ab	P 2ac 2ab
Moiety formula	C21 H28 O3 S	C21 H28 O3 S
Sum formula	C21 H28 O3 S	C21 H28 O3 S
Mr	360.49	360.49
Dx,g cm-3	1.213	1.213
Z	4	4
Mu (mm-1)	0.180	0.180
F000	776.0	776.0
F000'	776.82	
h,k,lmax	15,19,23	15,19,21
Nref	7552[4206]	6725
Tmin,Tmax	0.947,0.958	0.676,0.747
Tmin'	0.947	

Correction method= # Reported T Limits: Tmin=0.676 Tmax=0.747
AbsCorr = NONE

Data completeness= 1.60/0.89 Theta(max)= 33.181

R(reflections)= 0.0344(6394) wR2(reflections)= 0.0871(6725)

S = 1.046 Npar= 235

The following ALERTS were generated. Each ALERT has the format
test-name_ALERT_alert-type_alert-level.
Click on the hyperlinks for more details of the test.

Alert level C

```
STRVA01_ALERT_4_C           Flack test results are ambiguous.
           From the CIF: _refine_ls_abs_structure_Flack    0.500
           From the CIF: _refine_ls_abs_structure_Flack_su    0.060
PLAT094_ALERT_2_C Ratio of Maximum / Minimum Residual Density ....       2.10 Report
PLAT911_ALERT_3_C Missing FCF Refl Between Thmin & STh/L=    0.600         19 Report
                2  0  0,   1  1  0,   2  1  0,   0  2  0,   1  5  0,   0  2  1,
                0  3  1,   0  0  2,   1  0  2,   0  1  2,   1  0  3,   0  1  3,
                1  1  3,   0  2  3,   0  3  3,   0  0  4,   0  2  4,   0  2  5,
                2  0 16,
PLAT913_ALERT_3_C Missing # of Very Strong Reflections in FCF ....         10 Note
                2  0  0,   0  1  1,   0  0  2,   1  0  2,   0  1  2,   1  0  3,
                1  1  3,   0  0  4,   0  2  4,   0  2  5,
```

Alert level G

```
PLAT007_ALERT_5_G Number of Unrefined Donor-H Atoms .............          2 Report
              H1    H2
PLAT033_ALERT_4_G Flack x Value Deviates > 3.0 * sigma from Zero .      0.500 Note
PLAT883_ALERT_1_G No Info/Value for _atom_sites_solution_primary .     Please Do !
PLAT910_ALERT_3_G Missing # of FCF Reflection(s) Below Theta(Min).          1 Note
                0  1  1,
PLAT912_ALERT_4_G Missing # of FCF Reflections Above STh/L=  0.600        237 Note
PLAT933_ALERT_2_G Number of HKL-OMIT Records in Embedded .res File          1 Note
                1  5  0,
PLAT941_ALERT_3_G Average HKL Measurement Multiplicity ..........         4.0 Low
PLAT952_ALERT_5_G Calculated (ThMax) and CIF-Reported Lmax Differ.          2 Units
PLAT958_ALERT_1_G Calculated (ThMax) and Actual (FCF) Lmax Differ.          2 Units
PLAT978_ALERT_2_G Number C-C Bonds with Positive Residual Density.         19 Info
```

0 **ALERT level A** = Most likely a serious problem - resolve or explain
0 **ALERT level B** = A potentially serious problem, consider carefully
4 **ALERT level C** = Check. Ensure it is not caused by an omission or oversight
10 **ALERT level G** = General information/check it is not something unexpected

2 ALERT type 1 CIF construction/syntax error, inconsistent or missing data
3 ALERT type 2 Indicator that the structure model may be wrong or deficient
4 ALERT type 3 Indicator that the structure quality may be low
3 ALERT type 4 Improvement, methodology, query or suggestion
2 ALERT type 5 Informative message, check

It is advisable to attempt to resolve as many as possible of the alerts in all categories. Often the minor alerts point to easily fixed oversights, errors and omissions in your CIF or refinement strategy, so attention to these fine details can be worthwhile. In order to resolve some of the more serious problems it may be necessary to carry out additional measurements or structure refinements. However, the purpose of your study may justify the reported deviations and the more serious of these should normally be commented upon in the discussion or experimental section of a paper or in the "special_details" fields of the CIF. checkCIF was carefully designed to identify outliers and unusual parameters, but every test has its limitations and alerts that are not important in a particular case may appear. Conversely, the absence of alerts does not guarantee there are no aspects of the results needing attention. It is up to the individual to critically assess their own results and, if necessary, seek expert advice.

Publication of your CIF in IUCr journals

A basic structural check has been run on your CIF. These basic checks will be run on all CIFs submitted for publication in IUCr journals (*Acta Crystallographica*, *Journal of Applied Crystallography*, *Journal of Synchrotron Radiation*); however, if you intend to submit to *Acta Crystallographica Section C* or *E* or *IUCrData*, you should make sure that full publication checks are run on the final version of your CIF prior to submission.

Publication of your CIF in other journals

Please refer to the *Notes for Authors* of the relevant journal for any special instructions relating to CIF submission.

PLATON version of 29/11/2023; check.def file version of 14/09/2023

Datablock 1_a - ellipsoid plot

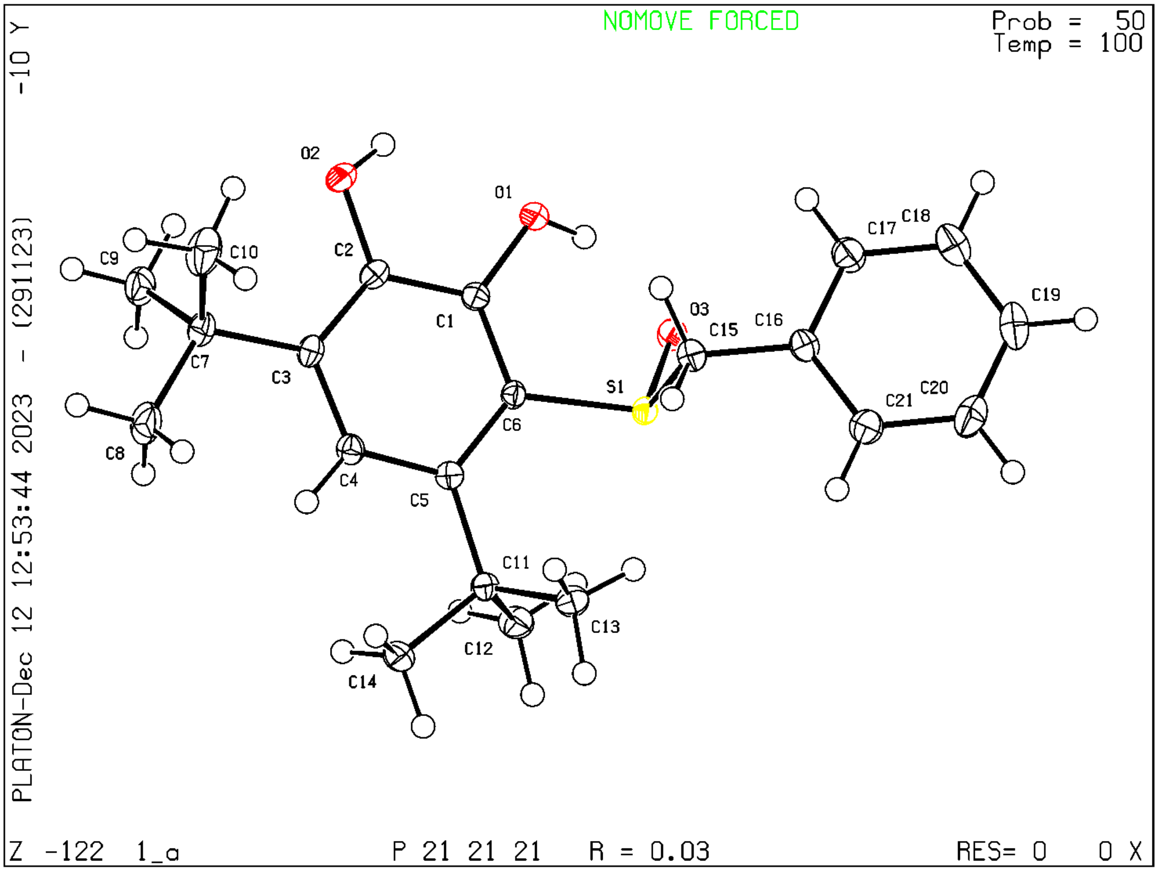

Supplement: File 2 — Crystallographic information files. [file Beilstein_J_Org_Chem-22-828-s002.zip › 6a_checkCIF.pdf]
